# Supplementary figures and images for: Strengthening of enterococcal biofilms by Esp
Source: PLoS Pathog. 2022 Sep 14;18(9):e1010829. doi: 10.1371/journal.ppat.1010829 (PMC9512215; doi:10.1371/journal.ppat.1010829)

S1 Figure

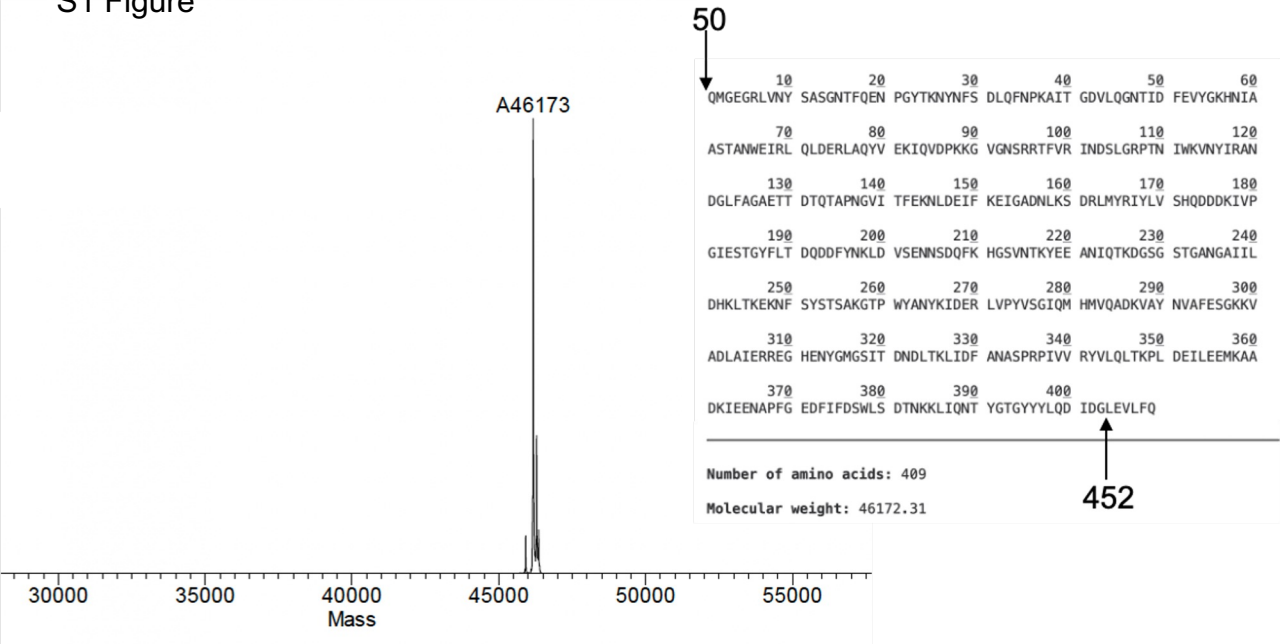

Supplement: S1 Fig — ESI-TOF mass spectrum of recombinant Esp452-His6 with the His6-tag removed by PreScission protease digestion. The sequence and predicted mass of the construct is indicated on the right. The sequence LEVLFQ at the C-terminus is from the PreScission protease cleavage site. Esp sequence numbers are indicated. (PDF) [file ppat.1010829.s001.pdf]

S2 Figure

A.

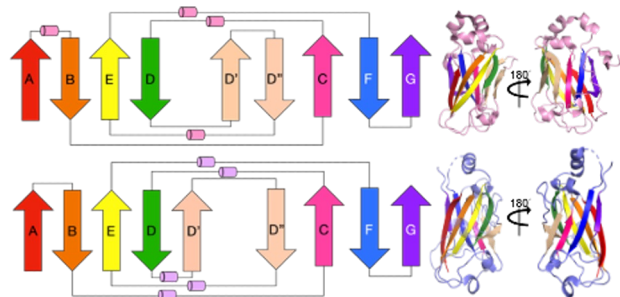

B.

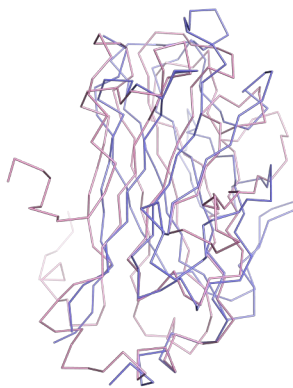

C.

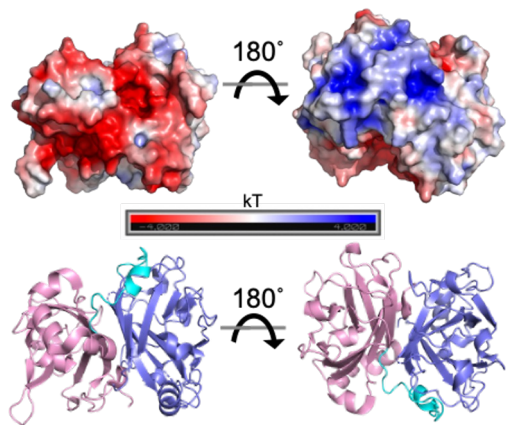

D.

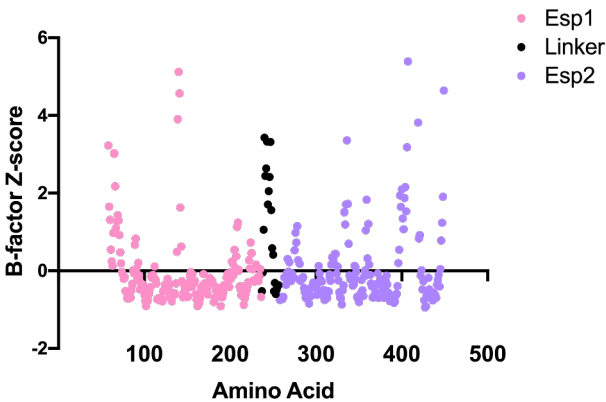

E.

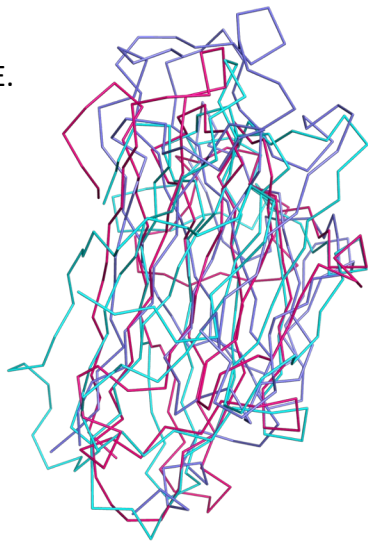

F..

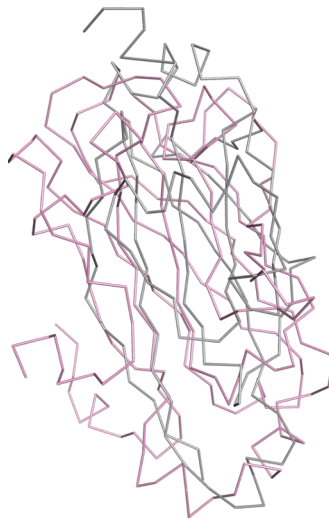

Supplement: S2 Fig — A. Topology of Esp1 (top) and Esp2 (bottom) shown in rainbow coloring, with similar coloring of the domains shown in ribbon representation at right. B. Esp1 (pink) and Esp2 (blue) superposed and depicted as Cα traces. C. Molecular surface of Esp452 viewed perpendicularly to the β-sheets. Surfaces with negative character shown in red, neutral in white, and positive in blue, ranging from -4.0 to 4.0 kT. Shown below is the same view in ribbon representation. D. Z-score for Cα atom B-factors, calculated with the following formula: Z = (Bx-Bavg)/s, where Bx is the B-factor of a given Cα atom, Bavg is the average B-factor of all Cα atoms in the structure, and s is the standard deviation of the Cα B-factors. Esp1 is shown in pink, Esp2 in lavender, and the linker in black. Certain loops and helices connecting β-strands, as well as the N- and C-termini, also have higher than average B-factors. E. Superposition of Esp2 (blue) with DEv-Ig domains of ClfA (pink) and ClfB (cyan), which bind fibrinogen. Rmsd of 3.8 and 3.9 Å, respectively, with Esp2 for 183 Cα. F. Superposition of Esp1 (pink) with DEv-Ig domain (gray) of Antigen I/II. Rmsd of 3.5 Å with Esp1 for 182 Cα. (PDF) [file ppat.1010829.s002.pdf]

S3 Figure

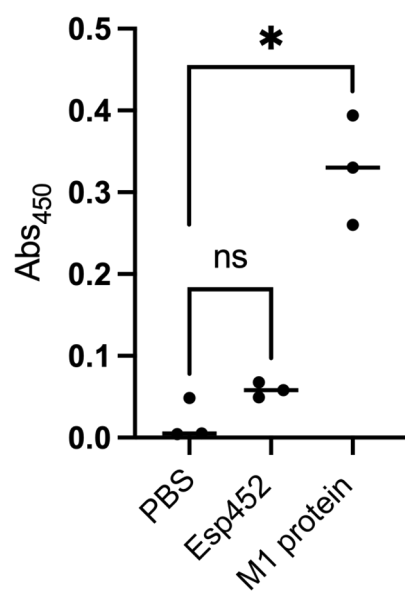

Supplement: S3 Fig — Wells of an ELISA plate were coated with fibrinogen, and equivalent molar amounts of Esp452-His6 or M1-His6 protein, or a PBS control was added to the wells. Bound His6-tagged proteins were quantified by ELISA using anti-His antibodies. The experiment was conducted one time in triplicate. Samples were compared by 1-Way ANOVA. *** p < 0.001. (PDF) [file ppat.1010829.s003.pdf]

S4 Figure

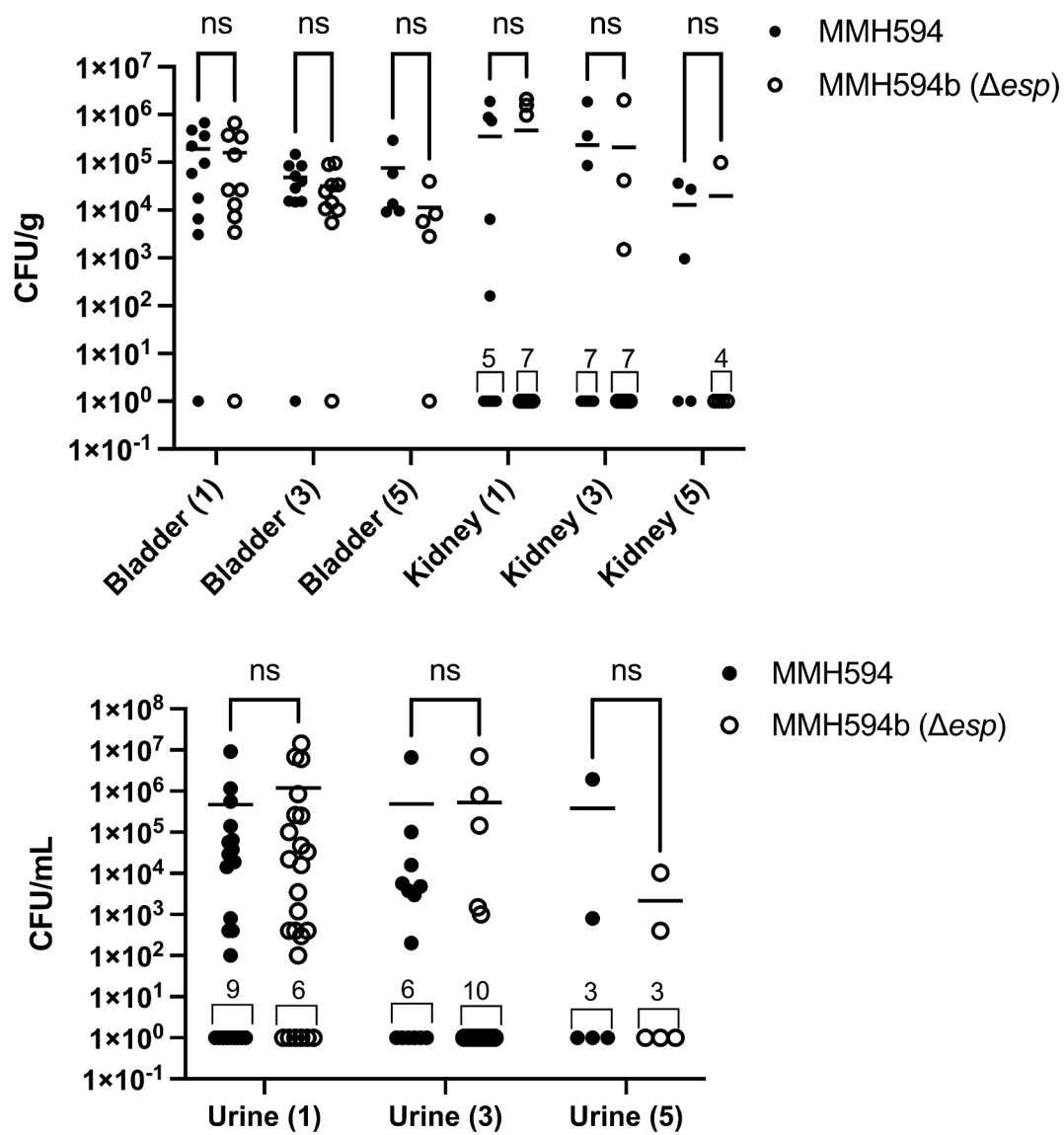

Supplement: S4 Fig — Mice were inoculated through the urethra with either MMH594 or MMH594b (Δesp). At 1, 3, or 5 days after inoculation, urine was collected, mice were sacrificed and tissues were homogenized in PBS and plated. Data are shown as CFU/g of tissue or CFU/mL of urine. The experiment was performed with five mice per sacrifice day, and independent experiments were performed twice for 1 and 3 days and once for 5 days. The mean is indicated with a horizontal line. Samples were compared by Fisher’s exact test. NS, p > 0.05. (PDF) [file ppat.1010829.s004.pdf]

S5 Figure

A.

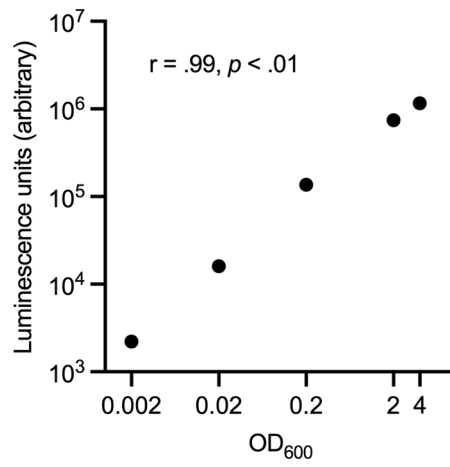

B.

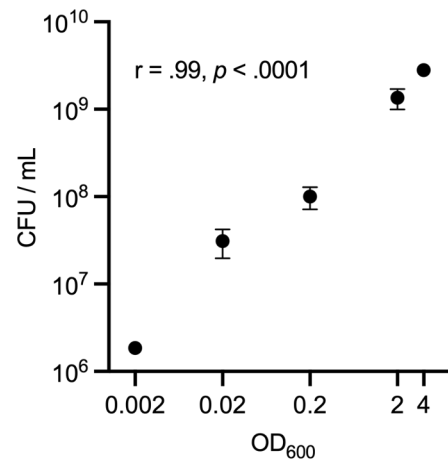

C.

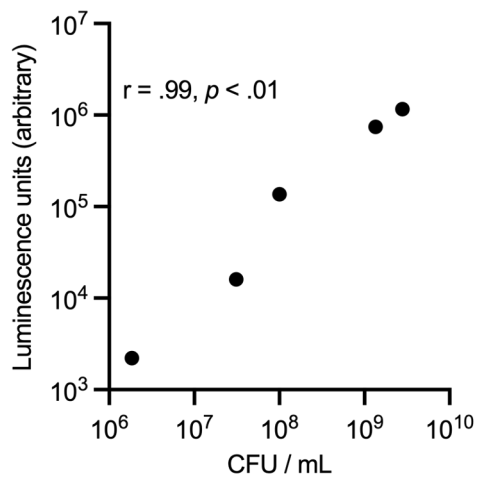

Supplement: S5 Fig — (A) Luminescence and (B) CFU/mL as a function of OD600, and (C) luminescence as a function of CFU/mL were measured for serial dilutions of MMH594. The Pearson correlation coefficient and corresponding p value are indicated on each graph. These relationships also apply to MMH594 (Δesp), as MMH594 and MMH594b (Δesp) were confirmed to have the same growth kinetics, as reported previously [44]. (PDF) [file ppat.1010829.s005.pdf]

S6 Figure

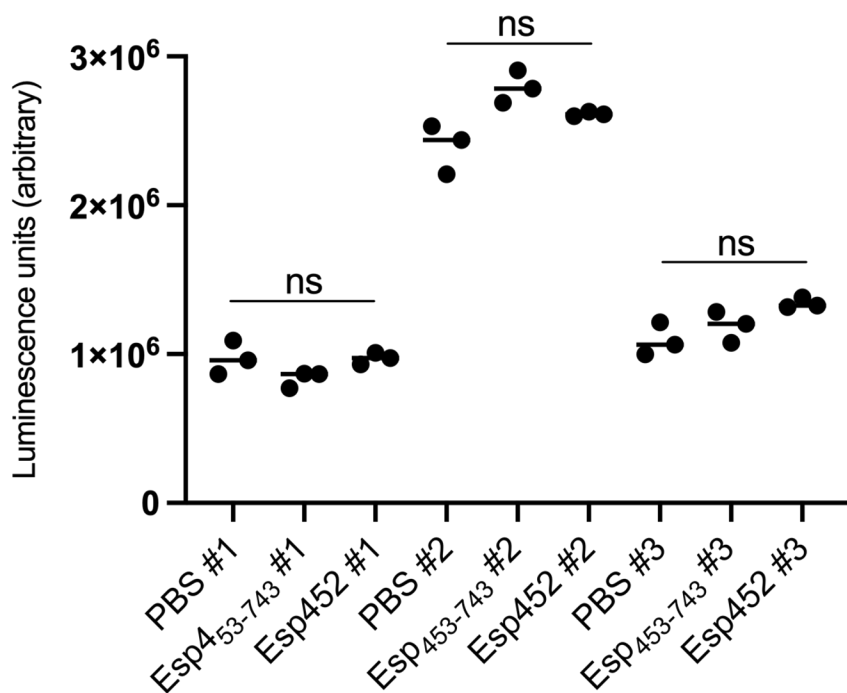

Supplement: S6 Fig — The total luminescence values of each well in three independent experiments are shown. Samples in each experiment were compared by Welch’s ANOVA with Dunnett T3 post hoc test. Total luminescence varied between experiments due to a variety of factors, including temperature at the time of measurement and age of the luminescence reagent. (PDF) [file ppat.1010829.s006.pdf]

S7 Figure

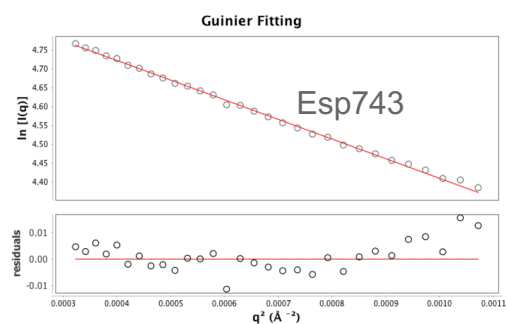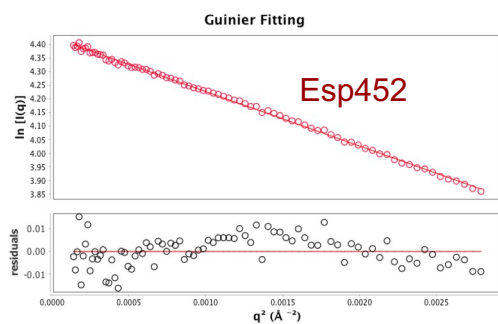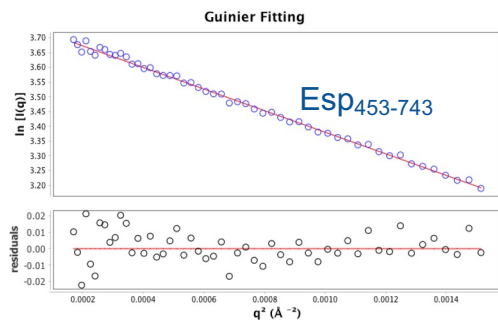

Supplement: S7 Fig — Guinier plots for experimental SEC-SAXS data for Esp743, Esp452, and Esp453-743. (PDF) [file ppat.1010829.s007.pdf]

S8 Figure

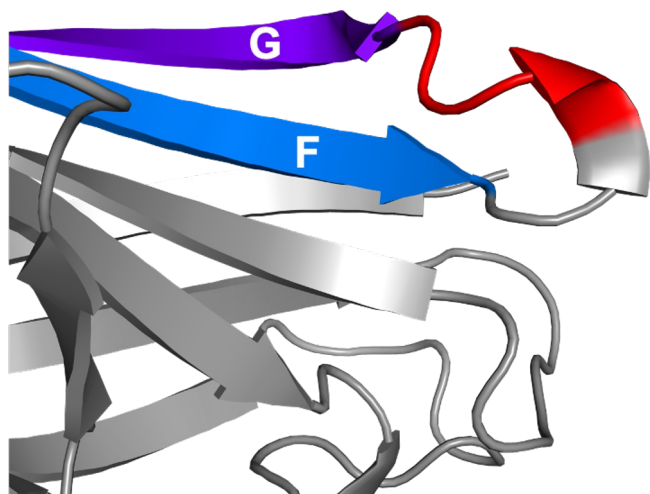

Supplement: S8 Fig — The DDDK sequence (red) is located on a loop between the Esp1 F and G β-strands (blue and purple, respectively). (PDF) [file ppat.1010829.s008.pdf]

S10 Figure

A.

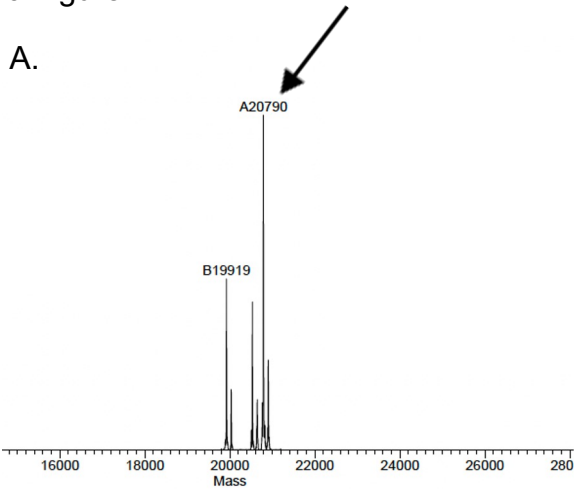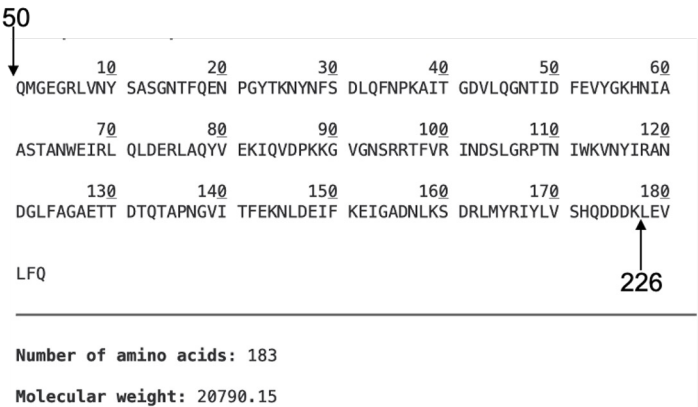

B.

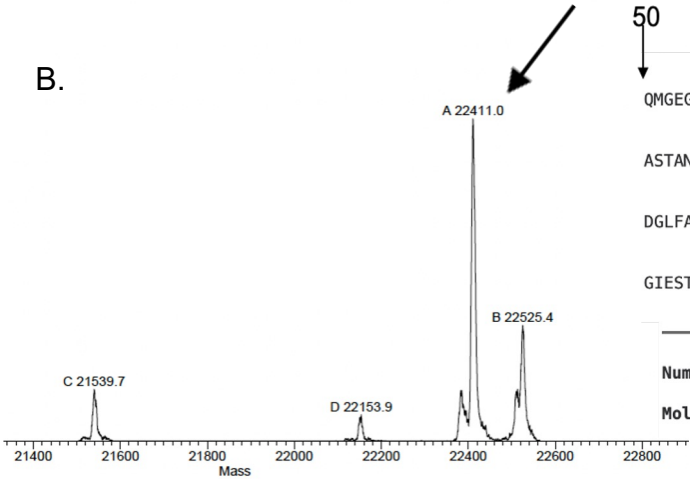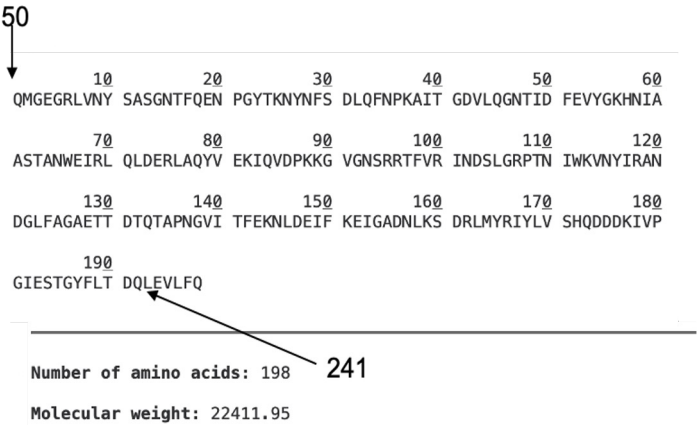

Supplement: S10 Fig — ESI-TOF spectra and sequences of (A) EspDDDK and (B) Esp1. ESI-TOF of recombinant Esp-His6 constructs in which the His6-tag was removed by PreScission protease digestion. The sequences and predicted masses of the constructs are indicated on the right. The C-terminal sequence LEVLFQ is from the PreScission protease cleavage site. The peaks corresponding to EspDDDK or Esp1 are indicated on the spectra with an arrow. (PDF) [file ppat.1010829.s010.pdf]

S11 Figure

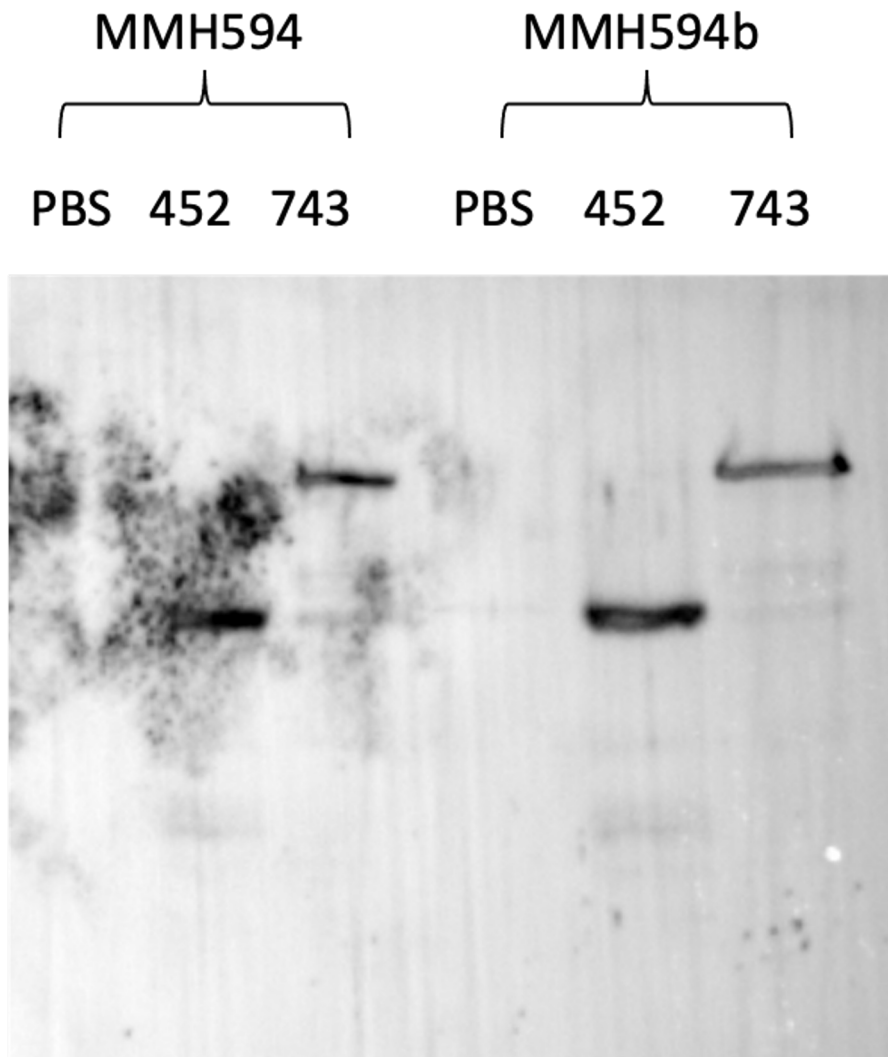

Supplement: S11 Fig — MMH594 and MMH594b (Δesp) biofilms were grown with Esp452, Esp743, or PBS. The biofilms were dissolved with NaCl, filtered, and assayed for the presence of Esp by western blot using anti-Esp452 polyclonal antibodies. (PDF) [file ppat.1010829.s011.pdf]

S12 Figure

A.

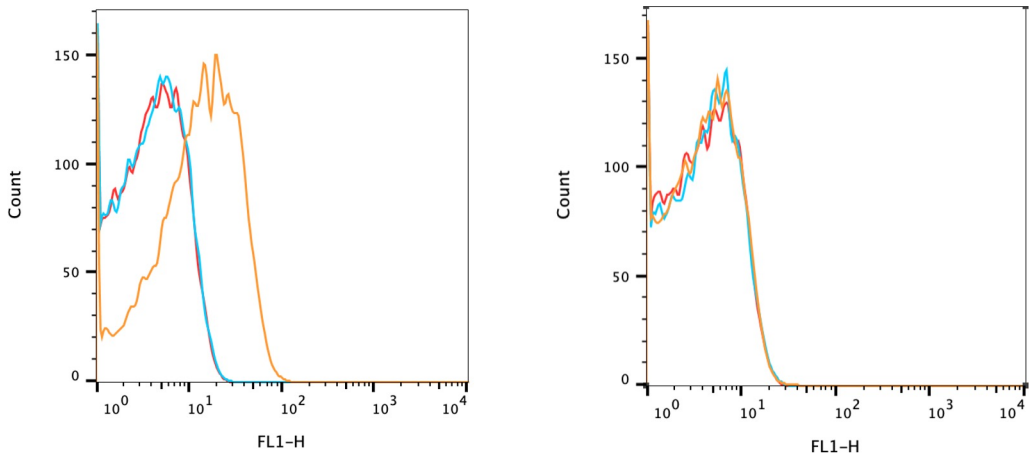

B.

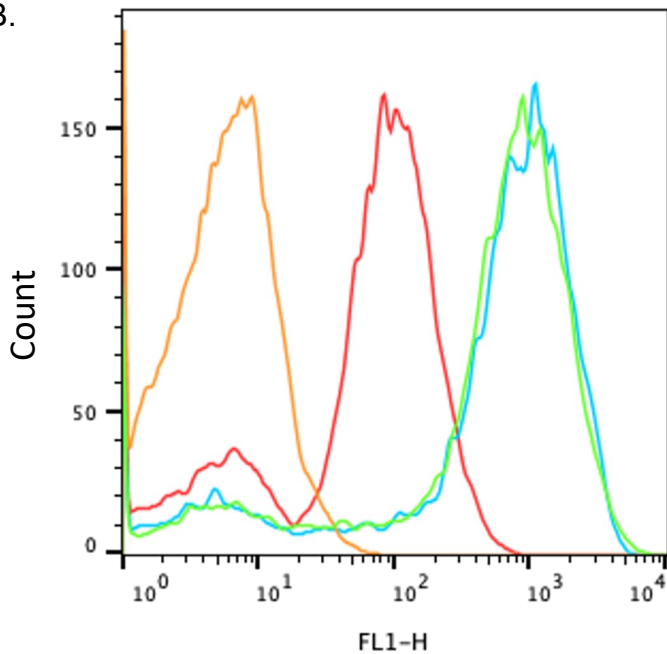

|  | Sample Name    | Subset Name | Count |
|--|----------------|-------------|-------|
|  | FA2-2+pEsp.008 | Ungated     | 10000 |
|  | FA2-2.006      | Ungated     | 10000 |
|  | 594+pEsp.004   | Ungated     | 10000 |
|  | 594-2.002      | Ungated     | 10000 |

Supplement: S12 Fig — A. MMH594 (left) and MMH594b (Δesp) (right) isolated from biofilms were incubated with rabbit anti-Esp452 antibodies followed by secondary antibodies conjugated to Alexa Fluor 488 (orange). Bacteria with no antibodies (red) and with secondary antibody only (blue) were measured to assess background fluorescence. Data were graphed with FloJo. B. FA2-2 (orange), FA2-2 (pEsp) (green), MMH594 (red), and MMH594 (pEsp) (cyan) isolated from biofilms were incubated with rabbit anti-Esp452 antibodies followed by secondary antibodies conjugated to Alexa Fluor 488. Data were graphed with FloJo. (PDF) [file ppat.1010829.s012.pdf]

S13 Figure

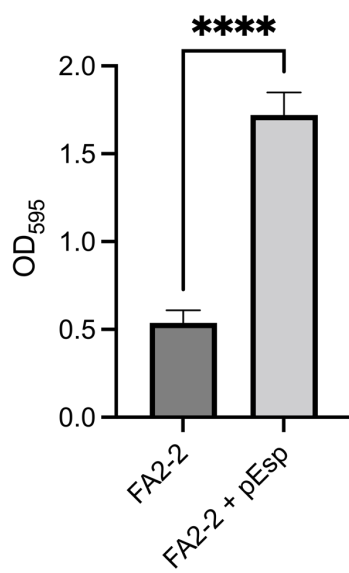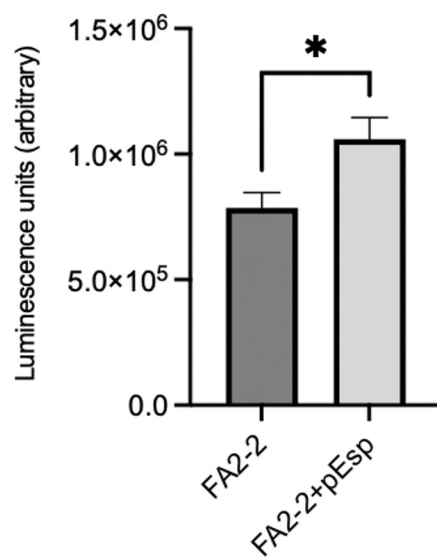

Supplement: S13 Fig — Crystal violet (A) and luminescence (B) measurements of biofilms produced by E. faecalis FA2-2 with and without pEsp. The experiments were conducted with sextuplicates and triplicates, respectively. Samples were compared by Student’s t-test. p < 0.05, *; p < .0001, ****. (PDF) [file ppat.1010829.s013.pdf]

S14 Figure

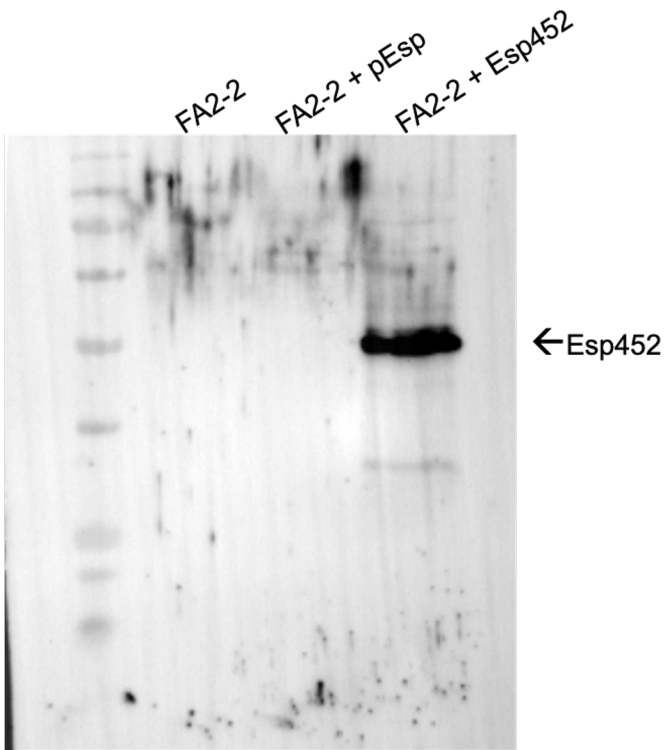

Supplement: S14 Fig — FA2-2 and FA2-2 (pEsp) biofilms were grown with PBS or Esp452. The biofilms were dissolved with NaCl, filtered, and assayed for the presence of Esp by western blot using anti-Esp452 polyclonal antibodies. (PDF) [file ppat.1010829.s014.pdf]

S15 Figure

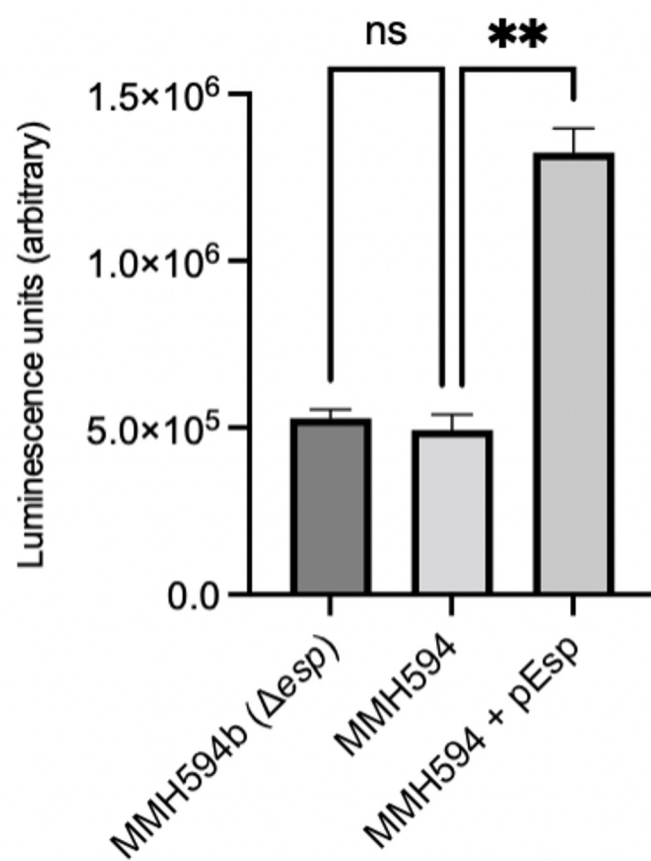

Supplement: S15 Fig — Luminescence of biofilm fractions of MMH594b (Δesp), MMH594, and MMH594 (pEsp). Welch’s ANOVA and D3 Dunnet’s post hoc test. p < 0.01, **. (PDF) [file ppat.1010829.s015.pdf]

S16 Figure

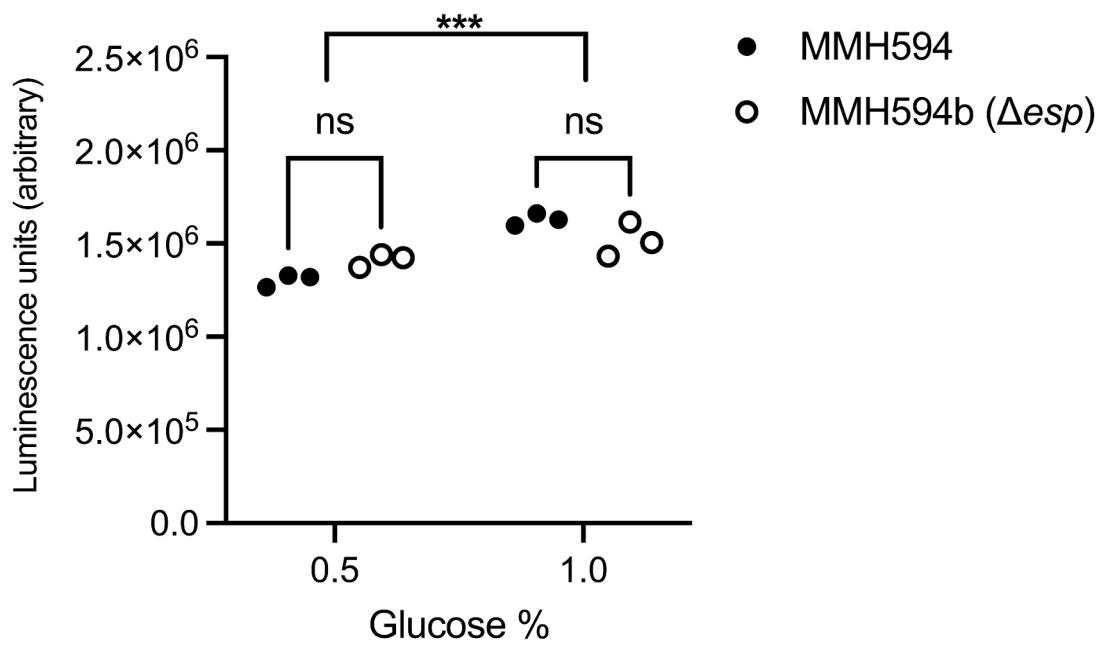

Supplement: S16 Fig — Luminescence of entire biofilm cultures, including the planktonic fractions, of MMH594 and MMH594b (Δesp) were measured. Samples were compared by 2-Way ANOVA and Tukey’s posthoc test. p < 0.001, ***. (PDF) [file ppat.1010829.s016.pdf]
