# Supplementary material for: Strengthening of enterococcal biofilms by Esp
Source: PLoS Pathog. 2022 Sep 14;18(9):e1010829. doi: 10.1371/journal.ppat.1010829 (PMC9512215; doi:10.1371/journal.ppat.1010829)
Supplement: S9 Fig — A. Products of digestion of Esp743 at 37°C for 3 h with the light chain of human enteropeptidase (EP) resolved by SDS-PAGE and InstantBlue-stained. At 3 h, the reaction was quenched before being applied to SDS-PAGE. The fragment designated by the arrowhead has a size matching EspDDDK. For the input sample, Esp743 and EP were incubated separately at 37°C for 3 h, quench solution was added to each, and the two were added together and immediately applied to SDS-PAGE. B. MALDI-TOF mass spectrogram of enteropeptidase-digested Esp743 and the theoretical sequence and weight of a hypothetical enteropeptidase cleavage product. The peak corresponding to the theoretical mass is indicated on the spectrum with an arrow. (PDF) [file ppat.1010829.s009.pdf]

S9 Figure

A.

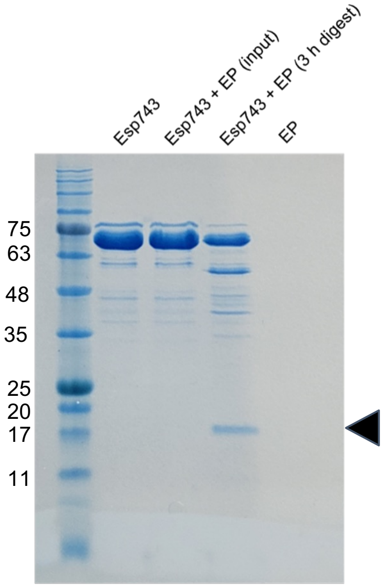

B.

Calc: 19715.84
Experimental: 19720.70

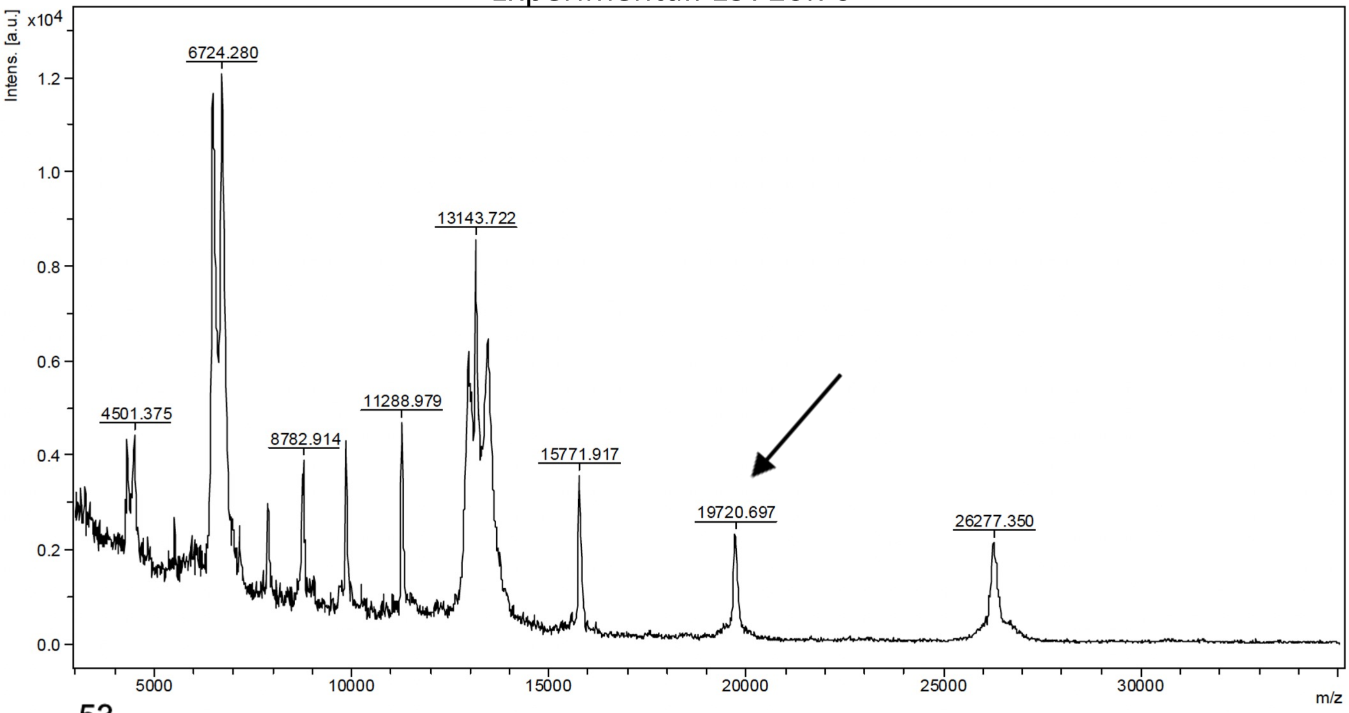

53

```
        10         20         30         40         50         60
EGRLANYSAS GNTFQENPGY TKNYNFSDLQ FNPKAITGDV LQGNTIDFEV YGKHNIAAST

        70         80         90        100        110        120
ANWEIRLQLD ERLAQYVEKI QVDPKKGVGN SRRTFVRIND SLGRPTNIWK VNYIRANDGL

       130        140        150        160        170
FAGAETTDTQ TAPNGVITFE KNLDEIFKEI GADNLKSDRL MYRIYLVSHQ DDDK
```

226

Number of amino acids: 174

Molecular weight: 19715.84
