# Supplementary material for: Strengthening of enterococcal biofilms by Esp
Source: PLoS Pathog. 2022 Sep 14;18(9):e1010829. doi: 10.1371/journal.ppat.1010829 (PMC9512215; doi:10.1371/journal.ppat.1010829)
Supplement: S1 Table — (PDF) [file ppat.1010829.s017.pdf]

S1 Table. Primers.

Construct	5’	3’
Esp743	ATATACCATGGGCAATGCACAAATGGGTGAAGGAAGATTAGCAAATTATTCTGC	CAGCACCTCGAGATTTTTACTTACAGTTACTGCTAAATCGGT
$Esp_{453\text{-}743}$	GATATACCATGGGCAATGCACAAATGGGTGAAGGAAGATTAGCAAATTATTCTGC	CAGCACCTCGAGATTTTTACTTACAGTTACTGCTAAATCGGT
Esp452	AGTTCTGTTCCAGGGGCCCGGATCCATGTTTGGAAAAAACAATAAGCATATG	GGCCGCAAGCTGGTGGTGCTCGAGTTAACCATCAATATCTTGCAAATAATAATAAC
Esp1	TTGACAGATCAGCTCGAGGTTCTGTTCCAGGGGCCC	GAACAGAACCTCGAGCTGATCTCTCAAAAAGTAACCAGTGC
Esp_{DDDK}	GAACAGAACCTCGAGTTTATCGTCATCTTGATGACTTACC	CATCAAGATGACGATAAACTCGAGGTTCTGTTCCAGGGGCCCGTC
