# Supplementary material for: Strengthening of enterococcal biofilms by Esp
Source: PLoS Pathog. 2022 Sep 14;18(9):e1010829. doi: 10.1371/journal.ppat.1010829 (PMC9512215; doi:10.1371/journal.ppat.1010829)
Supplement: S2 Table — (PDF) [file ppat.1010829.s018.pdf]

S2 Table. X-ray data collection and refinement statistics

	Esp	Esp – SeMet	Esp + Ca^{2+}
**Data collection**	ALS 5.0.2	ALS 12.3.1	APS 24-ID-E
Space group	P1	P1	P1
Cell dimensions			
a, b, c (Å)	39.9, 50.0, 51.2	40.0, 49.0, 51.0	39.9, 50.0, 51.2
α, β, γ (°)	107.4, 111.4, 90.4	111.0, 111.4, 91.7	107.3, 111.7, 90.1
Resolution (Å)	47.27-1.40 (1.42-1.40)[a]	26.49-2.29 (2.33-2.29)	45.11-2.14 (2.20-2.14)
R_{meas}[b]	.080 (.146)	.075 (.109)	.127 (.000)
$I / \sigma I$	11.3 (6.2)	35.2 (17.4)	5.4 (3.0)
Completeness (%)	85.4 (83.9)	98.0 (91.1)	92.8 (45.0)
$CC_{½}$[c]	.99 (.98)	.99 (.98)	.96 (.48)
Redundancy	3.2 (3.0)	3.7 (3.3)	1.9 (1.0)
**Refinement**			
Resolution (Å)	47.27-1.40 (1.42-1.40)		
Unique reflections	58,580 (2,996)		
R_{work} / R_{free}[d]	15.2/17.4 (16.3/21.9)		
No. atoms			
Protein	3025		
Ligand/ion	1		
Water	433		
B-factors (Å^2)			
Protein	11.9		
Ligand/ion	8.7		
Water	23.5		
R.m.s. deviations			
Bond lengths (Å)	.009		
Bond angles (°)	1.240		
Ramachandran plot			
favored (%)	99.7		
outliers (%)	0.3		
Clashscore	0		
Rotamer outliers (%)	0.6		
PDB code	6ORI		

[a]Values in parentheses are for the highest resolution shell.

[b]$R_{meas} = \sum_{hkl} \sqrt{\frac{n}{n-1}} \sum_{j=1}^{n} \left| I_{hkl\,j} - \langle I_{hkl} \rangle \right| / \sum_{hkl} \sum_{j} I_{hkl\,j}$

[c]$CC_{1/2}$ is the Pearson correlation coefficient between two random half datasets.

[d]$R_{work} = 100 \times \sum_{hkl} \left| F_{obs} - F_{calc} \right| / \sum_{hkl} F_{obs}$

Formulas for R_{work} and R_{free} are identical except 95% of the total number of reflections was used to calculate R_{work} whereas the remaining 5% of reflection was used to calculate R_{free}.
