# Supplementary material for: Strengthening of enterococcal biofilms by Esp
Source: PLoS Pathog. 2022 Sep 14;18(9):e1010829. doi: 10.1371/journal.ppat.1010829 (PMC9512215; doi:10.1371/journal.ppat.1010829)
Supplement: S3 Table — (PDF) [file ppat.1010829.s019.pdf]

S3 Table. Glycan Binding by Esp452

Chart ID	Streptavidin-488 (5ug/ml) Barcode #10127154	Average relative fluorescence units (Esp452 5 µg/mL)	Average relative fluorescence units (Esp452 50 µg/mL)
1	Gala-Sp8	102	28
2	Glca-Sp8	49	16
3	Mana-Sp8	22	6
4	GalNAca-Sp8	97	15
5	GalNAca-Sp15	11	7
6	Fuca-Sp8	89	42
7	Fuca-Sp9	60	24
8	Rhaa-Sp8	47	19
9	Neu5Aca-Sp8	64	27
10	Neu5Aca-Sp11	23	14
11	Neu5Acb-Sp8	27	8
12	Galb-Sp8	45	20
13	Glcb-Sp8	60	23
14	Manb-Sp8	73	53
15	GalNAcb-Sp8	46	21
16	GlcNAcb-Sp0	11	18
17	GlcNAcb-Sp8	32	14
18	GlcN(Gc)b-Sp8	102	20
19	Galb1-4GlcNAcb1-6(Galb1-4GlcNAcb1-3)GalNAca-Sp8	44	12
20	Galb1-4GlcNAcb1-6(Galb1-4GlcNAcb1-3)GalNAc-Sp14	50	39
21	GlcNAcb1-6(GlcNAcb1-4)(GlcNAcb1-3)GlcNAc-Sp8	78	24
22	6S(3S)Galb1-4(6S)GlcNAcb-Sp0	36	14
23	6S(3S)Galb1-4GlcNAcb-Sp0	11	11
24	(3S)Galb1-4(Fuca1-3)(6S)Glc-Sp0	69	-4
25	(3S)Galb1-4Glcb-Sp8	24	23
26	(3S)Galb1-4(6S)Glcb-Sp0	12	8
27	(3S)Galb1-4(6S)Glcb-Sp8	2	0
28	(3S)Galb1-3(Fuca1-4)GlcNAcb-Sp8	29	4
29	(3S)Galb1-3GalNAca-Sp8	5	6
30	(3S)Galb1-3GlcNAcb-Sp0	10	7
31	(3S)Galb1-3GlcNAcb-Sp8	2	-3
32	(3S)Galb1-4(Fuca1-3)GlcNAc-Sp0	69	44
33	(3S)Galb1-4(Fuca1-3)GlcNAc-Sp8	43	7
34	(3S)Galb1-4(6S)GlcNAcb-Sp0	17	9
35	(3S)Galb1-4(6S)GlcNAcb-Sp8	3	10
36	(3S)Galb1-4GlcNAcb-Sp0	28	7
37	(3S)Galb1-4GlcNAcb-Sp8	24	13
38	(3S)Galb-Sp8	25	6
39	(6S)(4S)Galb1-4GlcNAcb-Sp0	11	3
40	(4S)Galb1-4GlcNAcb-Sp8	10	5
41	(6P)Mana-Sp8	19	17
42	(6S)Galb1-4Glcb-Sp0	9	5
43	(6S)Galb1-4Glcb-Sp8	10	5
44	(6S)Galb1-4GlcNAcb-Sp8	10	8
45	(6S)Galb1-4(6S)Glcb-Sp8	22	2
46	Neu5Aca2-3(6S)Galb1-4GlcNAcb-Sp8	14	6
47	(6S)GlcNAcb-Sp8	16	6
48	Neu5,9Ac$_2$a-Sp8	6	0
49	Neu5,9Ac2a2-6Galb1-4GlcNAcb-Sp8	12	6
50	Mana1-6(Mana1-3)Manb1-4GlcNAcb1-4GlcNAcb-Sp12	15	9
51	Mana1-6(Mana1-3)Manb1-4GlcNAcb1-4GlcNAcb-Sp13	19	15
52	GlcNAcb1-2Mana1-6(GlcNAcb1-2Mana1-3)Manb1-4GlcNAcb1-4GlcNAcb-Sp12	22	15
53	GlcNAcb1-2Mana1-6(GlcNAcb1-2Mana1-3)Manb1-4GlcNAcb1-4GlcNAcb-Sp13	-1	2
54	Galb1-4GlcNAcb1-2Mana1-6(Galb1-4GlcNAcb1-2Mana1-3)Manb1-4GlcNAcb1-4GlcNAcb-Sp12	8	4
55	Neu5Aca2-6Galb1-4GlcNAcb1-2Mana1-6(Neu5Aca2-6Galb1-4GlcNAcb1-2Mana1-3)Manb1-4GlcNAcb1-4GlcNAcb-Sp12	1	4

56	Neu5Aca2-6Galb1-4GlcNAcb1-2Mana1-6(Neu5Aca2-6Galb1-4GlcNAcb1-2Man-a1-3)Manb1-4GlcNAcb1-4GlcNAcb-Sp21	21	9
57	Neu5Aca2-6Galb1-4GlcNAcb1-2Mana1-6(Neu5Aca2-6Galb1-4GlcNAcb1-2Mana1-3)Manb1-4GlcNAcb1-4GlcNAcb-Sp24	142	81
58	Fuca1-2Galb1-3GalNAcb1-3Gala-Sp9	18	6
59	Fuca1-2Galb1-3GalNAcb1-3Gala1-4Galb1-4Glcb-Sp9	9	10
60	Fuca1-2Galb1-3(Fuca1-4)GlcNAcb-Sp8	7	9
61	Fuca1-2Galb1-3GalNAca-Sp8	29	16
62	Fuca1-2Galb1-3GalNAca-Sp14	7	4
63	Fuca1-2Galb1-3GalNAcb1-4(Neu5Aca2-3)Galb1-4Glcb-Sp0	12	9
64	Fuca1-2Galb1-3GalNAcb1-4(Neu5Aca2-3)Galb1-4Glcb-Sp9	5	5
65	Fuca1-2Galb1-3GlcNAcb1-3Galb1-4Glcb-Sp8	8	8
66	Fuca1-2Galb1-3GlcNAcb1-3Galb1-4Glcb-Sp10	9	8
67	Fuca1-2Galb1-3GlcNAcb-Sp0	12	7
68	Fuca1-2Galb1-3GlcNAcb-Sp8	16	9
69	Fuca1-2Galb1-4(Fuca1-3)GlcNAcb1-3Galb1-4(Fuca1-3)GlcNAcb-Sp0	13	11
70	Fuca1-2Galb1-4(Fuca1-3)GlcNAcb1-3Galb1-4(Fuca1-3)GlcNAcb1-3Galb1-4(Fuca1-3)GlcNAcb-Sp0	45	32
71	Fuca1-2Galb1-4(Fuca1-3)GlcNAcb-Sp0	13	5
72	Fuca1-2Galb1-4(Fuca1-3)GlcNAcb-Sp8	15	8
73	Fuca1-2Galb1-4GlcNAcb1-3Galb1-4GlcNAcb-Sp0	0	2
74	Fuca1-2Galb1-4GlcNAcb1-3Galb1-4GlcNAcb1-3Galb1-4GlcNAcb-Sp0	-2	1
75	Fuca1-2Galb1-4GlcNAcb-Sp0	44	26
76	Fuca1-2Galb1-4GlcNAcb-Sp8	20	8
77	Fuca1-2Galb1-4Glcb-Sp0	28	10
78	Fuca1-2Galb-Sp8	24	7
79	Fuca1-3GlcNAcb-Sp8	12	6
80	Fuca1-4GlcNAcb-Sp8	55	2
81	Fucb1-3GlcNAcb-Sp8	19	9
82	GalNAca1-3(Fuca1-2)Galb1-3GlcNAcb-Sp0	51	23
83	GalNAca1-3(Fuca1-2)Galb1-4(Fuca1-3)GlcNAcb-Sp0	102	72
84	(3S)Galb1-4(Fuca1-3)Glcb-Sp0	20	8
85	GalNAca1-3(Fuca1-2)Galb1-4GlcNAcb-Sp0	9	5
86	GalNAca1-3(Fuca1-2)Galb1-4GlcNAcb-Sp8	5	0
87	GalNAca1-3(Fuca1-2)Galb1-4Glcb-Sp0	4	5
88	GlcNAcb1-3Galb1-3GalNAca-Sp8	17	6
89	GalNAca1-3(Fuca1-2)Galb-Sp8	5	6
90	GalNAca1-3(Fuca1-2)Galb-Sp18	18	12
91	GalNAca1-3GalNAcb-Sp8	6	8
92	GalNAca1-3Galb-Sp8	21	10
93	GalNAca1-4(Fuca1-2)Galb1-4GlcNAcb-Sp8	9	9
94	GalNAcb1-3GalNAca-Sp8	6	4
95	GalNAcb1-3(Fuca1-2)Galb-Sp8	14	1
96	GalNAcb1-3Gala1-4Galb1-4GlcNAcb-Sp0	16	9
97	GalNAcb1-4(Fuca1-3)GlcNAcb-Sp0	40	15
98	GalNAcb1-4GlcNAcb-Sp0	-5	-3
99	GalNAcb1-4GlcNAcb-Sp8	50	27
100	Gala1-2Galb-Sp8	44	14
101	Gala1-3(Fuca1-2)Galb1-3GlcNAcb-Sp0	20	13
102	Gala1-3(Fuca1-2)Galb1-3GlcNAcb-Sp8	125	81
103	Gala1-3(Fuca1-2)Galb1-4(Fuca1-3)GlcNAcb-Sp0	7	5
104	Gala1-3(Fuca1-2)Galb1-4(Fuca1-3)GlcNAcb-Sp8	68	41
105	Gala1-3(Fuca1-2)Galb1-4GlcNAc-Sp0	14	11
106	Gala1-3(Fuca1-2)Galb1-4Glcb-Sp0	11	9
107	Gala1-3(Fuca1-2)Galb-Sp8	16	2
108	Gala1-3(Fuca1-2)Galb-Sp18	10	15
109	Gala1-4(Gala1-3)Galb1-4GlcNAcb-Sp8	5	-9
110	Gala1-3GalNAca-Sp8	28	14
111	Gala1-3GalNAca-Sp16	98	43
112	Gala1-3GalNAcb-Sp8	17	4
113	Gala1-3Galb1-4(Fuca1-3)GlcNAcb-Sp8	3	9
114	Gala1-3Galb1-3GlcNAcb-Sp0	49	8
115	Gala1-3Galb1-4GlcNAcb-Sp8	6	2
116	Gala1-3Galb1-4Glcb-Sp0	74	16
117	Gala1-3Galb1-4Glc-Sp10	13	14
118	Gala1-3Galb-Sp8	15	8

119	Gala1-4(Fuca1-2)Galb1-4GlcNAcb-Sp8	-1	6
120	Gala1-4Galb1-4GlcNAcb-Sp0	19	6
121	Gala1-4Galb1-4GlcNAcb-Sp8	3	1
122	Gala1-4Galb1-4Glcb-Sp0	28	24
123	Gala1-4GlcNAcb-Sp8	31	28
124	Gala1-6Glcb-Sp8	33	15
125	Galb1-2Galb-Sp8	1	0
126	Galb1-3(Fuca1-4)GlcNAcb1-3Galb1-4(Fuca1-3)GlcNAcb-Sp0	6	7
127	Galb1-3GlcNAcb1-3Galb1-4(Fuca1-3)GlcNAcb-Sp0	6	1
128	Galb1-3(Fuca1-4)GlcNAc-Sp0	38	13
129	Galb1-3(Fuca1-4)GlcNAc-Sp8	12	7
130	Fuca1-4(Galb1-3)GlcNAcb-Sp8	14	0
131	Galb1-4GlcNAcb1-6GalNAca-Sp8	18	14
132	Galb1-4GlcNAcb1-6GalNAc-Sp14	7	13
133	GlcNAcb1-6(Galb1-3)GalNAca-Sp8	16	9
134	GlcNAcb1-6(Galb1-3)GalNAca-Sp14	4	-1
135	Neu5Aca2-6(Galb1-3)GalNAca-Sp8	3	4
136	Neu5Aca2-6(Galb1-3)GalNAca-Sp14	32	15
137	Neu5Acb2-6(Galb1-3)GalNAca-Sp8	9	4
138	Neu5Aca2-6(Galb1-3)GlcNAcb1-4Galb1-4Glcb-Sp10	-2	0
139	Galb1-3GalNAca-Sp8	21	7
140	Galb1-3GalNAca-Sp14	8	8
141	Galb1-3GalNAca-Sp16	30	9
142	Galb1-3GalNAcb-Sp8	9	10
143	Galb1-3GalNAcb1-3Gala1-4Galb1-4Glcb-Sp0	11	9
144	Galb1-3GalNAcb1-4(Neu5Aca2-3)Galb1-4Glcb-Sp0	1	2
145	Galb1-3GalNAcb1-4Galb1-4Glcb-Sp8	30	16
146	Galb1-3Galb-Sp8	37	20
147	Galb1-3GlcNAcb1-3Galb1-4GlcNAcb-Sp0	18	10
148	Galb1-3GlcNAcb1-3Galb1-4Glcb-Sp10	-3	0
149	Galb1-3GlcNAcb-Sp0	79	10
150	Galb1-3GlcNAcb-Sp8	29	14
151	Galb1-4(Fuca1-3)GlcNAcb-Sp0	10	6
152	Galb1-4(Fuca1-3)GlcNAcb-Sp8	12	2
153	Galb1-4(Fuca1-3)GlcNAcb1-3Galb1-4(Fuca1-3)GlcNAcb-Sp0	17	9
154	Galb1-4(Fuca1-3)GlcNAcb1-3Galb1-4(Fuca1-3)GlcNAcb1-3Galb1-4(Fuca1-3)GlcNAcb-Sp0	6	6
155	Galb1-4(6S)Glcb-Sp0	17	7
156	Galb1-4(6S)Glcb-Sp8	12	8
157	Galb1-4GalNAca1-3(Fuca1-2)Galb1-4GlcNAcb-Sp8	5	4
158	Galb1-4GalNAcb1-3(Fuca1-2)Galb1-4GlcNAcb-Sp8	6	0
159	Galb1-4GlcNAcb1-3GalNAca-Sp8	4	-1
160	Galb1-4GlcNAcb1-3GalNAc-Sp14	12	5
161	Galb1-4GlcNAcb1-3Galb1-4(Fuca1-3)GlcNAcb1-3Galb1-4(Fuca1-3)GlcNAcb-Sp0	9	6
162	Galb1-4GlcNAcb1-3Galb1-4GlcNAcb1-3Galb1-4GlcNAcb-Sp0	1	0
163	Galb1-4GlcNAcb1-3Galb1-4GlcNAcb-Sp0	59	38
164	Galb1-4GlcNAcb1-3Galb1-4Glcb-Sp0	6	-3
165	Galb1-4GlcNAcb1-3Galb1-4Glcb-Sp8	11	4
166	Galb1-4GlcNAcb1-6(Galb1-3)GalNAca-Sp8	3	4
167	Galb1-4GlcNAcb1-6(Galb1-3)GalNAc-Sp14	-3	2
168	Galb1-4GlcNAcb-Sp0	28	15
169	Galb1-4GlcNAcb-Sp8	21	16
170	Galb1-4GlcNAcb-Sp23	2	-5
171	Galb1-4Glcb-Sp0	9	12
172	Galb1-4Glcb-Sp8	13	9
173	GlcNAca1-3Galb1-4GlcNAcb-Sp8	5	4
174	GlcNAca1-6Galb1-4GlcNAcb-Sp8	1	3
175	GlcNAcb1-2Galb1-3GalNAca-Sp8	1	1
176	GlcNAcb1-6(GlcNAcb1-3)GalNAca-Sp8	-3	2
177	GlcNAcb1-6(GlcNAcb1-3)GalNAca-Sp14	12	6
178	GlcNAcb1-6(GlcNAcb1-3)Galb1-4GlcNAcb-Sp8	2	4
179	GlcNAcb1-3GalNAca-Sp8	11	2
180	GlcNAcb1-3GalNAca-Sp14	7	-1
181	GlcNAcb1-3Galb-Sp8	9	6
182	GlcNAcb1-3Galb1-4GlcNAcb-Sp0	4	5

183	GlcNAcb1-3Galb1-4GlcNAcb-Sp8	2	-1
184	GlcNAcb1-3Galb1-4GlcNAcb1-3Galb1-4GlcNAcb-Sp0	4	4
185	GlcNAcb1-3Galb1-4Glcb-Sp0	2	25
186	GlcNAcb1-4-MDPLys	2	3
187	GlcNAcb1-6(GlcNAcb1-4)GalNAca-Sp8	3	2
188	GlcNAcb1-4Galb1-4GlcNAcb-Sp8	-7	-3
189	GlcNAcb1-4GlcNAcb1-4GlcNAcb1-4GlcNAcb1-4GlcNAcb1-4GlcNAcb1-Sp8	25	7
190	GlcNAcb1-4GlcNAcb1-4GlcNAcb1-4GlcNAcb1-4GlcNAcb1-Sp8	6	2
191	GlcNAcb1-4GlcNAcb1-4GlcNAcb-Sp8	6	3
192	GlcNAcb1-6GalNAca-Sp8	28	1
193	GlcNAcb1-6GalNAca-Sp14	5	6
194	GlcNAcb1-6Galb1-4GlcNAcb-Sp8	1	-2
195	Glca1-4Glcb-Sp8	20	20
196	Glca1-4Glca-Sp8	31	14
197	Glca1-6Glca1-6Glcb-Sp8	27	9
198	Glcb1-4Glcb-Sp8	22	7
199	Glcb1-6Glcb-Sp8	27	10
200	G-ol-Sp8	20	15
201	GlcAa-Sp8	22	4
202	GlcAb-Sp8	138	93
203	GlcAb1-3Galb-Sp8	14	5
204	GlcAb1-6Galb-Sp8	29	10
205	KDNa2-3Galb1-3GlcNAcb-Sp0	5	6
206	KDNa2-3Galb1-4GlcNAcb-Sp0	8	4
207	Mana1-2Mana1-2Mana1-3Mana-Sp9	7	5
208	Mana1-2Mana1-6(Mana1-2Mana1-3)Mana-Sp9	-4	0
209	Mana1-2Mana1-3Mana-Sp9	11	7
210	Mana1-6(Mana1-2Mana1-3)Mana1-6(Mana1-2Mana1-3)Manb1-4GlcNAcb1-4GlcNAcb-Sp12	6	2
211	Mana1-2Mana1-6(Mana1-3)Mana1-6(Mana1-2Mana1-2Mana1-3)Manb1-4GlcNAcb1-4GlcNAcb-Sp12	28	7
212	Mana1-2Mana1-6(Mana1-2Mana1-3)Mana1-6(Mana1-2Mana1-2Mana1-3)Manb1-4GlcNAcb1-4GlcNAcb-Sp12	90	27
213	Mana1-6(Mana1-3)Mana-Sp9	5	2
214	Mana1-2Mana1-2Mana1-6(Mana1-3)Mana-Sp9	18	10
215	Mana1-6(Mana1-3)Mana1-6(Mana1-2Mana1-3)Manb1-4GlcNAcb1-4GlcNAcb-Sp12	150	77
216	Mana1-6(Mana1-3)Mana1-6(Mana1-3)Manb1-4GlcNAcb1-4GlcNAcb-Sp12	11	9
217	Manb1-4GlcNAcb-Sp0	-1	0
218	Neu5Aca2-3Galb1-4GlcNAcb1-3Galb1-4(Fuca1-3)GlcNAcb-Sp0	-8	-3
219	(3S)Galb1-4(Fuca1-3)(6S)GlcNAcb-Sp8	43	22
220	Fuca1-2(6S)Galb1-4GlcNAcb-Sp0	21	9
221	Fuca1-2Galb1-4(6S)GlcNAcb-Sp8	2	2
222	Fuca1-2(6S)Galb1-4(6S)Glcb-Sp0	15	6
223	Neu5Aca2-3Galb1-3GalNAca-Sp8	4	2
224	Neu5Aca2-3Galb1-3GalNAca-Sp14	11	1
225	GalNAcb1-4(Neu5Aca2-8Neu5Aca2-8Neu5Aca2-8Neu5Aca2-3)Galb1-4Glcb-Sp0	30	21
226	GalNAcb1-4(Neu5Aca2-8Neu5Aca2-8Neu5Aca2-3)Galb1-4Glcb-Sp0	96	50
227	Neu5Aca2-8Neu5Aca2-8Neu5Aca2-3Galb1-4Glcb-Sp0	6	3
228	GalNAcb1-4(Neu5Aca2-8Neu5Aca2-3)Galb1-4Glcb-Sp0	7	2
229	Neu5Aca2-8Neu5Aca2-8Neu5Aca-Sp8	4	-1
230	GalNAcb1-4(Neu5Aca2-3)Galb1-4GlcNAcb-Sp0	35	22
231	GalNAcb1-4(Neu5Aca2-3)Galb1-4GlcNAcb-Sp8	20	5
232	GalNAcb1-4(Neu5Aca2-3)Galb1-4Glcb-Sp0	-1	1
233	Neu5Aca2-3Galb1-3GalNAcb1-4(Neu5Aca2-3)Galb1-4Glcb-Sp0	3	1
234	Neu5Aca2-6(Neu5Aca2-3)GalNAca-Sp8	5	2
235	Neu5Aca2-3GalNAca-Sp8	11	2
236	Neu5Aca2-3GalNAcb1-4GlcNAcb-Sp0	2	-2
237	Neu5Aca2-3Galb1-3(6S)GlcNAc-Sp8	171	95
238	Neu5Aca2-3Galb1-3(Fuca1-4)GlcNAcb-Sp8	-2	-8
239	Neu5Aca2-3Galb1-3(Fuca1-4)GlcNAcb1-3Galb1-4(Fuca1-3)GlcNAcb-Sp0	2	0
240	Neu5Aca2-3Galb1-4(Neu5Aca2-3Galb1-3)GlcNAcb-Sp8	45	0

241	Neu5Aca2-3Galb1-3(6S)GalNAca-Sp8	6	3
242	Neu5Aca2-6(Neu5Aca2-3Galb1-3)GalNAca-Sp8	2	3
243	Neu5Aca2-6(Neu5Aca2-3Galb1-3)GalNAca-Sp14	29	12
244	Neu5Aca2-3Galb-Sp8	9	9
245	Neu5Aca2-3Galb1-3GalNAcb1-3Gala1-4Galb1-4Glcb-Sp0	20	11
246	Neu5Aca2-3Galb1-3GlcNAcb1-3Galb1-4GlcNAcb-Sp0	-8	-1
247	Fuca1-2(6S)Galb1-4Glcb-Sp0	24	22
248	Neu5Aca2-3Galb1-3GlcNAcb-Sp0	161	76
249	Neu5Aca2-3Galb1-4(6S)GlcNAcb-Sp8	6	9
250	Neu5Aca2-3Galb1-4(Fuca1-3)(6S)GlcNAcb-Sp8	1	1
251	Neu5Aca2-3Galb1-4(Fuca1-3)GlcNAcb1-3Galb1-4(Fuca1-3)GlcNAcb1-3Galb1-4(Fuca1-3)GlcNAcb-Sp0	123	77
252	Neu5Aca2-3Galb1-4(Fuca1-3)GlcNAcb-Sp0	7	3
253	Neu5Aca2-3Galb1-4(Fuca1-3)GlcNAcb-Sp8	-2	-4
254	Neu5Aca2-3Galb1-4(Fuca1-3)GlcNAcb1-3Galb-Sp8	31	7
255	Neu5Aca2-3Galb1-4(Fuca1-3)GlcNAcb1-3Galb1-4GlcNAcb-Sp8	53	36
256	Neu5Aca2-3Galb1-4GlcNAcb1-3Galb1-4GlcNAcb1-3Galb1-4GlcNAcb-Sp0	7	3
257	Neu5Aca2-3Galb1-4GlcNAcb-Sp0	45	33
258	Neu5Aca2-3Galb1-4GlcNAcb-Sp8	5	3
259	Neu5Aca2-3Galb1-4GlcNAcb1-3Galb1-4GlcNAcb-Sp0	-9	-9
260	Fuca1-2Galb1-4(6S)Glcb-Sp0	64	2
261	Neu5Aca2-3Galb1-4Glcb-Sp0	54	42
262	Neu5Aca2-3Galb1-4Glcb-Sp8	8	0
263	Neu5Aca2-6GalNAca-Sp8	4	5
264	Neu5Aca2-6GalNAcb1-4GlcNAcb-Sp0	1	-1
265	Neu5Aca2-6Galb1-4(6S)GlcNAcb-Sp8	2	1
266	Neu5Aca2-6Galb1-4GlcNAcb-Sp0	12	3
267	Neu5Aca2-6Galb1-4GlcNAcb-Sp8	2	8
268	Neu5Aca2-6Galb1-4GlcNAcb1-3Galb1-4(Fuca1-3)GlcNAcb1-3Galb1-4(Fuca1-3)GlcNAcb-Sp0	17	6
269	Neu5Aca2-6Galb1-4GlcNAcb1-3Galb1-4GlcNAcb-Sp0	-1	2
270	Neu5Aca2-6Galb1-4Glcb-Sp0	-4	1
271	Neu5Aca2-6Galb1-4Glcb-Sp8	6	3
272	Neu5Aca2-6Galb-Sp8	3	6
273	Neu5Aca2-8Neu5Aca-Sp8	5	0
274	Neu5Aca2-8Neu5Aca2-3Galb1-4Glcb-Sp0	7	0
275	Galb1-3(Fuca1-4)GlcNAcb1-3Galb1-3(Fuca1-4)GlcNAcb-Sp0	33	11
276	Neu5Acb2-6GalNAca-Sp8	-2	-1
277	Neu5Acb2-6Galb1-4GlcNAcb-Sp8	7	2
278	Neu5Gca2-3Galb1-3(Fuca1-4)GlcNAcb-Sp0	4	3
279	Neu5Gca2-3Galb1-3GlcNAcb-Sp0	2	3
280	Neu5Gca2-3Galb1-4(Fuca1-3)GlcNAcb-Sp0	2	6
281	Neu5Gca2-3Galb1-4GlcNAcb-Sp0	19	3
282	Neu5Gca2-3Galb1-4Glcb-Sp0	75	43
283	Neu5Gca2-6GalNAca-Sp0	16	10
284	Neu5Gca2-6Galb1-4GlcNAcb-Sp0	7	3
285	Neu5Gca-Sp8	24	10
286	Neu5Aca2-3Galb1-4GlcNAcb1-6(Galb1-3)GalNAca-Sp14	5	10
287	Galb1-3GlcNAcb1-3Galb1-3GlcNAcb-Sp0	9	6
288	Galb1-4(Fuca1-3)(6S)GlcNAcb-Sp0	7	6
289	Galb1-4(Fuca1-3)(6S)Glcb-Sp0	45	18
290	Galb1-4(Fuca1-3)GlcNAcb1-3Galb1-3(Fuca1-4)GlcNAcb-Sp0	28	21
291	Galb1-4GlcNAcb1-3Galb1-3GlcNAcb-Sp0	41	20
292	Neu5Aca2-3Galb1-3GlcNAcb1-3Galb1-3GlcNAcb-Sp0	10	5
293	Neu5Aca2-3Galb1-4GlcNAcb1-3Galb1-3GlcNAcb-Sp0	3	0
294	4S(3S)Galb1-4GlcNAcb-Sp0	101	27
295	(6S)Galb1-4(6S)GlcNAcb-Sp0	3	4
296	(6P)Glcb-Sp10	18	7
297	Neu5Aca2-3Galb1-4(Fuca1-3)GlcNAcb1-6(Galb1-3)GalNAca-Sp14	128	76
298	Galb1-3Galb1-4GlcNAcb-Sp8	8	9
299	Neu5Aca2-6Galb1-4GlcNAcb1-2Mana1-6(Galb1-4GlcNAcb1-2Mana1-3)Manb1-4GlcNAcb1-4GlcNAcb-Sp12	7	9
300	Galb1-4GlcNAcb1-6(Galb1-4GlcNAcb1-3)Galb1-4GlcNAc-Sp0	8	5
301	GlcNAcb1-6(Galb1-4GlcNAcb1-3)Galb1-4GlcNAc-Sp0	1	-1
302	Galb1-4GlcNAca1-6Galb1-4GlcNAcb-Sp0	4	4

303	Galb1-4GlcNAcb1-6Galb1-4GlcNAcb-Sp0	32	24
304	GalNAcb1-3Galb-Sp8	38	26
305	GlcAb1-3GlcNAcb-Sp8	17	7
306	Neu5Aca2-6Galb1-4GlcNAcb1-2Mana1-6(GlcNAcb1-2Mana1-3)Manb1-4GlcNAcb1-4GlcNAcb-Sp12	2	5
307	GlcNAcb1-3Man-Sp10	11	10
308	GlcNAcb1-4GlcNAcb-Sp10	-1	-1
309	GlcNAcb1-4GlcNAcb-Sp12	6	6
310	MurNAcb1-4GlcNAcb-Sp10	12	6
311	Mana1-6Manb-Sp10	1	1
312	Mana1-6(Mana1-3)Mana1-6(Mana1-3)Manb-Sp10	15	6
313	Mana1-2Mana1-6(Mana1-3)Mana1-6(Mana1-2Mana1-2Mana1-3)Mana-Sp9	2	3
314	Mana1-2Mana1-6(Mana1-2Mana1-3)Mana1-6(Mana1-2Mana1-2Mana1-3)Mana-Sp9	4	3
315	Neu5Aca2-3Galb1-4GlcNAcb1-6(Neu5Aca2-3Galb1-3)GalNAca-Sp14	1	3
316	Neu5Aca2-6Galb1-4GlcNAcb1-2Mana1-6(Neu5Aca2-3Galb1-4GlcNAcb1-2Mana1-3)Manb1-4GlcNAcb1-4GlcNAcb-Sp12	2	3
317	Galb1-4GlcNAcb1-2Mana1-6(Neu5Aca2-6Galb1-4GlcNAcb1-2Mana1-3)Manb1-4GlcNAcb1-4GlcNAcb-Sp12	1	3
318	Neu5Aca2-8Neu5Acb-Sp17	7	5
319	Neu5Aca2-8Neu5Aca2-8Neu5Acb-Sp8	1	0
320	Neu5Gcb2-6Galb1-4GlcNAc-Sp8	14	4
321	Galb1-3GlcNAcb1-2Mana1-6(Galb1-3GlcNAcb1-2Mana1-3)Manb1-4GlcNAcb1-4GlcNAcb-Sp19	50	28
322	Neu5Aca2-3Galb1-4GlcNAcb1-2Mana1-6(Neu5Aca2-3Galb1-4GlcNAcb1-2Mana1-3)Manb1-4GlcNAcb1-4GlcNAcb-Sp12	0	1
323	Neu5Aca2-3Galb1-4GlcNAcb1-2Mana1-6(Neu5Aca2-6Galb1-4GlcNAcb1-2Mana1-3)Manb1-4GlcNAcb1-4GlcNAcb-Sp12	-4	0
324	Galb1-4(Fuca1-3)GlcNAcb1-2Mana1-6(Galb1-4(Fuca1-3)GlcNAcb1-2Mana1-3)Manb1-4GlcNAcb1-4GlcNAcb-Sp20	23	10
325	Neu5,9Ac2a2-3Galb1-4GlcNAcb-Sp0	79	44
326	Neu5,9Ac2a2-3Galb1-3GlcNAcb-Sp0	2	0
327	Neu5Aca2-6Galb1-4GlcNAcb1-3Galb1-3GlcNAcb-Sp0	-29	-10
328	Neu5Aca2-3Galb1-3(Fuca1-4)GlcNAcb1-3Galb1-3(Fuca1-4)GlcNAcb-Sp0	14	9
329	Neu5Aca2-6Galb1-4GlcNAcb1-3Galb1-4GlcNAcb1-3Galb1-4GlcNAcb-Sp0	-8	-3
330	Gala1-4Galb1-4GlcNAcb1-3Galb1-4Glcb-Sp0	7	4
331	GalNAcb1-3Gala1-4Galb1-4GlcNAcb1-3Galb1-4Glcb-Sp0	23	8
332	GalNAca1-3(Fuca1-2)Galb1-4GlcNAcb1-3Galb1-4GlcNAcb-Sp0	-1	4
333	GalNAca1-3(Fuca1-2)Galb1-4GlcNAcb1-3Galb1-4GlcNAcb1-3Galb1-4GlcNAcb-Sp0	69	63
334	Neu5Aca2-3Galb1-4(Fuca1-3)GlcNAcb1-6(Neu5Aca2-3Galb1-3)GalNAc-Sp14	56	46
335	GlcNAca1-4Galb1-4GlcNAcb1-3Galb1-4GlcNAcb1-3Galb1-4GlcNAcb-Sp0	-4	-6
336	GlcNAca1-4Galb1-4GlcNAcb-Sp0	16	5
337	GlcNAca1-4Galb1-3GlcNAcb-Sp0	9	4
338	GlcNAca1-4Galb1-4GlcNAcb1-3Galb1-4Glcb-Sp0	2	3
339	GlcNAca1-4Galb1-4GlcNAcb1-3Galb1-4(Fuca1-3)GlcNAcb1-3Galb1-4(Fuca1-3)GlcNAcb-Sp0	67	33
340	GlcNAca1-4Galb1-4GlcNAcb1-3Galb1-4GlcNAcb-Sp0	17	6
341	GlcNAca1-4Galb1-3GalNAc-Sp14	20	4
342	Neu5Aca2-6Galb1-4GlcNAcb1-2Mana1-6(Mana1-3)Manb1-4GlcNAcb1-4GlcNAc-Sp12	3	5
343	Mana1-6(Neu5Aca2-6Galb1-4GlcNAcb1-2Mana1-3)Manb1-4GlcNAcb1-4GlcNAc-Sp12	7	9
344	Neu5Aca2-6Galb1-4GlcNAcb1-2Mana1-6Manb1-4GlcNAcb1-4GlcNAc-Sp12	4	4
345	Neu5Aca2-6Galb1-4GlcNAcb1-2Mana1-3Manb1-4GlcNAcb1-4GlcNAc-Sp12	9	17
346	Galb1-4GlcNAcb1-2Mana1-3Manb1-4GlcNAcb1-4GlcNAc-Sp12	-3	-4
347	Galb1-4GlcNAcb1-2Mana1-6Manb1-4GlcNAcb1-4GlcNAc-Sp12	7	8
348	Mana1-6(Galb1-4GlcNAcb1-2Mana1-3)Manb1-4GlcNAcb1-4GlcNAcb-Sp12	15	4

349	GlcNAcb1-2Mana1-6(GlcNAcb1-2Mana1-3)Manb1-4GlcNAcb1-4(Fuca1-6)GlcNAcb-Sp22	63	34
350	Galb1-4GlcNAcb1-2Mana1-6(Galb1-4GlcNAcb1-2Mana1-3)Manb1-4GlcNAcb1-4(Fuca1-6)GlcNAcb-Sp22	102	83
351	Galb1-3GlcNAcb1-2Mana1-6(Galb1-3GlcNAcb1-2Mana1-3)Manb1-4GlcNAcb1-4(Fuca1-6)GlcNAcb-Sp22	40	22
352	(6S)GlcNAcb1-3Galb1-4GlcNAcb-Sp0	57	35
353	KDNa2-3Galb1-4(Fuca1-3)GlcNAc-Sp0	26	18
354	KDNa2-6Galb1-4GlcNAc-Sp0	10	5
355	KDNa2-3Galb1-4Glc-Sp0	23	5
356	KDNa2-3Galb1-3GalNAca-Sp14	9	9
357	Fuca1-2Galb1-3GlcNAcb1-2Mana1-6(Fuca1-2Galb1-3GlcNAcb1-2Mana1-3)Manb1-4GlcNAcb1-4GlcNAcb-Sp20	153	123
358	Fuca1-2Galb1-4GlcNAcb1-2Mana1-6(Fuca1-2Galb1-4GlcNAcb1-2Mana1-3)Manb1-4GlcNAcb1-4GlcNAcb-Sp20	74	52
359	Fuca1-2Galb1-4(Fuca1-3)GlcNAcb1-2Mana1-6(Fuca1-2Galb1-4(Fuca1-3)GlcNAcb1-2Mana1-3)Manb1-4GlcNAcb1-4GlcNAb-Sp20	171	71
360	Gala1-3Galb1-4GlcNAcb1-2Mana1-6(Gala1-3Galb1-4GlcNAcb1-2Mana1-3)Manb1-4GlcNAcb1-4GlcNAcb-Sp20	21	15
361	Galb1-4GlcNAcb1-2Mana1-6(Mana1-3)Manb1-4GlcNAcb1-4GlcNAcb-Sp12	5	8
362	Fuca1-4(Galb1-3)GlcNAcb1-2Mana1-6(Fuca1-4(Galb1-3)GlcNAcb1-2Mana1-3)Manb1-4GlcNAcb1-4(Fuca1-6)GlcNAcb-Sp22	145	111
363	Neu5Aca2-6GlcNAcb1-4GlcNAc-Sp21	14	12
364	Neu5Aca2-6GlcNAcb1-4GlcNAcb1-4GlcNAc-Sp21	20	10
365	Galb1-4(Fuca1-3)GlcNAcb1-6(Fuca1-2Galb1-4GlcNAcb1-3)Galb1-4Glc-Sp21	27	15
366	Galb1-4GlcNAcb1-2Mana1-6(Galb1-4GlcNAcb1-4(Galb1-4GlcNAcb1-2)Mana1-3)Manb1-4GlcNAcb1-4GlcNAc-Sp21	39	22
367	GalNAca1-3(Fuca1-2)Galb1-4GlcNAcb1-2Mana1-6(GalNAca1-3(Fuca1-2)Galb1-4GlcNAcb1-2Mana1-3)Manb1-4GlcNAcb1-4GlcNAcb-Sp20	24	13
368	Gala1-3(Fuca1-2)Galb1-4GlcNAcb1-2Mana1-6(Gala1-3(Fuca1-2)Galb1-4GlcNAcb1-2Mana1-3)Manb1-4GlcNAcb1-4GlcNAcb-Sp20	27	16
369	Gala1-3Galb1-4(Fuca1-3)GlcNAcb1-2Mana1-6(Gala1-3Galb1-4(Fuca1-3)GlcNAcb1-2Mana1-3)Manb1-4GlcNAcb1-4GlcNAcb-Sp20	123	59
370	GalNAca1-3(Fuca1-2)Galb1-3GlcNAcb1-2Mana1-6(GalNAca1-3(Fuca1-2)Galb1-3GlcNAcb1-2Mana1-3)Manb1-4GlcNAcb1-4GlcNAcb-Sp20	87	69
371	Fuca1-4(Fuca1-2Galb1-3)GlcNAcb1-2Mana1-3(Fuca1-4(Fuca1-2Galb1-3)GlcNAcb1-2Mana1-3)Manb1-4GlcNAcb1-4GlcNAcb-Sp19	95	43
372	Neu5Aca2-3Galb1-4GlcNAcb1-3GalNAc-Sp14	3	5
373	Neu5Aca2-6Galb1-4GlcNAcb1-3GalNAc-Sp14	16	10
374	Neu5Aca2-3Galb1-4(Fuca1-3)GlcNAcb1-3GalNAca-Sp14	174	94
375	GalNAcb1-4GlcNAcb1-2Mana1-6(GalNAcb1-4GlcNAcb1-2Mana1-3)Manb1-4GlcNAcb1-4GlcNAc-Sp12	48	28
376	Galb1-3GalNAca1-3(Fuca1-2)Galb1-4Glc-Sp0	3	3
377	Galb1-3GalNAca1-3(Fuca1-2)Galb1-4GlcNAc-Sp0	6	3
378	Galb1-3GlcNAcb1-3Galb1-4GlcNAcb1-6(Galb1-3GlcNAcb1-3)Galb1-4Glcb-Sp0	24	10
379	Galb1-4(Fuca1-3)GlcNAcb1-6(Galb1-3GlcNAcb1-3)Galb1-4Glc-Sp21	38	25
380	Galb1-4GlcNAcb1-6(Fuca1-4(Fuca1-2Galb1-3)GlcNAcb1-3)Galb1-4Glc-Sp21	79	47
381	Galb1-4(Fuca1-3)GlcNAcb1-6(Fuca1-4(Fuca1-2Galb1-3)GlcNAcb1-3)Galb1-4Glc-Sp21	35	15
382	Galb1-3GlcNAcb1-3Galb1-4(Fuca1-3)GlcNAcb1-6(Galb1-3GlcNAcb1-3)Galb1-4Glc-Sp21	25	15
383	Galb1-4GlcNAcb1-6(Galb1-4GlcNAcb1-2)Mana1-6(Galb1-4GlcNAcb1-4(Galb1-4GlcNAcb1-2)Mana1-3)Manb1-4GlcNAcb1-4GlcNAcb-Sp21	34	15
384	GlcNAcb1-2Mana1-6(GlcNAcb1-4(GlcNAcb1-2)Mana1-3)Manb1-4GlcNAcb1-4GlcNAc-Sp21	35	24
385	Fuca1-2Galb1-3GalNAca1-3(Fuca1-2)Galb1-4Glcb-Sp0	29	11
386	Fuca1-2Galb1-3GalNAca1-3(Fuca1-2)Galb1-4GlcNAcb-Sp0	21	11
387	Galb1-3GlcNAcb1-3GalNAca-Sp14	23	11
388	GalNAcb1-4(Neu5Aca2-3)Galb1-4GlcNAcb1-3GalNAca-Sp14	172	66
389	GalNAca1-3(Fuca1-2)Galb1-3GalNAca1-3(Fuca1-2)Galb1-4GlcNAcb-Sp0	9	1
390	Gala1-3Galb1-3GlcNAcb1-2Mana1-6(Gala1-3Galb1-3GlcNAcb1-2Mana1-3)Manb1-4GlcNAcb1-4GlcNAc-Sp19	83	63

391	Gala1-3Galb1-3(Fuca1-4)GlcNAcb1-2Mana1-6(Gala1-3Galb1-3(Fuca1-4)GlcNAcb1-2Mana1-3)Manb1-4GlcNAcb1-4GlcNAc-Sp19	92	88
392	Neu5Aca2-3Galb1-3GlcNAcb1-2Mana1-6(Neu5Aca2-3Galb1-3GlcNAcb1-2Mana1-3)Manb1-4GlcNAcb1-4GlcNAc-Sp19	22	6
393	GlcNAcb1-2Mana1-6(Galb1-4GlcNAcb1-2Mana1-3)Manb1-4GlcNAcb1-4GlcNAc-Sp12	23	5
394	Galb1-4GlcNAcb1-2Mana1-6(GlcNAcb1-2Mana1-3)Manb1-4GlcNAcb1-4GlcNAc-Sp12	2	3
395	Neu5Aca2-3Galb1-3GlcNAcb1-3GalNAca-Sp14	36	21
396	Fuca1-2Galb1-4GlcNAcb1-3GalNAca-Sp14	42	32
397	Galb1-4(Fuca1-3)GlcNAcb1-3GalNAca-Sp14	50	37
398	GalNAca1-3GalNAcb1-3Gala1-4Galb1-4GlcNAcb-Sp0	-2	0
399	Gala1-4Galb1-3GlcNAcb1-2Mana1-6(Gala1-4Galb1-3GlcNAcb1-2Mana1-3)Manb1-4GlcNAcb1-4GlcNAcb-Sp19	46	56
400	Gala1-4Galb1-4GlcNAcb1-2Mana1-6(Gala1-4Galb1-4GlcNAcb1-2Mana1-3)Manb1-4GlcNAcb1-4GlcNAcb-Sp24	143	119
401	Gala1-3Galb1-4GlcNAcb1-3GalNAca-Sp14	-2	0
402	Galb1-3GlcNAcb1-6Galb1-4GlcNAcb-Sp0	2	2
403	Galb1-3GlcNAca1-6Galb1-4GlcNAcb-Sp0	6	-2
404	GalNAcb1-3Gala1-6Galb1-4Glcb-Sp8	15	5
405	Gala1-3(Fuca1-2)Galb1-4(Fuca1-3)Glcb-Sp21	44	13
406	Galb1-4GlcNAcb1-6(Neu5Aca2-6Galb1-3GlcNAcb1-3)Galb1-4Glc-Sp21	28	16
407	Galb1-3GalNAcb1-4(Neu5Aca2-8Neu5Aca2-3)Galb1-4Glcb-Sp0	26	12
408	Neu5Aca2-3Galb1-3GalNAcb1-4(Neu5Aca2-8Neu5Aca2-3)Galb1-4Glcb-Sp0	27	20
409	Gala1-3(Fuca1-2)Galb1-4GlcNAcb1-3GalNAca-Sp14	7	2
410	GalNAca1-3(Fuca1-2)Galb1-4GlcNAcb1-3GalNAca-Sp14	18	14
411	GalNAca1-3GalNAcb1-3Gala1-4Galb1-4Glcb-Sp0	6	10
412	Fuca1-2Galb1-4(Fuca1-3)GlcNAcb1-3GalNAca-Sp14	227	168
413	Gala1-3(Fuca1-2)Galb1-4(Fuca1-3)GlcNAcb1-3GalNAc-Sp14	50	51
414	GalNAca1-3(Fuca1-2)Galb1-4(Fuca1-3)GlcNAcb1-3GalNAc-Sp14	155	106
415	Galb1-4(Fuca1-3)GlcNAcb1-2Mana1-6(Galb1-4(Fuca1-3)GlcNAcb1-2Mana1-3)Manb1-4GlcNAcb1-4(Fuca1-6)GlcNAcb-Sp22	57	26
416	Fuca1-2Galb1-4GlcNAcb1-2Mana1-6(Fuca1-2Galb1-4GlcNAcb1-2Mana1-3)Manb1-4GlcNAcb1-4(Fuca1-6)GlcNAcb-Sp22	142	123
417	GlcNAcb1-2(GlcNAcb1-6)Mana1-6(GlcNAcb1-2Mana1-3)Manb1-4GlcNAcb1-4GlcNAcb-Sp19	19	9
418	Fuca1-2Galb1-3GlcNAcb1-3GalNAc-Sp14	8	9
419	Gala1-3(Fuca1-2)Galb1-3GlcNAcb1-3GalNAc-Sp14	15	8
420	GalNAca1-3(Fuca1-2)Galb1-3GlcNAcb1-3GalNAc-Sp14	92	58
421	Gala1-3Galb1-3GlcNAcb1-3GalNAc-Sp14	4	8
422	Fuca1-2Galb1-3GlcNAcb1-2Mana1-6(Fuca1-2Galb1-3GlcNAcb1-2Mana1-3)Manb1-4GlcNAcb1-4(Fuca1-6)GlcNAcb-Sp22	150	94
423	Gala1-3(Fuca1-2)Galb1-4GlcNAcb1-2Mana1-6(Gala1-3(Fuca1-2)Galb1-4GlcNAcb1-2Mana1-3)Manb1-4GlcNAcb1-4(Fuca1-6)GlcNAcb-Sp22	200	175
424	Galb1-3GlcNAcb1-6(Galb1-3GlcNAcb1-2)Mana1-6(Galb1-3GlcNAcb1-2Mana1-3)Manb1-4GlcNAcb1-4GlcNAcb-Sp19	43	28
425	Galb1-4GlcNAcb1-6(Fuca1-2Galb1-3GlcNAcb1-3)Galb1-4Glc-Sp21	30	18
426	Fuca1-3GlcNAcb1-6(Galb1-4GlcNAcb1-3)Galb1-4Glc-Sp21	43	19
427	GlcNAcb1-2Mana1-6(GlcNAcb1-4)(GlcNAcb1-2Mana1-3)Manb1-4GlcNAcb1-4GlcNAc-Sp21	81	18
428	GlcNAcb1-2Mana1-6(GlcNAcb1-4)(GlcNAcb1-4(GlcNAcb1-2)Mana1-3)Manb1-4GlcNAcb1-4GlcNAc-Sp21	23	10
429	GlcNAcb1-6(GlcNAcb1-2)Mana1-6(GlcNAcb1-4)(GlcNAcb1-2Mana1-3)Manb1-4GlcNAcb1-4GlcNAc-Sp21	26	12
430	GlcNAcb1-6(GlcNAcb1-2)Mana1-6(GlcNAcb1-4)(GlcNAcb1-4(GlcNAcb1-2)Mana1-3)Manb1-4GlcNAcb1-4GlcNAc-Sp21	31	25
431	Galb1-4GlcNAcb1-2Mana1-6(GlcNAcb1-4)(Galb1-4GlcNAcb1-2Mana1-3)Manb1-4GlcNAcb1-4GlcNAc-Sp21	48	20
432	Galb1-4GlcNAcb1-2Mana1-6(GlcNAcb1-4)(Galb1-4GlcNAcb1-4(Galb1-4GlcNAcb1-2)Mana1-3)Manb1-4GlcNAcb1-4GlcNAc-Sp21	31	22
433	Galb1-4GlcNAcb1-6(Galb1-4GlcNAcb1-2)Mana1-6(GlcNAcb1-4)(Galb1-4GlcNAcb1-2Mana1-3)Manb1-4GlcNAcb1-4GlcNAc-Sp21	36	22
434	Galb1-4GlcNAcb1-6(Galb1-4GlcNAcb1-2)Mana1-6(GlcNAcb1-4)(Galb1-4GlcNAcb1-4(Galb1-4GlcNAcb1-2)Mana1-3)Manb1-4GlcNAcb1-4GlcNAc-Sp21	26	15
435	Galb1-4Galb-Sp10	30	12

436	Galb1-6Galb-Sp10	30	10
437	Neu5Aca2-3Galb1-4GlcNAcb1-3Galb-Sp8	10	4
438	GalNAcb1-6GalNAcb-Sp8	12	4
439	(6S)Galb1-3GlcNAcb-Sp0	11	5
440	(6S)Galb1-3(6S)GlcNAc-Sp0	18	13
441	Fuca1-2Galb1-4 GlcNAcb1-2Mana1-6(Fuca1-2Galb1-4GlcNAcb1-2(Fuca1-2Galb1-4GlcNAcb1-4)Mana1-3)Manb1-4GlcNAcb1-4GlcNAcb-Sp12	86	73
442	Fuca1-2Galb1-4(Fuca1-3)GlcNAcb1-2Mana1-6(Fuca1-2Galb1-4(Fuca1-3)GlcNAcb1-4(Fuca1-2Galb1-4(Fuca1-3)GlcNAcb1-2)Mana1-3)Manb1-4GlcNAcb1-4GlcNAcb-Sp12	140	92
443	Galb1-4(Fuca1-3)GlcNAcb1-6GalNAc-Sp14	104	68
444	Galb1-4GlcNAcb1-2Mana-Sp0	42	28
445	Fuca1-2Galb1-4GlcNAcb1-6(Fuca1-2Galb1-4GlcNAcb1-3)GalNAc-Sp14	26	16
446	Gala1-3(Fuca1-2)Galb1-4GlcNAcb1-6(Gala1-3(Fuca1-2)Galb1-4GlcNAcb1-3)GalNAc-Sp14	3	8
447	GalNAca1-3(Fuca1-2)Galb1-4GlcNAcb1-6(GalNAca1-3(Fuca1-2)Galb1-4GlcNAcb1-3)GalNAc-Sp14	12	2
448	Neu5Aca2-8Neu5Aca2-3Galb1-3GalNAcb1-4(Neu5Aca2-8Neu5Aca2-3)Galb1-4Glcb-Sp0	16	9
449	GalNAcb1-4Galb1-4Glcb-Sp0	54	15
450	GalNAca1-3(Fuca1-2)Galb1-4GlcNAcb1-2Mana1-6(GalNAca1-3(Fuca1-2)Galb1-4GlcNAcb1-2Mana1-3)Manb1-4GlcNAcb1-4(Fuca1-6)GlcNAcb-Sp22	42	24
451	Gala1-3(Fuca1-2)Galb1-3GlcNAcb1-2Mana1-6(Gala1-3(Fuca1-2)Galb1-3GlcNAcb1-2Mana1-3)Manb1-4GlcNAcb1-4(Fuca1-6)GlcNAcb-Sp22	210	179
452	Neu5Aca2-6Galb1-4GlcNAcb1-6(Fuca1-2Galb1-3GlcNAcb1-3)Galb1-4Glc-Sp21	26	24
453	GalNAca1-3(Fuca1-2)Galb1-3GlcNAcb1-2Mana1-6(GalNAca1-3(Fuca1-2)Galb1-3GlcNAcb1-2Mana1-3)Manb1-4GlcNAcb1-4(Fuca1-6)GlcNAcb-Sp22	411	260
454	Galb1-4GlcNAcb1-6(Galb1-4GlcNAcb1-2)Mana1-6(Galb1-4GlcNAcb1-2Mana1-3)Manb1-4GlcNAcb1-4GlcNAcb-Sp19	55	35
455	Neu5Aca2-3Galb1-4GlcNAcb1-2Mana1-6(GlcNAcb1-4)(Neu5Aca2-3Galb1-4GlcNAcb1-2Mana1-3)Manb1-4GlcNAcb1-4GlcNAcb-Sp21	22	11
456	Neu5Aca2-3Galb1-4GlcNAcb1-4Mana1-6(GlcNAcb1-4)(Neu5Aca2-3Galb1-4GlcNAcb1-4(Neu5Aca2-3Galb1-4GlcNAcb1-2)Mana1-3)Manb1-4GlcNAcb1-4GlcNAcb-Sp21	12	10
457	Neu5Aca2-3Galb1-4GlcNAcb1-6(Neu5Aca2-3Galb1-4GlcNAcb1-2)Mana1-6(GlcNAcb1-4)(Neu5Aca2-3Galb1-4GlcNAcb1-2Mana1-3)Manb1-4GlcNAcb1-4GlcNAcb-Sp21	16	7
458	Neu5Aca2-3Galb1-4GlcNAcb1-6(Neu5Aca2-3Galb1-4GlcNAcb1-2)Mana1-6(GlcNAcb1-4)(Neu5Aca2-3Galb1-4GlcNAcb1-4(Neu5Aca2-3Galb1-4GlcNAcb1-2)Mana1-3)Manb1-4GlcNAcb1-4GlcNAcb-Sp21	9	7
459	Neu5Aca2-6Galb1-4GlcNAcb1-2Mana1-6(GlcNAcb1-4)(Neu5Aca2-6Galb1-4GlcNAcb1-2Mana1-3)Manb1-4GlcNAcb1-4GlcNAcb-Sp21	41	25
460	Neu5Aca2-6Galb1-4GlcNAcb1-4Mana1-6(GlcNAcb1-4)(Neu5Aca2-6Galb1-4GlcNAcb1-4(Neu5Aca2-6Galb1-4GlcNAcb1-2)Mana1-3)Manb1-4GlcNAcb1-4GlcNAcb-Sp21	16	12
461	Neu5Aca2-6Galb1-4GlcNAcb1-6(Neu5Aca2-6Galb1-4GlcNAcb1-2)Mana1-6(GlcNAcb1-4)(Neu5Aca2-6Galb1-4GlcNAcb1-2Mana1-3)Manb1-4GlcNAcb1-4GlcNAcb-Sp21	20	17
462	Neu5Aca2-6Galb1-4GlcNAcb1-6(Neu5Aca2-6Galb1-4GlcNAcb1-2)Mana1-6(GlcNAcb1-4)(Neu5Aca2-6Galb1-4GlcNAcb1-4(Neu5Aca2-6Galb1-4GlcNAcb1-2)Mana1-3)Manb1-4GlcNAcb1-4GlcNAcb-Sp21	14	9
463	Gala1-3(Fuca1-2)Galb1-3GalNAca-Sp8	13	9
464	Gala1-3(Fuca1-2)Galb1-3GalNAcb-Sp8	11	2
465	Glca1-6Glca1-6Glca1-6Glcb-Sp10	41	14
466	Glca1-4Glca1-4Glca1-4Glcb-Sp10	15	19
467	Neu5Aca2-3Galb1-4GlcNAcb1-6(Neu5Aca2-3Galb1-4GlcNAcb1-3)GalNAca-Sp14	-2	-4
468	Fuca1-2Galb1-4(Fuca1-3)GlcNAcb1-2Mana1-6(Fuca1-2Galb1-4(Fuca1-3)GlcNAcb1-2Mana1-3)Manb1-4GlcNAcb1-4(Fuca1-6)GlcNAcb-Sp24	3	24
469	Fuca1-2Galb1-3(Fuca1-4)GlcNAcb1-2Mana1-6(Fuca1-2Galb1-3(Fuca1-4)GlcNAcb1-2Mana1-3)Manb1-4GlcNAcb1-4(Fuca1-6)GlcNAcb1-4(Fuca1-6)GlcNAcb-Sp19	127	85

470	GlcNAcb1-6(GlcNAcb1-2)Mana1-6(GlcNAcb1-2Mana1-3)Manb1-4GlcNAcb1-4(Fuca1-6)GlcNAcb-Sp24	177	128
471	Galb1-3GlcNAcb1-2Mana1-6(GlcNAcb1-4)(Galb1-3GlcNAcb1-2Mana1-3)Manb1-4GlcNAcb1-4GlcNAcb-Sp21	94	69
472	Neu5Aca2-6Galb1-4GlcNAcb1-6(Galb1-3GlcNAcb1-3)Galb1-4Glcb-Sp21	31	22
473	Neu5Aca2-3Galb1-4GlcNAcb1-2Mana-Sp0	78	49
474	Neu5Aca2-3Galb1-4GlcNAcb1-6GalNAca-Sp14	5	3
475	Neu5Aca2-6Galb1-4GlcNAcb1-6GalNAca-Sp14	54	10
476	Neu5Aca2-6Galb1-4 GlcNAcb1-6(Neu5Aca2-6Galb1-4GlcNAcb1-3)GalNAca-Sp14	6	4
477	Neu5Aca2-6Galb1-4GlcNAcb1-2Mana1-6(Neu5Aca2-6Galb1-4GlcNAcb1-2Mana1-3)Manb1-4GlcNAcb1-4(Fuca1-6)GlcNAcb-Sp24	131	60
478	Neu5Aca2-3Galb1-4GlcNAcb1-2Mana1-6(Neu5Aca2-3Galb1-4GlcNAcb1-2Mana1-3)Manb1-4GlcNAcb1-4(Fuca1-6)GlcNAcb-Sp24	73	45
479	Mana1-6(Mana1-3)Manb1-4GlcNAcb1-4(Fuca1-6)GlcNAcb-Sp19	29	12
480	Galb1-4GlcNAcb1-6(Galb1-4GlcNAcb1-2)Mana1-6(Galb1-4GlcNAcb1-2Mana1-3)Manb1-4GlcNAcb1-4(Fuca1-6)GlcNAcb-Sp24	128	94
481	Neu5Aca2-3Galb1-3GlcNAcb1-2Mana1-6(GlcNAcb1-4)(Neu5Aca2-3Galb1-3GlcNAcb1-2Mana1-3)Manb1-4GlcNAcb1-4GlcNAc-Sp21	50	10
482	Neu5Aca2-6Galb1-4GlcNAcb1-6(Fuca1-2Galb1-4(Fuca1-3)GlcNAcb1-3)Galb1-4Glc-Sp21	53	46
483	Galb1-3GlcNAcb1-6GalNAca-Sp14	1	2
484	Gala1-3Galb1-3GlcNAcb1-6GalNAca-Sp14	9	3
485	Galb1-3(Fuca1-4)GlcNAcb1-6GalNAca-Sp14	86	37
486	Neu5Aca2-3Galb1-3GlcNAcb1-6GalNAca-Sp14	4	6
487	(3S)Galb1-3(Fuca1-4)GlcNAcb-Sp0	44	15
488	Galb1-4(Fuca1-3)GlcNAcb1-6(Neu5Aca2-6(Neu5Aca2-3Galb1-3)GlcNAcb1-3)Galb1-4Glc-Sp21	50	30
489	Fuca1-2Galb1-4GlcNAcb1-6GalNAca-Sp14	75	-1
490	Gala1-3Galb1-4GlcNAcb1-6GalNAca-Sp14	11	3
491	Galb1-4(Fuca1-3)GlcNAcb1-2Mana-Sp0	35	12
492	Fuca1-2(6S)Galb1-3GlcNAcb-Sp0	10	0
493	Gala1-3(Fuca1-2)Galb1-4GlcNAcb1-6GalNAca-Sp14	29	12
494	Fuca1-2Galb1-4GlcNAcb1-2Mana-Sp0	46	12
495	Fuca1-2Galb1-3(6S)GlcNAcb-Sp0	41	22
496	Fuca1-2(6S)Galb1-3(6S)GlcNAcb-Sp0	85	51
497	Neu5Aca2-6GalNAcb1-4(6S)GlcNAcb-Sp8	10	3
498	GalNAcb1-4(Fuca1-3)(6S)GlcNAcb-Sp8	6	7
499	(3S)GalNAcb1-4(Fuca1-3)GlcNAcb-Sp8	14	8
500	Fuca1-2Galb1-3GlcNAcb1-6(Fuca1-2Galb1-3GlcNAcb1-3)GalNAca-Sp14	20	10
501	GalNAca1-3(Fuca1-2)Galb1-3GlcNAcb1-6GalNAca-Sp14	-1	-1
502	GlcNAcb1-6(GlcNAcb1-2)Mana1-6(GlcNAcb1-4)(GlcNAcb1-4(GlcNAcb1-2)Mana1-3)Manb1-4GlcNAcb1-4(Fuca1-6)GlcNAc-Sp21	15	10
503	Galb1-4GlcNAcb1-6(Galb1-4GlcNAcb1-2)Mana1-6(GlcNAcb1-4)Galb1-4GlcNAcb1-4(Gal b1-4GlcNAcb1-2)Mana1-3)Manb1-4GlcNAcb1-4(Fuca1-6)GlcNAc-Sp21	27	14
504	Galb1-3GlcNAca1-3Galb1-4GlcNAcb-Sp8	8	4
505	Galb1-3(6S)GlcNAcb-Sp8	2	-4
506	(6S)(4S)GalNAcb1-4GlcNAc-Sp8	2	5
507	(6S)GalNAcb1-4GlcNAc-Sp8	-3	-2
508	(3S)GalNAcb1-4(3S)GlcNAc-Sp8	7	4
509	GalNAcb1-4(6S)GlcNAc-Sp8	3	5
510	(3S)GalNAcb1-4GlcNAc-Sp8	11	4
511	(4S)GalNAcb-Sp10	4	-1
512	Galb1-4(6P)GlcNAcb-Sp0	14	6
513	(6P)Galb1-4GlcNAcb-SP0	18	10
514	GalNAca1-3(Fuca1-2)Galb1-4GlcNAcb1-6GalNAc-Sp14	2	0
515	Neu5Aca2-6Galb1-4GlcNAcb1-2Man-Sp0	9	9
516	Gala1-3Galb1-4GlcNAcb1-2Mana-Sp0	5	1
517	Gala1-3(Fuca1-2)Galb1-4GlcNAcb1-2Mana-Sp0	15	6
518	GalNAca1-3(Fuca1-2)Galb1-4 GlcNAcb1-2Mana-Sp0	6	5
519	Galb1-3GlcNAcb1-2Mana-Sp0	20	10
520	Gala1-3(Fuca1-2)Galb1-3GlcNAcb1-6GalNAc-Sp14	1	1
521	Neu5Aca2-3Galb1-3GlcNAcb1-2Mana-Sp0	7	6
522	Gala1-3Galb1-3GlcNAcb1-2Mana-Sp0	10	3

523	GalNAcb1-4GlcNAcb1-2Mana-Sp0	11	5
524	Neu5Aca2-3Galb1-3GalNAcb1-4Galb1-4Glcb-Sp0	22	12
525	GlcNAcb1-2 Mana1-6(GlcNAcb1-4)(GlcNAcb1-2Mana1-3)Manb1-4GlcNAcb1-4(Fuca1-6)GlcNAc-Sp21	13	8
526	Galb1-4GlcNAcb1-2 Mana1-6(GlcNAcb1-4)(Galb1-4GlcNAcb1-2Mana1-3)Manb1-4GlcNAcb1-4(Fuca1-6)GlcNAc-Sp21	19	12
527	Galb1-4GlcNAcb1-2 Mana1-6(Galb1-4GlcNAcb1-4)(Galb1-4GlcNAcb1-2Mana1-3)Manb1-4GlcNAcb1-4(Fuca1-6)GlcNAc-Sp21	22	22
528	Fuca1-4(Galb1-3)GlcNAcb1-2 Mana-Sp0	105	79
529	Neu5Aca2-3Galb1-4(Fuca1-3)GlcNAcb1-2Mana-Sp0	2	5
530	GlcNAcb1-3Galb1-4GlcNAcb1-6(GlcNAcb1-3)Galb1-4GlcNAc-Sp0	3	6
531	GalNAca1-3(Fuca1-2)Galb1-3GalNAcb1-3Gala1-4Galb1-4Glc-Sp21	14	7
532	Gala1-3(Fuca1-2)Galb1-3GalNAcb1-3Gala1-4Galb1-4Glc-Sp21	44	24
533	Galb1-3GalNAcb1-3Gal-Sp21	30	13
534	GlcNAcb1-3Galb1-4GlcNAcb1-2Mana1-6(GlcNAcb1-3Galb1-4GlcNAcb1-2Mana1-3)Manb1-4GlcNAcb1-4GlcNAcb-Sp12	7	7
535	GlcNAcb1-3Galb1-4GlcNAcb1-2Mana1-6(GlcNAcb1-3Galb1-4GlcNAcb1-2Mana1-3)Manb1-4GlcNAcb1-4GlcNAcb-Sp25	172	98
536	Fuca1-2Galb1-4GlcNAcb1-3Galb1-4GlcNAcb1-2Mana1-6(Fuca1-2Galb1-4GlcNAcb1-3Galb1-4GlcNAcb1-2Mana1-3)Manb1-4GlcNAcb1-4GlcNAcb-Sp24	153	93
537	GlcNAcb1-3Galb1-4GlcNAcb1-3Galb1-4GlcNAcb1-2Mana1-6(GlcNAcb1-3Galb1-4GlcNAcb1-3Galb1-4GlcNAcb1-2Mana1-3)Manb1-4GlcNAcb1-4GlcNAcb-Sp12	798	382
538	GlcNAcb1-3Galb1-4GlcNAcb1-3Galb1-4GlcNAcb1-2Mana1-6(GlcNAcb1-3Galb1-4GlcNAcb1-3Galb1-4GlcNAcb1-2Mana1-3)Manb1-4GlcNAcb1-4GlcNAcb-Sp25	155	124
539	Galb1-4GlcNAcb1-3Galb1-4GlcNAcb1-3Galb1-4GlcNAcb1-2Mana1-6(Galb1-4GlcNAcb1-3Galb1-4GlcNAcb1-3Galb1-4GlcNAcb1-2Mana1-3)Manb1-4GlcNAcb1-4GlcNAcb-Sp12	1341	968
540	Galb1-4GlcNAcb1-3Galb1-4GlcNAcb1-3Galb1-4GlcNAcb1-2Mana1-6(Galb1-4GlcNAcb1-3Galb1-4GlcNAcb1-3Galb1-4GlcNAcb1-2Mana1-3)Manb1-4GlcNAcb1-4GlcNAcb-Sp24	152	102
541	Galb1-3GlcNAcb1-3Galb1-4GlcNAcb1-2Mana1-6(Galb1-3GlcNAcb1-3Galb1-4GlcNAcb1-2Mana1-3)Manb1-4GlcNAcb1-4GlcNAc-Sp25	82	60
542	Neu5Gca2-8Neu5Gca2-3Galb1-4GlcNAc-Sp0	9	4
543	Neu5Aca2-8Neu5Gca2-3Galb1-4GlcNAc-Sp0	-5	-5
544	Neu5Gca2-8Neu5Aca2-3Galb1-4GlcNAc-Sp0	-3	-2
545	Neu5Gca2-8Neu5Gca2-3Galb1-4GlcNAcb1-3Galb1-4GlcNAc-Sp0	-5	1
546	Neu5Gca2-8Neu5Gca2-6Galb1-4GlcNAc-Sp0	-1	0
547	Neu5Aca2-8Neu5Aca2-3Galb1-4GlcNAc-Sp0	-1	0
548	GlcNAcb1-3Galb1-4GlcNAcb1-6(GlcNAcb1-3Galb1-4GlcNAcb1-2)Mana1-6(GlcNAcb1-3Galb1-4GlcNAcb1-2Man a1-3)Manb1-4GlcNAcb1-4GlcNAc-Sp24	217	149
549	Galb1-4GlcNAcb1-3Galb1-4GlcNAcb1-6(Galb1-4GlcNAcb1-3Galb1-4GlcNAcb1-2)Mana1-6(Galb1-4GlcNAcb1-3Galb1-4GlcNAcb1-2Mana1-3)Mana1-4GlcNAcb1-4GlcNAc-Sp24	121	69
550	Gala1-3Galb1-4GlcNAcb1-2Mana1-6(Gala1-3Galb1-4GlcNAcb1-2Mana1-3)Manb1-4GlcNAcb1-4GlcNAc-Sp24	176	175
551	GlcNAcb1-3Galb1-4GlcNAcb1-6(GlcNAcb1-3Galb1-3)GalNAca-Sp14	-1	-2
552	GalNAcb1-3GlcNAcb-Sp0	8	3
553	GalNAcb1-4GlcNAcb1-3GalNAcb1-4GlcNAcb-Sp0	-9	-6
554	GlcNAcb1-3Galb1-4GlcNAcb1-3Galb1-4GlcNAcb1-3Galb1-4GlcNAcb1-3Galb1-4GlcNAcb1-2Mana1-6(GlcNAcb1-3Galb1-4GlcNAcb1-3Galb1-4GlcNAcb1-3Galb1-4GlcNAcb1-3Galb1-4GlcNAcb1-2Mana1-3)Manb1-4GlcNAcb1-4GlcNAcb-Sp25	48	68
555	Galb1-4GlcNAcb1-3Galb1-4GlcNAcb1-3Galb1-4GlcNAcb1-3Galb1-4GlcNAcb1-3Galb1-4GlcNAcb1-2Mana1-6(Galb1-4GlcNAcb1-3Galb1-4GlcNAcb1-3Galb1-4GlcNAcb1-3Galb1-4GlcNAcb1-3Galb1-4GlcNAcb1-2Mana1-3)Manb1-4GlcNAcb1-4GlcNAcb-Sp25	72	57
556	GlcNAb1-3Galb1-3GalNAc-Sp14	-1	-3
557	Galb1-3GlcNAcb1-6(Galb1-3)GalNAc-Sp14	3	1
558	Galb1-4GlcNAcb1-3Galb1-4GlcNAcb1-3Galb1-4GlcNAcb1-3Galb1-4GlcNAcb1-3Galb1-4GlcNAcb1-3Galb1-4GlcNAcb1-2Mana1-6(Galb1-4GlcNAcb1-3Galb1-4GlcNAcb1-3Galb1-4GlcNAcb1-3Galb1-4GlcNAcb1-3Galb1-4GlcNAcb1-3Galb1-4GlcNAcb1-2Mana1-3)Manb1-4GlcNAcb1-4GlcNAcb-Sp25	-1	-1

559	(3S)GlcAb1-3Galb1-4GlcNAcb1-3Galb1-4Glc-Sp0	-1	-1
560	(3S)GlcAb1-3Galb1-4GlcNAcb1-2Mana-Sp0	0	-2
561	Galb1-3GlcNAcb1-3Galb1-4GlcNAcb1-3Galb1-4GlcNAcb1-6(Galb1-3GlcNAcb1-3Galb1-4GlcNAcb1-3Galb1-4GlcNAb1-2)Mana1-6(Galb1-3GlcNAcb1-3Galb1-4GlcNAcb1-3Galb1-4GlcNAcb1-2Mana1-3)Manb1-4GlcNAcb1-4(Fuca1-6)GlcNAcb-Sp24	79	52
562	Galb1-3GlcNAcb1-3Galb1-4GlcNAcb1-6(Galb1-3GlcNAcb1-3Galb1-4GlcNAb1-2)Mana1-6(Galb1-3GlcNAcb1-3Galb1-4GlcNAcb1-2Mana1-3)Manb1-4GlcNAcb1-4(Fuca1-6)GlcNAcb-Sp24	196	90
563	Neu5Aca2-8Neu5Aca2-3Galb1-3GalNAcb1-4(Neu5Aca2-3)Galb1-4Glc-Sp21	25	19
564	GlcNAcb1-3Galb1-4GlcNAcb1-2Mana1-6(GlcNAcb1-3Galb1-4GlcNAcb1-2Mana1-3)Manb1-4GlcNAcb1-4(Fuca1-6)GlcNAcb-Sp24	178	87
565	Galb1-4GlcNAcb1-3Galb1-4GlcNAcb1-2Mana1-6(Galb1-4GlcNAcb1-3Galb1-4GlcNAcb1-2Mana1-3)Manb1-4GlcNAcb1-4(Fuca1-6)GlcNAcb-Sp24	145	113
566	GlcNAcb1-3Galb1-4GlcNAcb1-3Galb1-4GlcNAcb1-2Mana1-6(GlcNAcb1-3Galb1-4GlcNAcb1-3Galb1-4GlcNAcb1-2Mana1-3)Manb1-4GlcNAcb1-4(Fuca1-6)GlcNAcb-Sp24	12	7
567	Galb1-4GlcNAcb1-3Galb1-4GlcNAcb1-3Galb1-4GlcNAcb1-2Mana1-6(Galb1-4GlcNAcb1-3Galb1-4GlcNAcb1-3Galb1-4GlcNAcb1-2Mana1-3)Manb1-4GlcNAcb1-4(Fuca1-6)GlcNAcb-Sp24	31	42
568	GlcNAcb1-3Galb1-4GlcNAcb1-3Galb1-4GlcNAcb1-3Galb1-4GlcNAcb1-2Mana1-6(GlcNAcb1-3Galb1-4GlcNAcb1-3Galb1-4GlcNAcb1-3Galb1-4GlcNAcb1-2Mana1-3)Manb1-4GlcNAcb1-4(Fuca1-6)GlcNAcb-Sp24	44	68
569	Galb1-4GlcNAcb1-3Galb1-4GlcNAcb1-3Galb1-4GlcNAcb1-3Galb1-4GlcNAcb1-2Mana1-6(Galb1-4GlcNAcb1-3Galb1-4GlcNAcb1-3Galb1-4GlcNAcb1-3Galb1-4GlcNAcb1-2Mana1-3)Manb1-4GlcNAcb1-4(Fuca1-6)GlcNAcb-Sp24	44	94
570	GlcNAcb1-3Galb1-4GlcNAcb1-3Galb1-4GlcNAcb1-3Galb1-4GlcNAcb1-3Galb1-4GlcNAcb1-2Mana1-6(GlcNAcb1-3Galb1-4GlcNAcb1-3Galb1-4GlcNAcb1-3Galb1-4GlcNAcb1-3Galb1-4GlcNAcb1-2Mana1-3)Manb1-4GlcNAcb1-4(Fuca1-6)GlcNAcb-Sp19	12	18
571	Galb1-4GlcNAcb1-3Galb1-4GlcNAcb1-3Galb1-4GlcNAcb1-3Galb1-4GlcNAcb1-3Galb1-4GlcNAcb1-2Mana1-6(Galb1-4GlcNAcb1-3Galb1-4GlcNAcb1-3Galb1-4GlcNAcb1-3Galb1-4GlcNAcb1-3Galb1-4GlcNAcb1-2Mana1-3)Manb1-4GlcNAcb1-4(Fuca1-6)GlcNAcb-Sp19	36	70
572	Galb1-4GlcNAcb1-3Galb1-4GlcNAcb1-6(Galb1-4GlcNAcb1-3Galb1-4GlcNAb1-2)Mana1-6(Galb1-4GlcNAcb1-3Galb1-4GlcNAcb1-2Mana1-3)Manb1-4GlcNAcb1-4(Fuca1-6)GlcNAcb-Sp24	70	82
573	GlcNAcb1-3Galb1-4GlcNAcb1-3Galb1-4GlcNAcb1-6(GlcNAcb1-3Galb1-4GlcNAcb1-3Galb1-4GlcNAb1-2)Mana1-6(GlcNAcb1-3Galb1-4GlcNAcb1-3Galb1-4GlcNAcb1-2Mana1-3)Manb1-4GlcNAcb1-4(Fuca1-6)GlcNAcb-Sp24	38	42
574	Galb1-4GlcNAcb1-3Galb1-4GlcNAcb1-3Galb1-4GlcNAcb1-6(Galb1-4GlcNAcb1-3Galb1-4GlcNAcb1-3Galb1-4GlcNAb1-2)Mana1-6(Galb1-4GlcNAcb1-3Galb1-4GlcNAcb1-3Galb1-4GlcNAcb1-2Mana1-3)Manb1-4GlcNAcb1-4(Fuca1-6)GlcNAcb-Sp24	110	70
575	GlcNAcb1-3Galb1-4GlcNAcb1-3Galb1-4GlcNAcb1-3Galb1-4GlcNAcb1-6(GlcNAcb1-3Galb1-4GlcNAcb1-3Galb1-4GlcNAcb1-3Galb1-4GlcNAb1-2)Mana1-6(GlcNAcb1-3Galb1-4GlcNAcb1-3Galb1-4GlcNAcb1-3Galb1-4GlcNAcb1-2Mana1-3)Manb1-4GlcNAcb1-4(Fuca1-6)GlcNAcb-Sp24	34	42
576	Galb1-4GlcNAcb1-3Galb1-4GlcNAcb1-3Galb1-4GlcNAcb1-3Galb1-4GlcNAcb1-6(Galb1-4GlcNAcb1-3Galb1-4GlcNAcb1-3Galb1-4GlcNAcb1-3Galb1-4GlcNAb1-2)Mana1-6(Galb1-4GlcNAcb1-3Galb1-4GlcNAcb1-3Galb1-4GlcNAcb1-3Galb1-4GlcNAcb1-2Mana1-3)Manb1-4GlcNAcb1-4(Fuca1-6)GlcNAcb-Sp24	24	62
577	GlcNAcb1-3Galb1-4GlcNAcb1-3Galb1-4GlcNAcb1-3Galb1-4GlcNAcb1-3Galb1-4GlcNAcb1-6(GlcNAcb1-3Galb1-4GlcNAcb1-3Galb1-4GlcNAcb1-3Galb1-4GlcNAcb1-3Galb1-4GlcNAb1-2)Mana1-6(GlcNAcb1-3Galb1-4GlcNAcb1-3Galb1-4GlcNAcb1-3Galb1-4GlcNAcb1-3Galb1-4GlcNAcb1-2Mana1-3)Manb1-4GlcNAcb1-4(Fuca1-6)GlcNAcb-Sp24	94	190
578	Galb1-4GlcNAcb1-3Galb1-4GlcNAcb1-3Galb1-4GlcNAcb1-3Galb1-4GlcNAcb1-3Galb1-4GlcNAcb1-6(Galb1-4GlcNAcb1-3Galb1-4GlcNAcb1-3Galb1-4GlcNAcb1-3Galb1-4GlcNAcb1-3Galb1-4GlcNAb1-2)Mana1-6(Galb1-4GlcNAcb1-3Galb1-4GlcNAcb1-3Galb1-4GlcNAcb1-	79	123

	3Galb1-4GlcNAcb1-3Galb1-4GlcNAcb1-2Mana1-3)Manb1-4GlcNAcb1-4(Fuca1-6)GlcNAcb-Sp24		
579	Galb1-4GlcNAcb1-3Galb1-4GlcNAcb1-3GalNAca-Sp14	273	138
580	Galb1-4GlcNAcb1-3Galb1-4GlcNAcb1-6(Galb1-3)GalNAca-Sp14	285	180
581	Galb1-4GlcNAcb1-3Galb1-4GlcNAcb1-6(Galb1-4GlcNAcb1-3Galb1-4GlcNAcb1-3)GalNAca-Sp14	483	239
582	Neu5Aca2-3Galb1-4GlcNAcb1-3Galb1-4GlcNAcb1-3GalNAca-Sp14	-7	-8
583	GlcNAcb1-3Galb1-4GlcNAcb1-3GalNAca-Sp14	-15	-7
584	GlcNAcb1-3Galb1-4GlcNAcb1-6(Galb1-3)GalNAca-Sp14	2	0
585	GlcNAcb1-3Galb1-4GlcNAcb1-6(GlcNAcb1-3Galb1-4GlcNAcb1-3)GalNAca-Sp14	-1	1
586	Neu5Aca2-3Galb1-4GlcNAcb1-3Galb1-4GlcNAcb1-6(Neu5Aca2-3Galb1-4GlcNAcb1-3Galb1-4GlcNAcb1-3)GalNAca-Sp14	39	23
587	Neu5Aca2-6Galb1-4GlcNAcb1-3Galb1-4GlcNAcb1-3GalNAca-Sp14	10	7
588	GlcNAcb1-3Galb1-4GlcNAcb1-3Galb1-4GlcNAcb1-3GalNAca-Sp14	8	1
589	Galb1-4GlcNAcb1-3Galb1-3GalNAca-Sp14	1	2
590	Neu5Aca2-3Galb1-4GlcNAcb1-3Galb1-4GlcNAcb1-6(Galb1-3)GalNAca-Sp14	101	59
591	Neu5Aca2-6Galb1-4GlcNAcb1-3Galb1-4GlcNAcb1-6(Galb1-3)GalNAca-Sp14	45	189
592	Neu5Aca2-6Galb1-4GlcNAcb1-6(Galb1-3)GalNAca-Sp14	8	8
593	Neu5Aca2-3Galb1-4GlcNAcb1-3Galb1-4GlcNAcb1-2Mana1-6(Neu5Aca2-3Galb1-4GlcNAcb1-3Galb1-4GlcNAcb1-2Mana1-3)Manb1-4GlcNAcb1-4GlcNAcb-Sp12	227	128
594	GlcNAcb1-6(Neu5Aca2-3Galb1-3)GalNAca-Sp14	2	1
595	Neu5Aca2-6Galb1-4GlcNAcb1-3Galb1-4GlcNAcb1-6(Neu5Aca2-6Galb1-4GlcNAcb1-3Galb1-4GlcNAcb1-3)GalNAca-Sp14	141	73
596	Neu5Aca2-6Galb1-4GlcNAcb1-3Galb1-4GlcNAcb1-3Galb1-4GlcNAcb1-2Mana1-6(Neu5Aca2-6Galb1-4GlcNAcb1-3Galb1-4GlcNAcb1-3Galb1-4GlcNAcb1-2Mana1-3)Manb1-4GlcNAcb1-4GlcNAcb-Sp12	443	296
597	Neu5Aca2-3Galb1-4GlcNAcb1-3Galb1-4GlcNAcb1-3Galb1-4GlcNAcb1-2Mana1-6(Neu5Aca2-3Galb1-4GlcNAcb1-3Galb1-4GlcNAcb1-3Galb1-4GlcNAcb1-2Mana1-3)Manb1-4GlcNAcb1-4GlcNAcb-Sp12	684	490
598	Neu5Aca2-6Galb1-4GlcNAcb1-3Galb1-4GlcNAcb1-2Mana1-6(Neu5Aca2-6Galb1-4GlcNAcb1-3Galb1-4GlcNAcb1-2Mana1-3)Manb1-4GlcNAcb1-4GlcNAcb-Sp12	119	67
599	GlcNAcb1-3Fuca-Sp21	1	6
600	Galb1-3GalNAcb1-4(Neu5Aca2-8Neu5Aca2-8Neu5Aca2-3)Galb1-4Glcb-Sp21	18	16
