# Supplementary material for: Strengthening of enterococcal biofilms by Esp
Source: PLoS Pathog. 2022 Sep 14;18(9):e1010829. doi: 10.1371/journal.ppat.1010829 (PMC9512215; doi:10.1371/journal.ppat.1010829)
Supplement: S4 Table — (PDF) [file ppat.1010829.s020.pdf]

S4 Table. SEC-MALS-SAX Analysis

SAXS Data	Esp743	Esp452	$Esp_{453\text{-}743}$
Beamline	ALS 12.3.1	ALS 12.3.1	ALS 12.3.1
SEC load (mg/ml)	15	10	10
Porod Debye (Px)	3.8	4.0	2.5
Low q ($Å^{-1}$)	0.01732	0.01111	0.01266
High q ($Å^{-1}$)	0.3677	0.3677	0.36874
Reciprocal Rg (Å)	39	24	33
Real space Rg (Å)	43	25	35
Dmax (Å)	157	85	119
MALS MW (kD)	76	45	31
SAXS MW (kD)	59	40	26
Theoretical MW (kD)	77	46	31
