# Supplementary material for: Strengthening of enterococcal biofilms by Esp
Source: PLoS Pathog. 2022 Sep 14;18(9):e1010829. doi: 10.1371/journal.ppat.1010829 (PMC9512215; doi:10.1371/journal.ppat.1010829)
Supplement: S1 File — Individual Z-stacks of MMH594b (Δesp) biofilms grown overnight with PBS. Bacteria were stained with Syto-13 (green) and proteins were labeled with AF647 (red). The first image was taken at the interface of the biofilm with the slide and each stack is 1.2 μm higher, progressing up to the top of the biofilm at the biofilm-media interface. (PDF) [file ppat.1010829.s021.pdf]

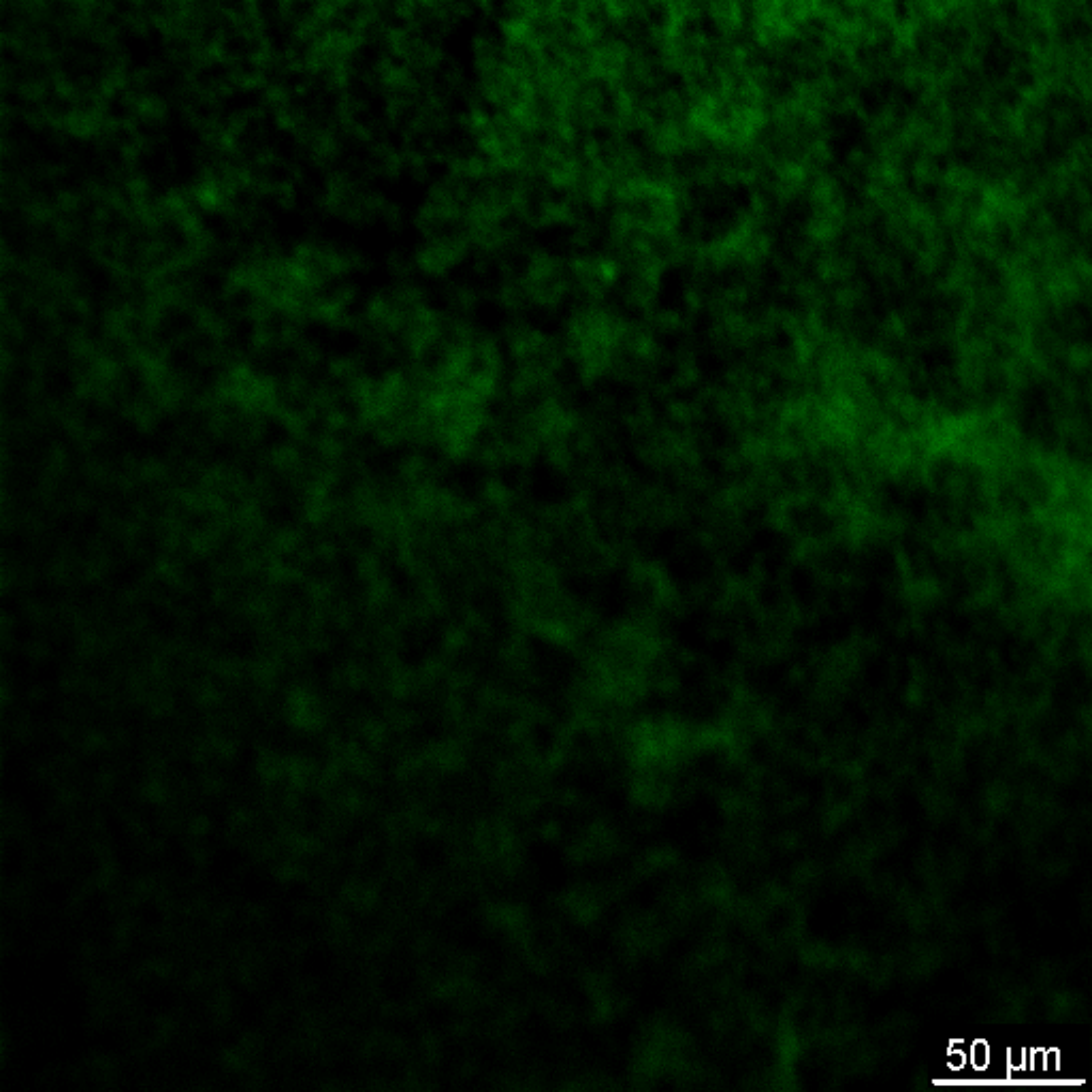
50 µm

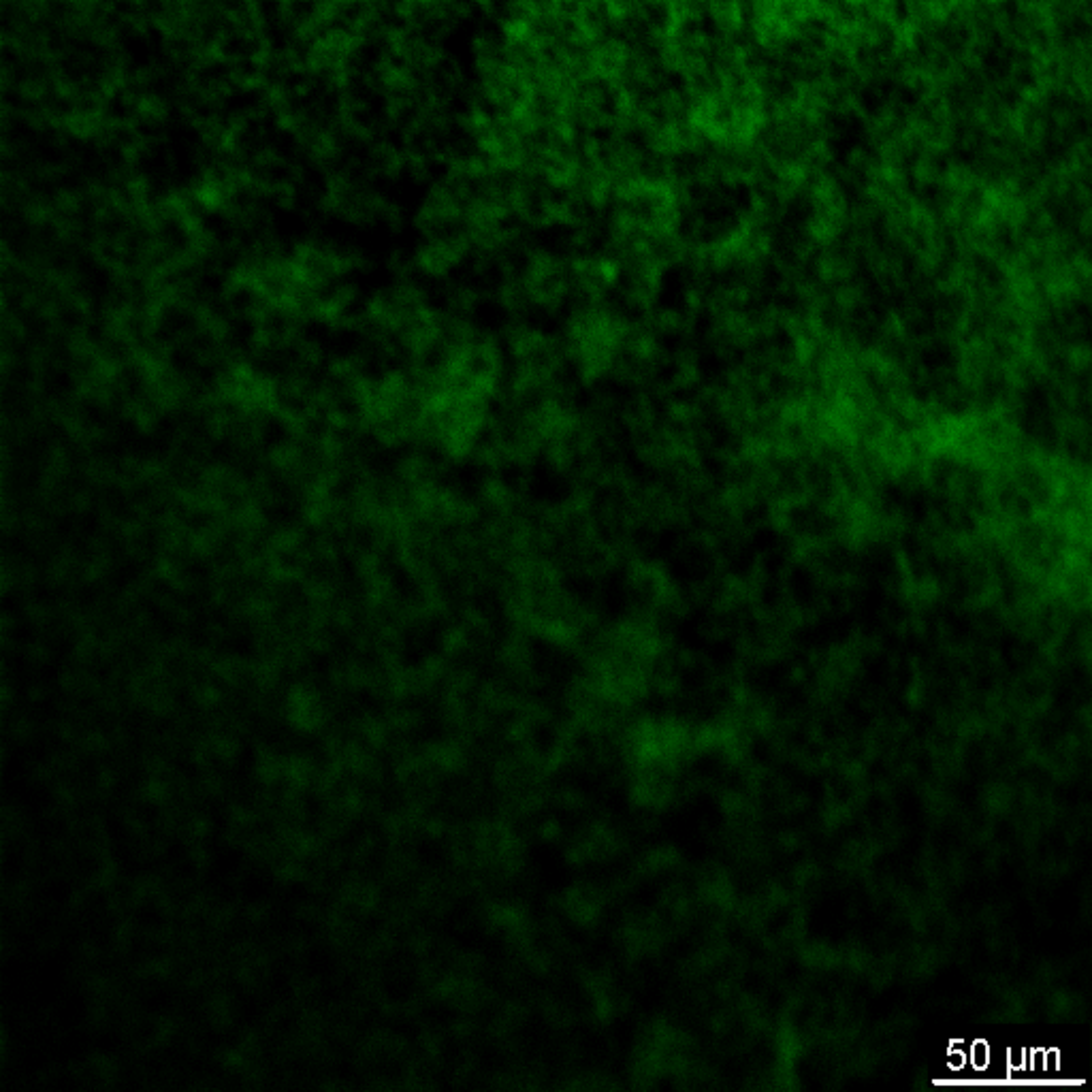
50 µm

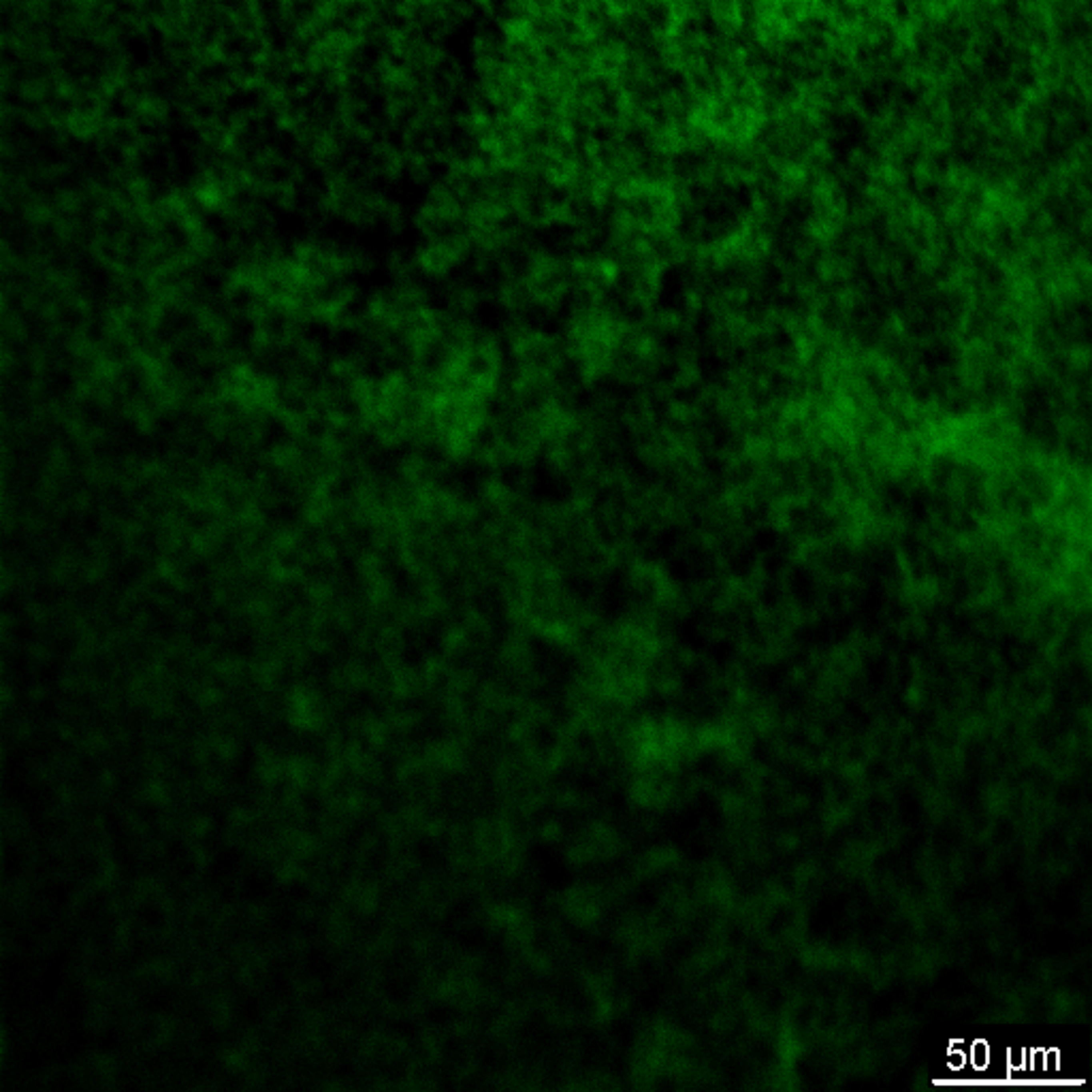
50 µm

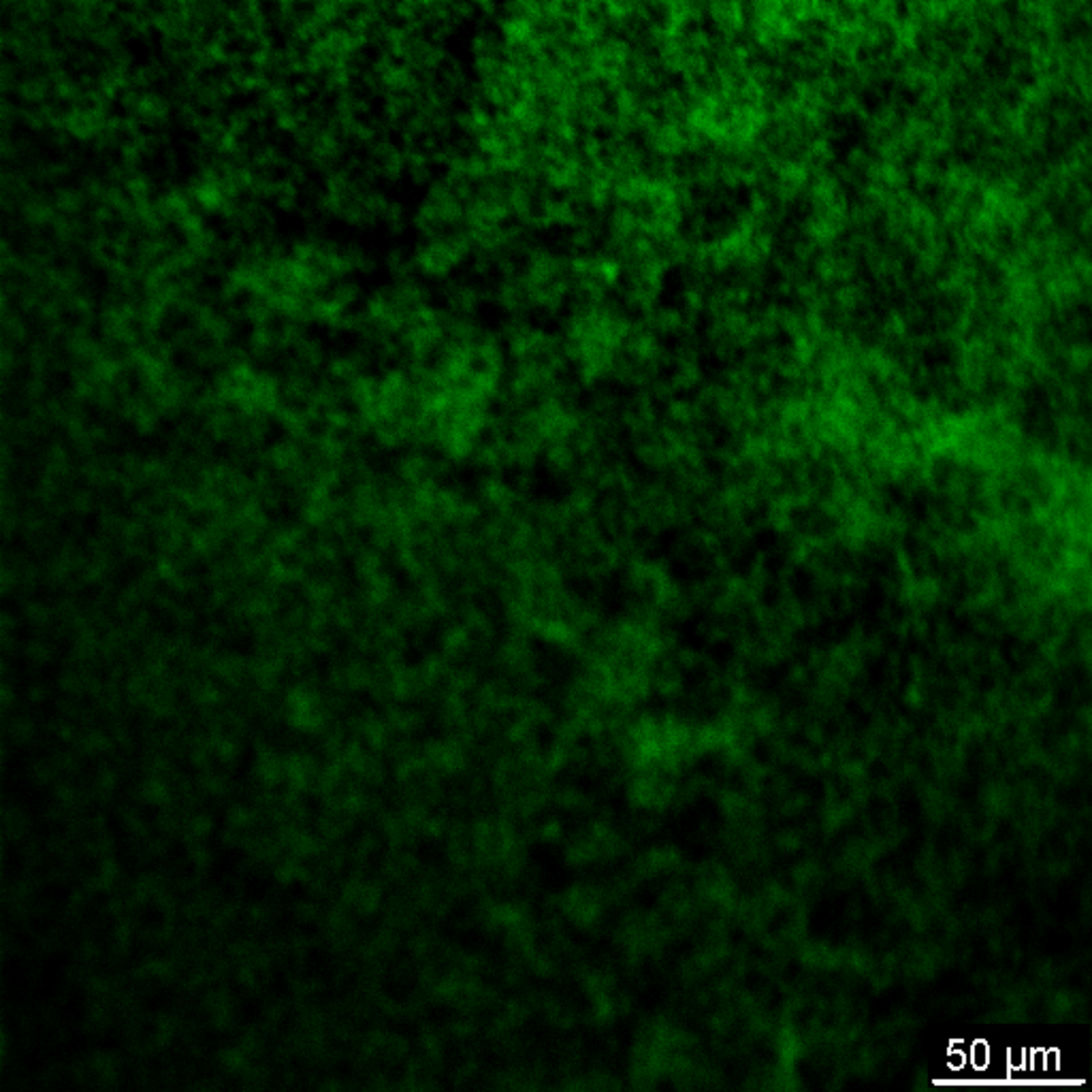
50 µm

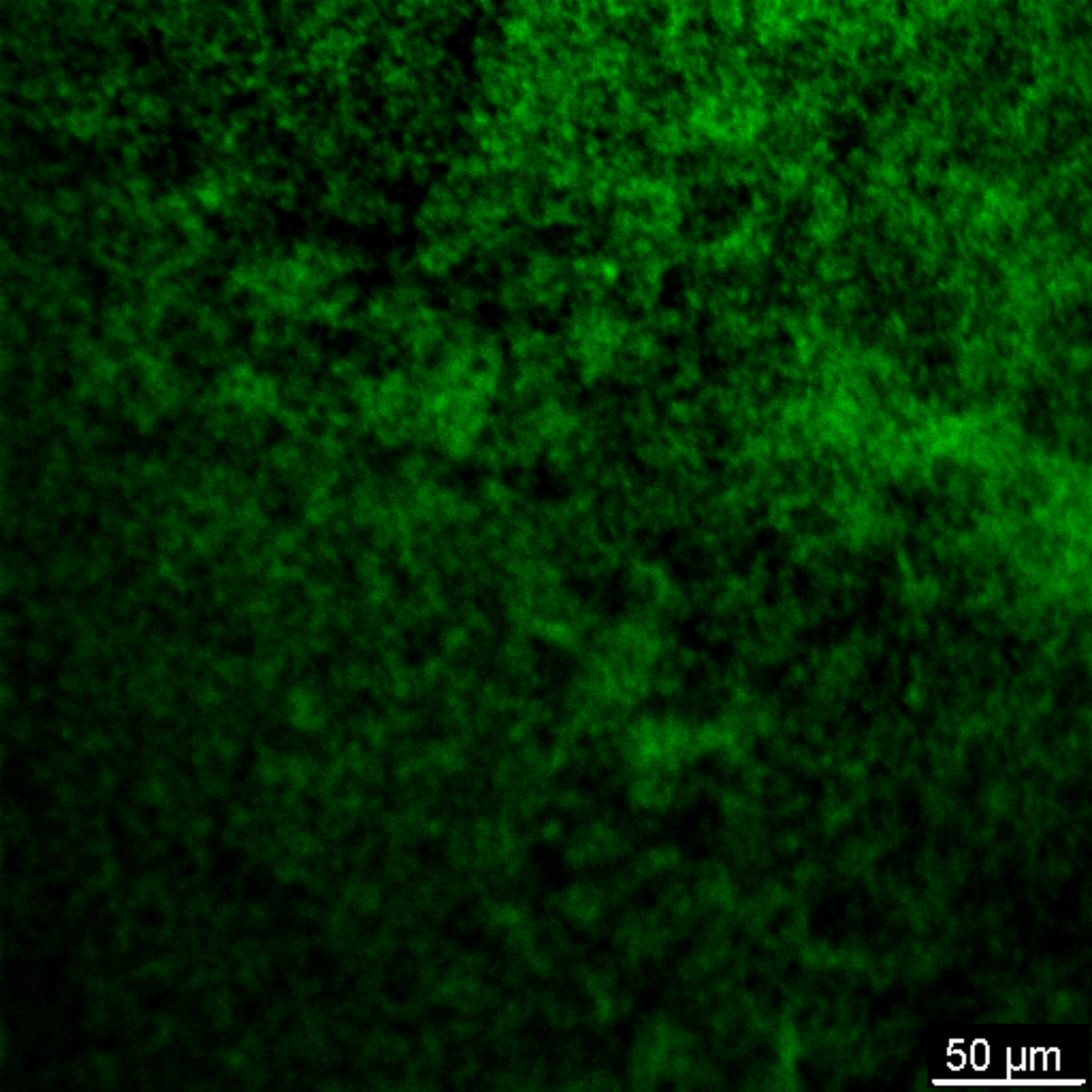
50 µm

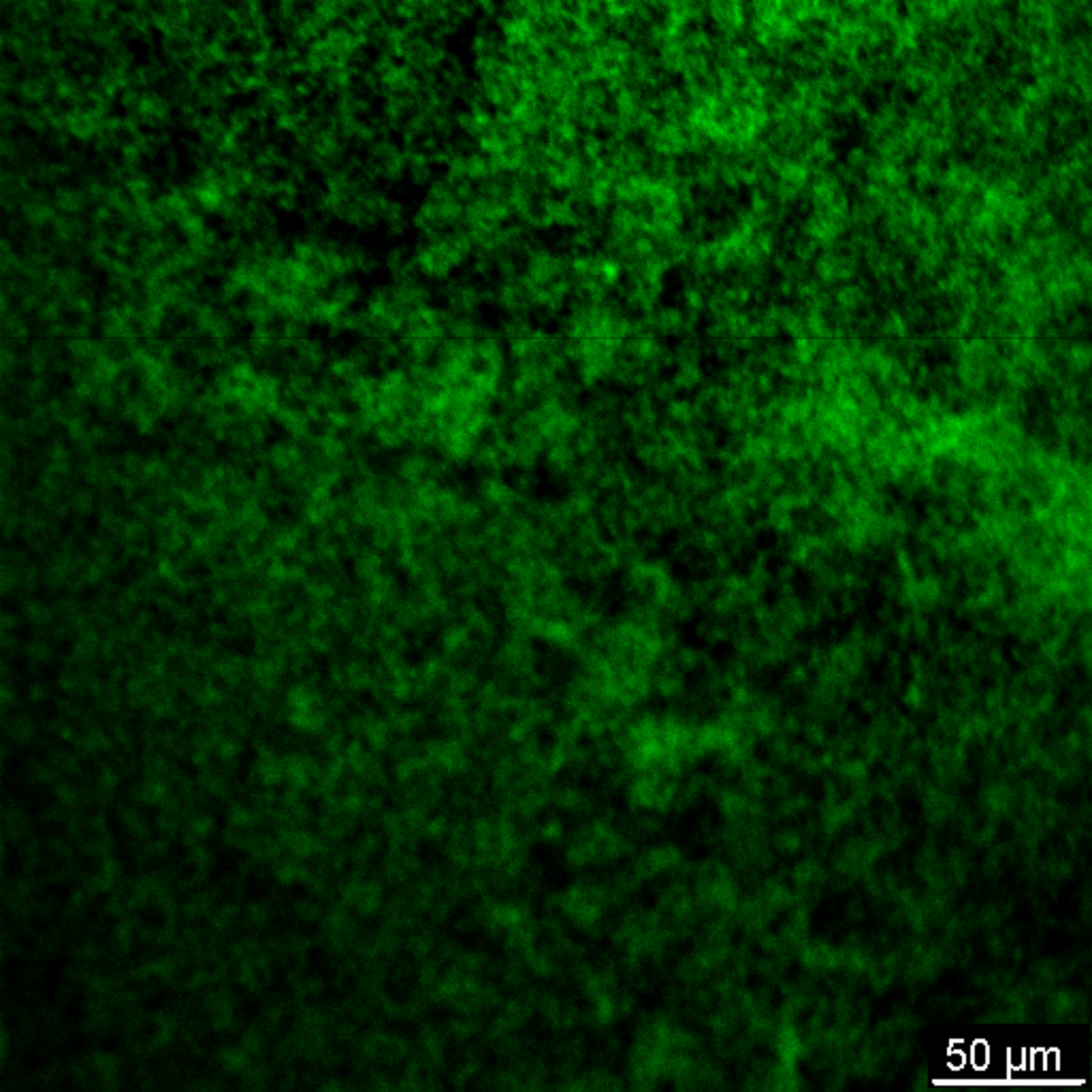
50 µm

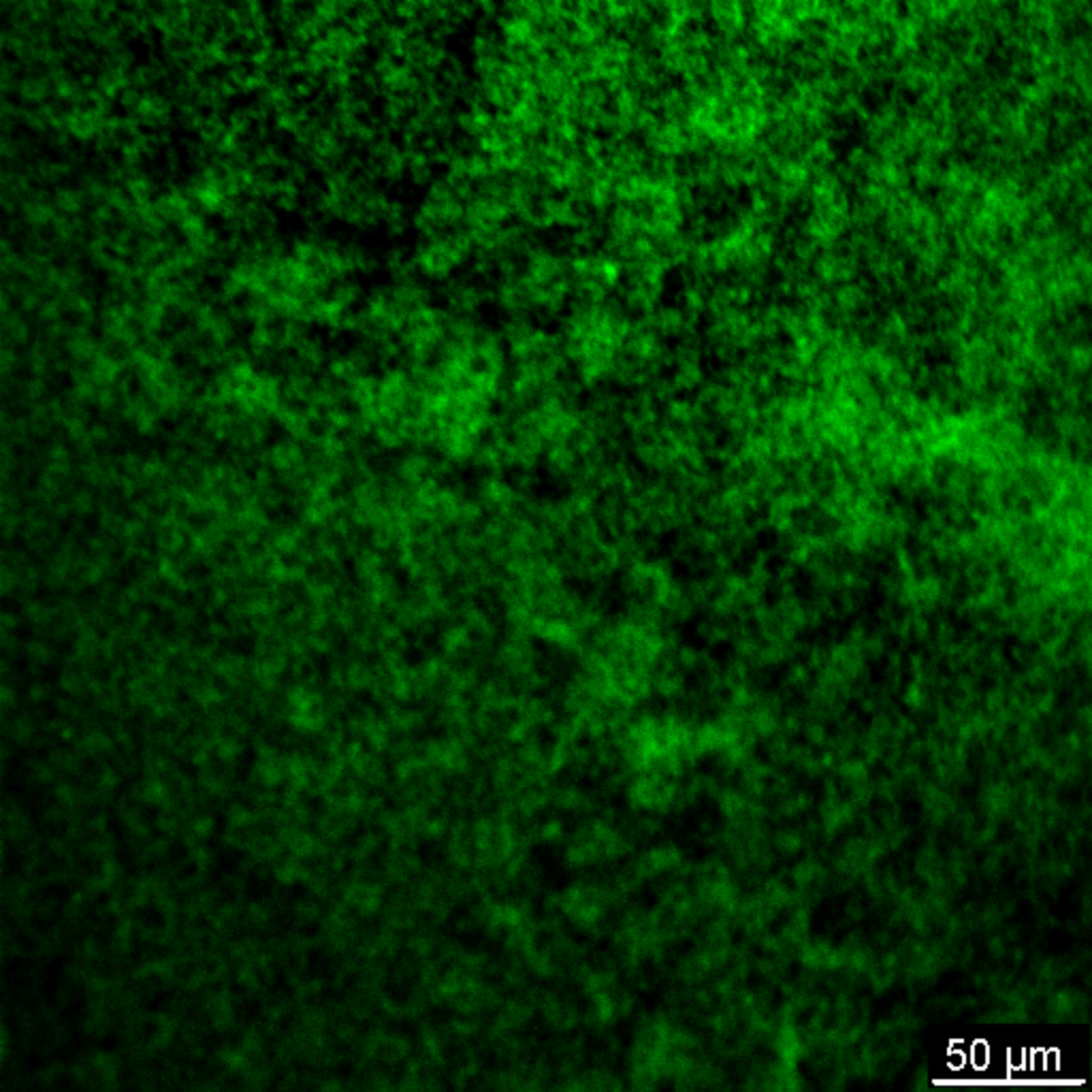
50 μm

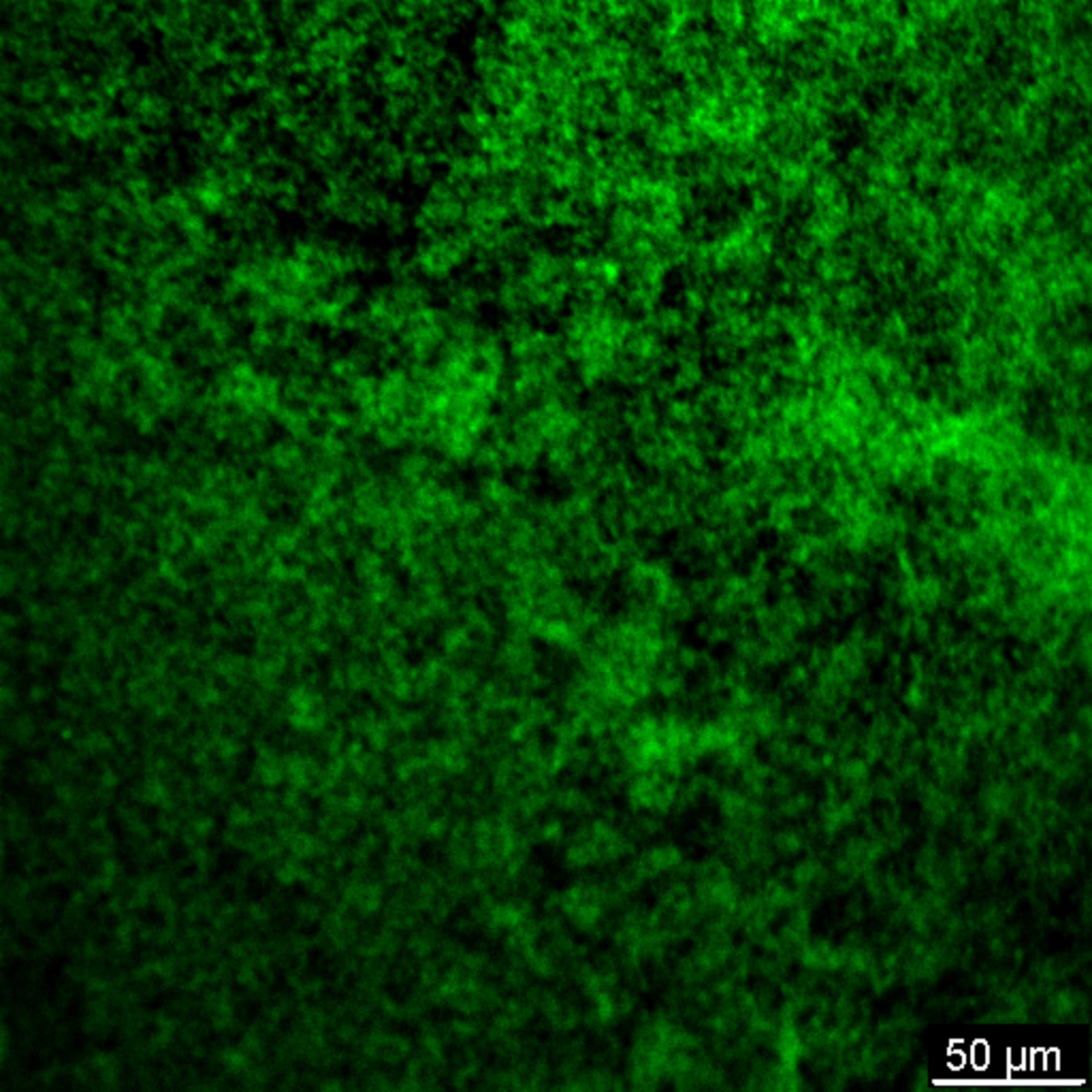
50 µm

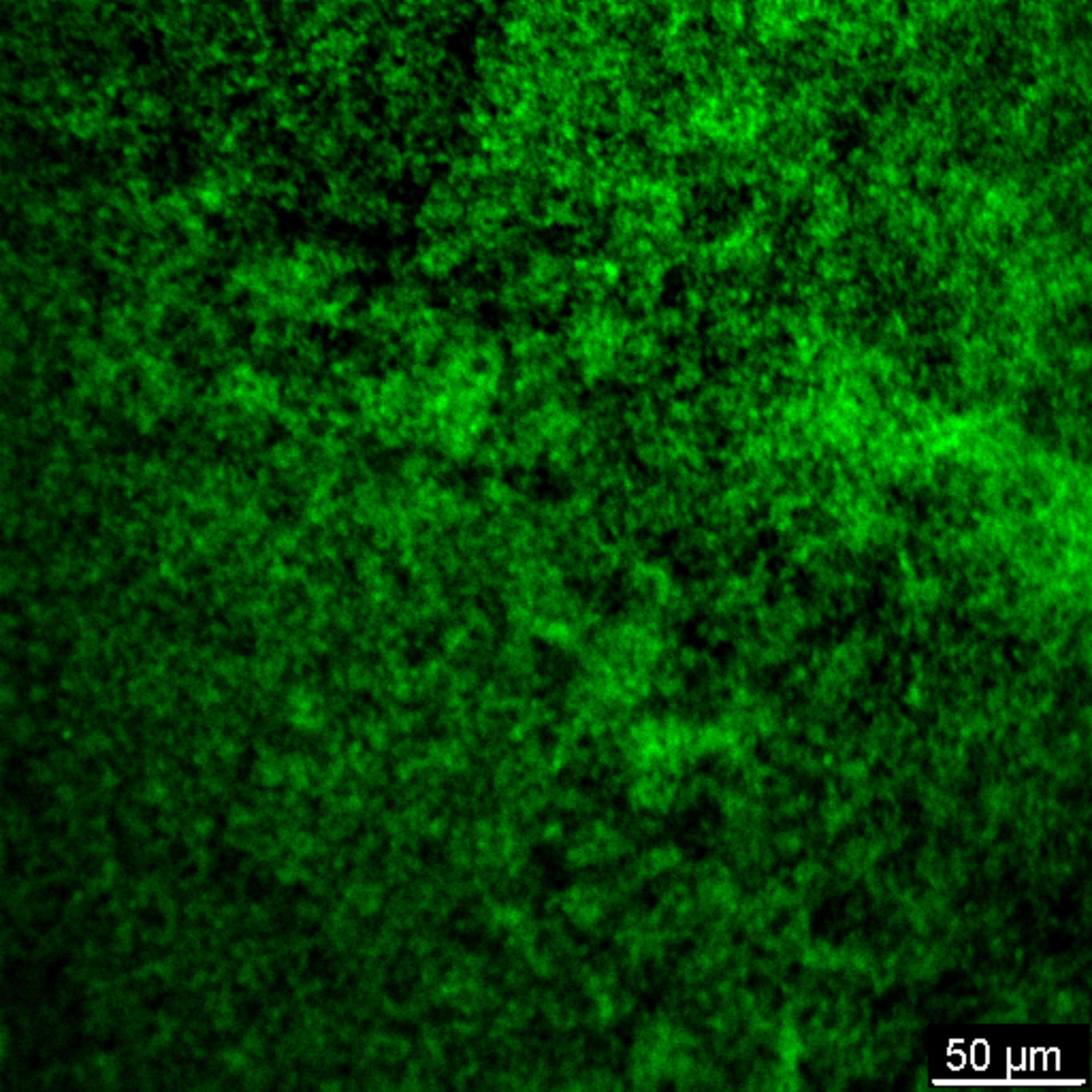
50 µm

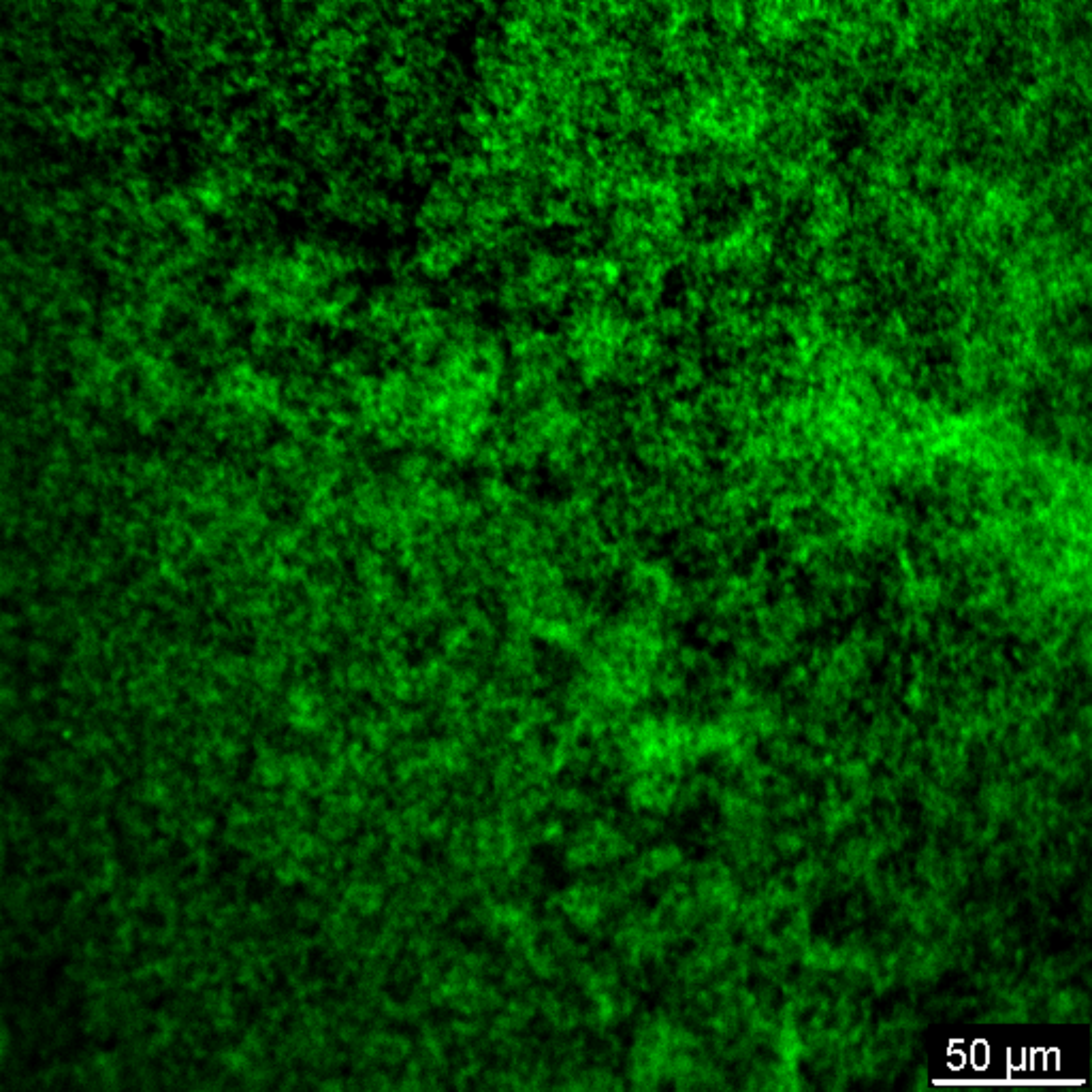
50 µm

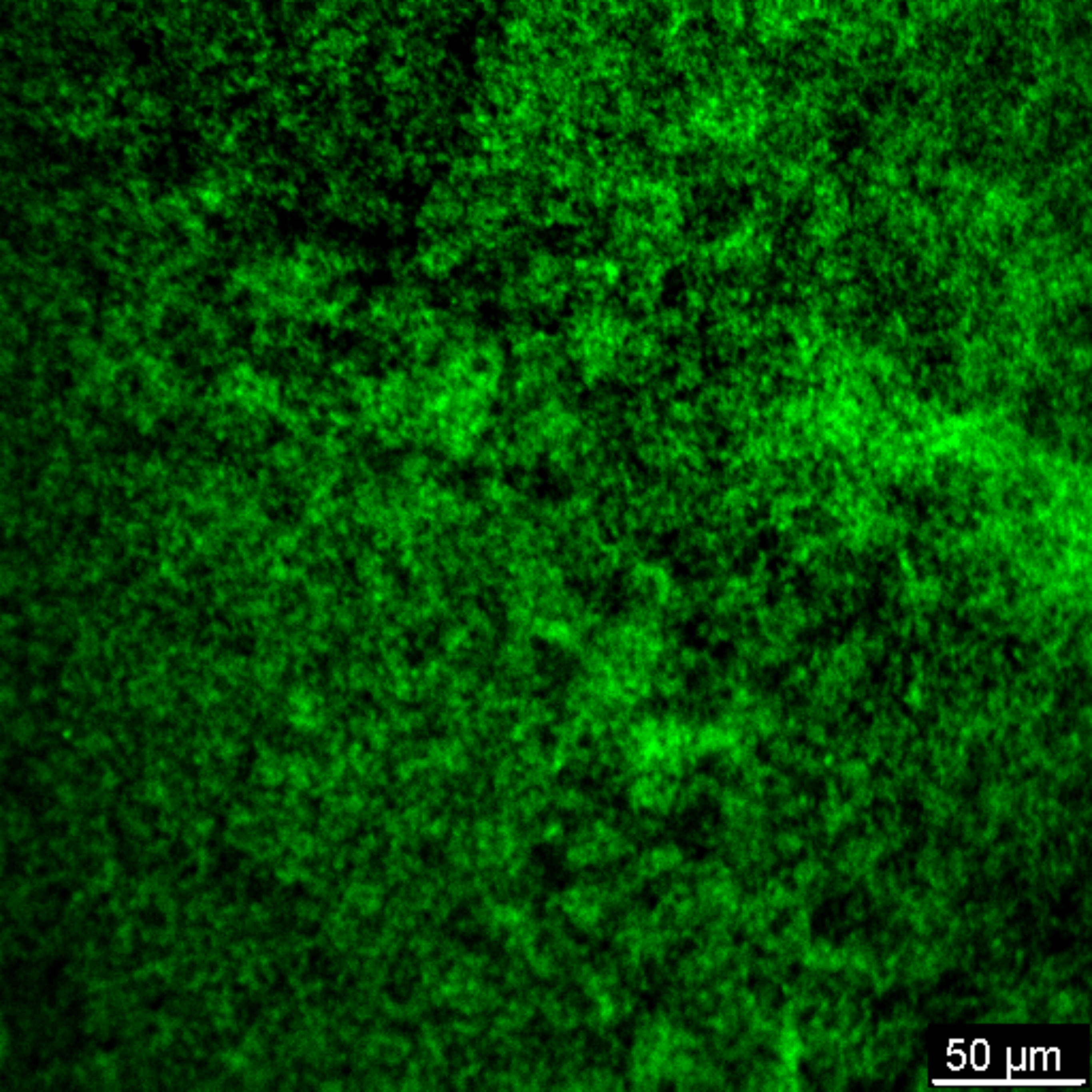
50 µm

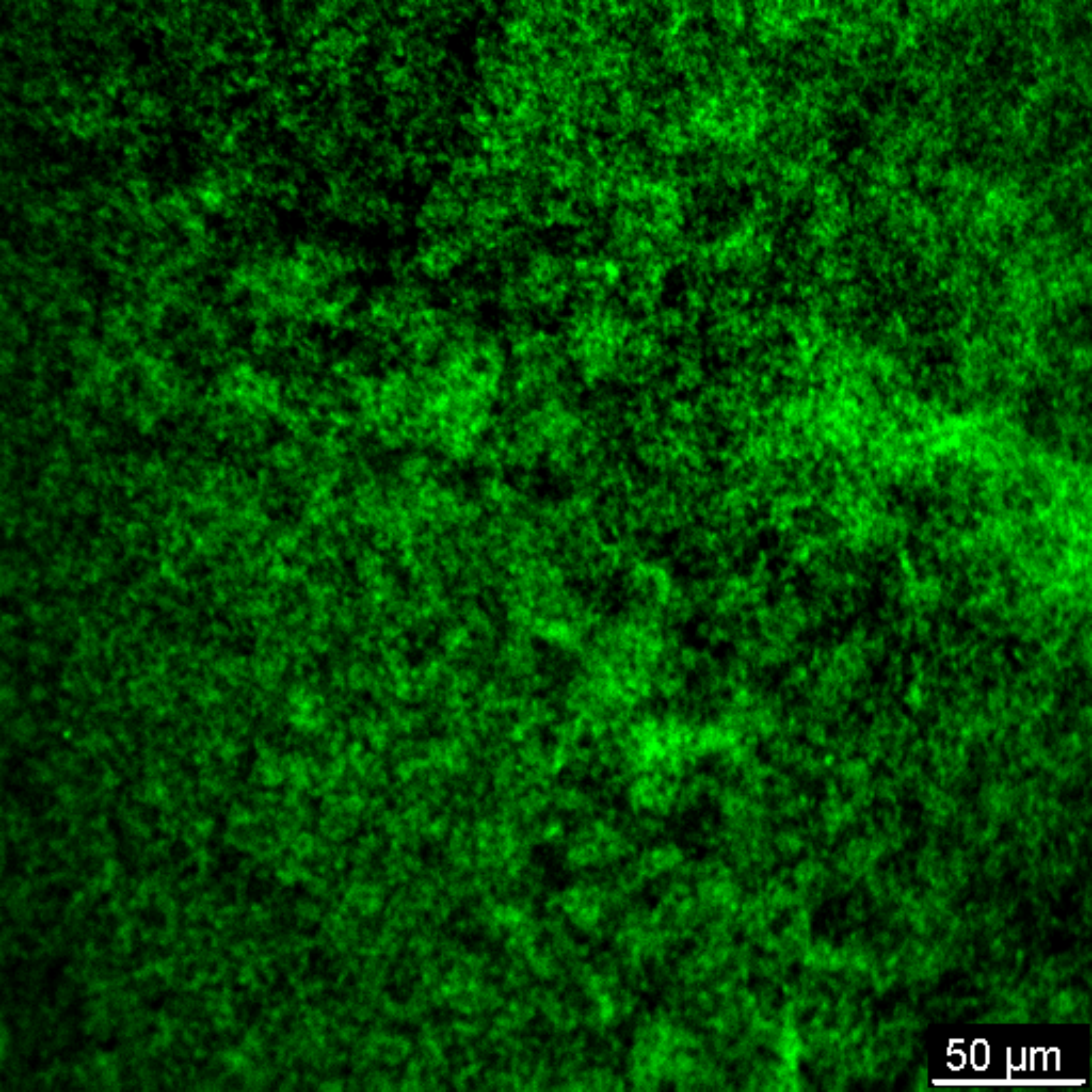
50 µm

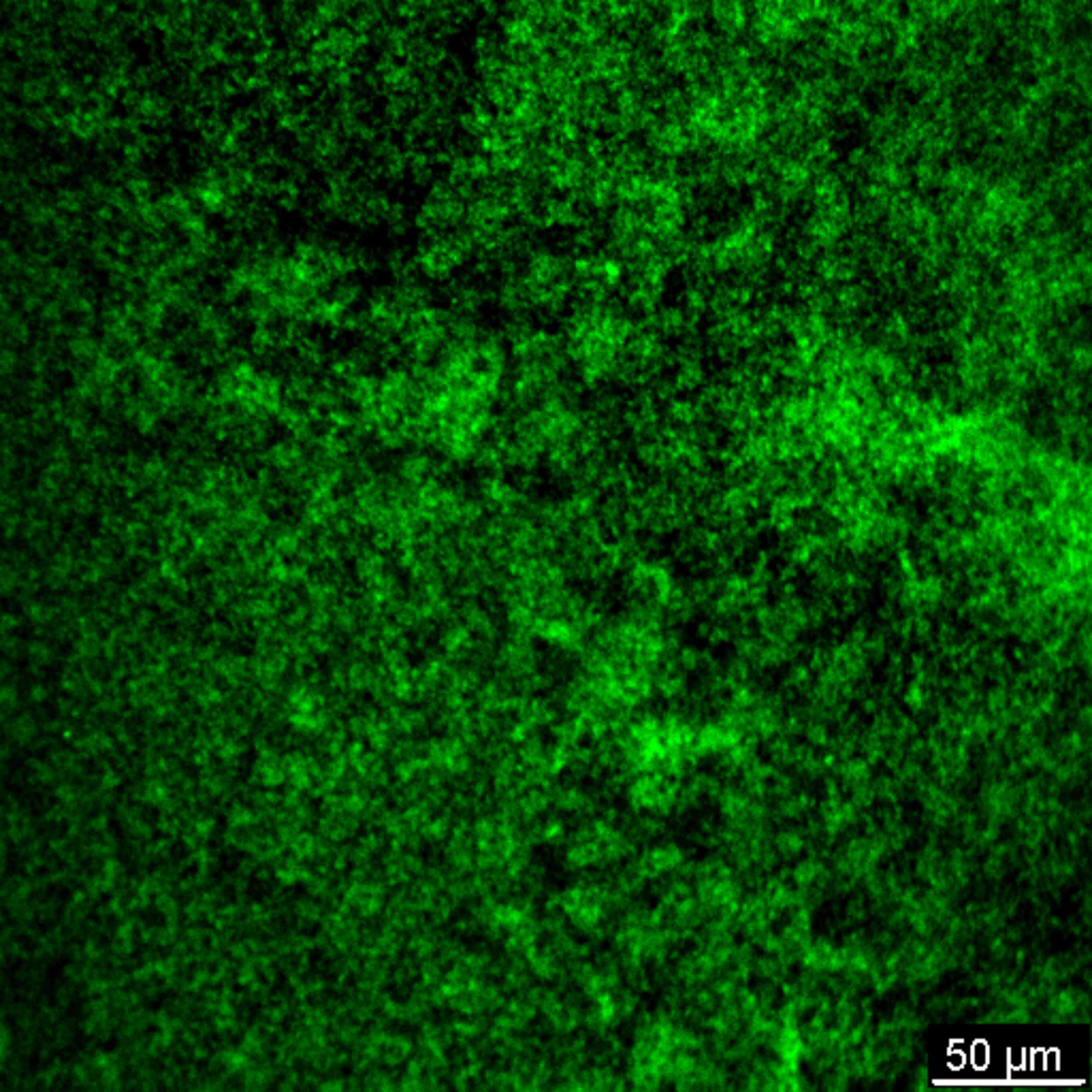
50 µm

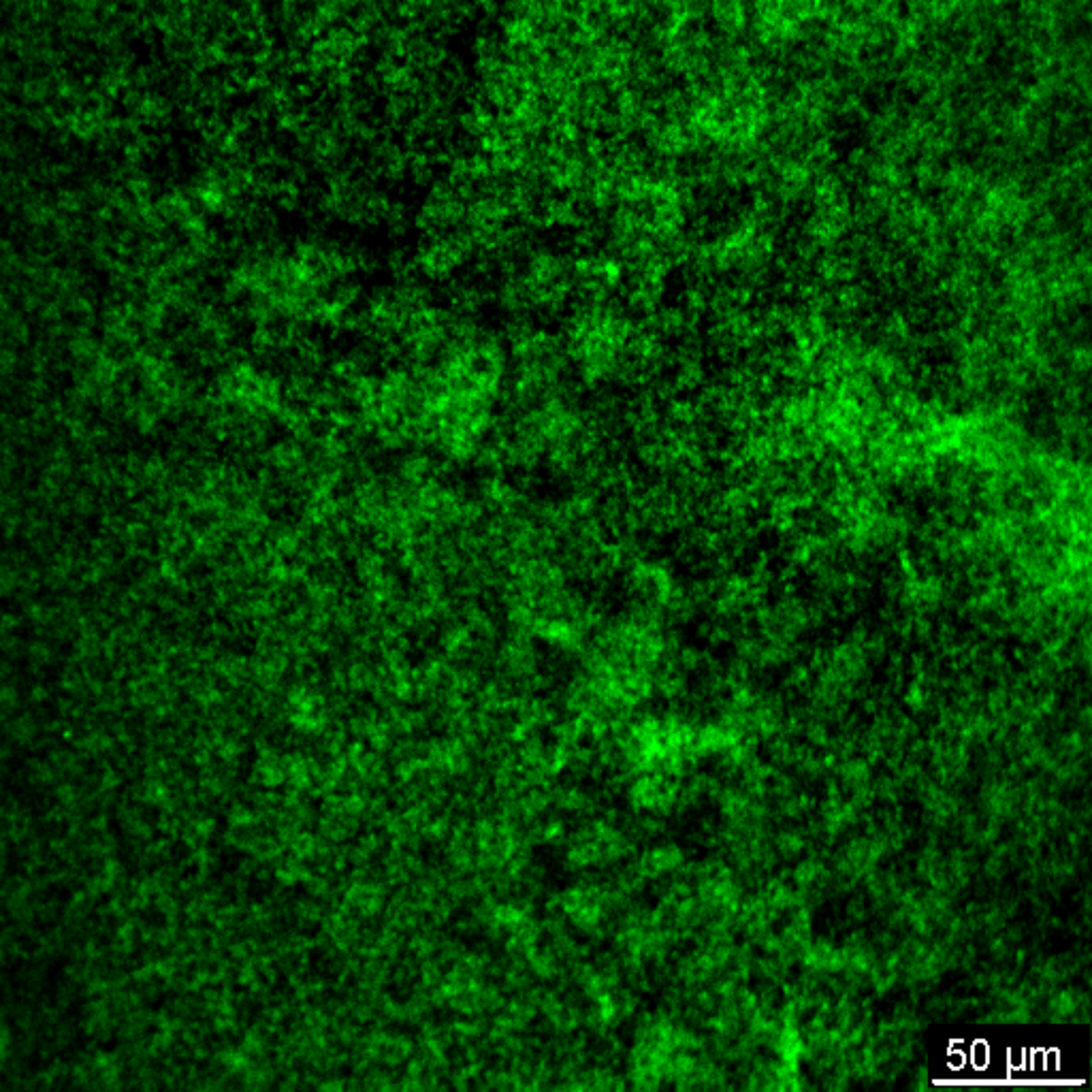
50 µm

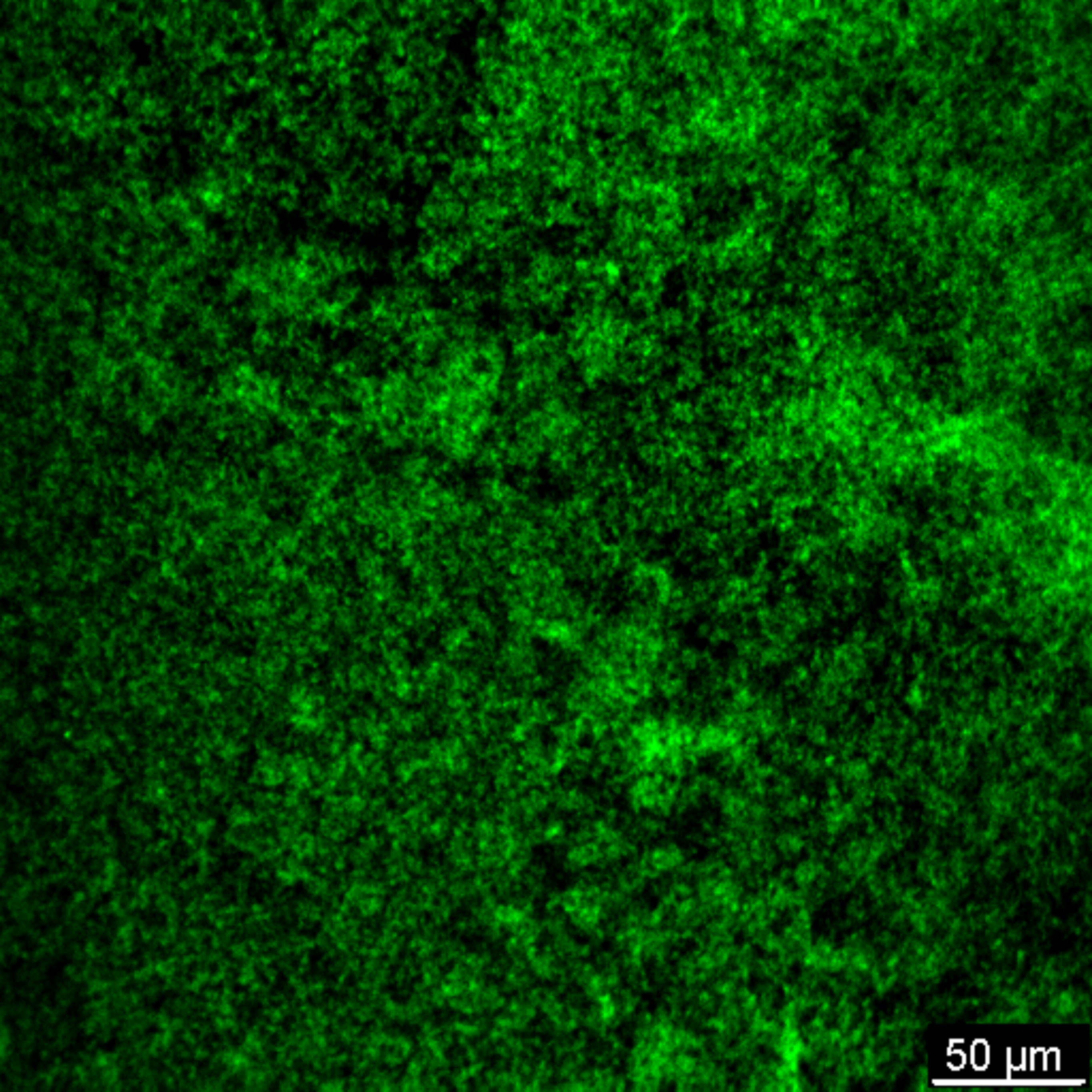
50 µm

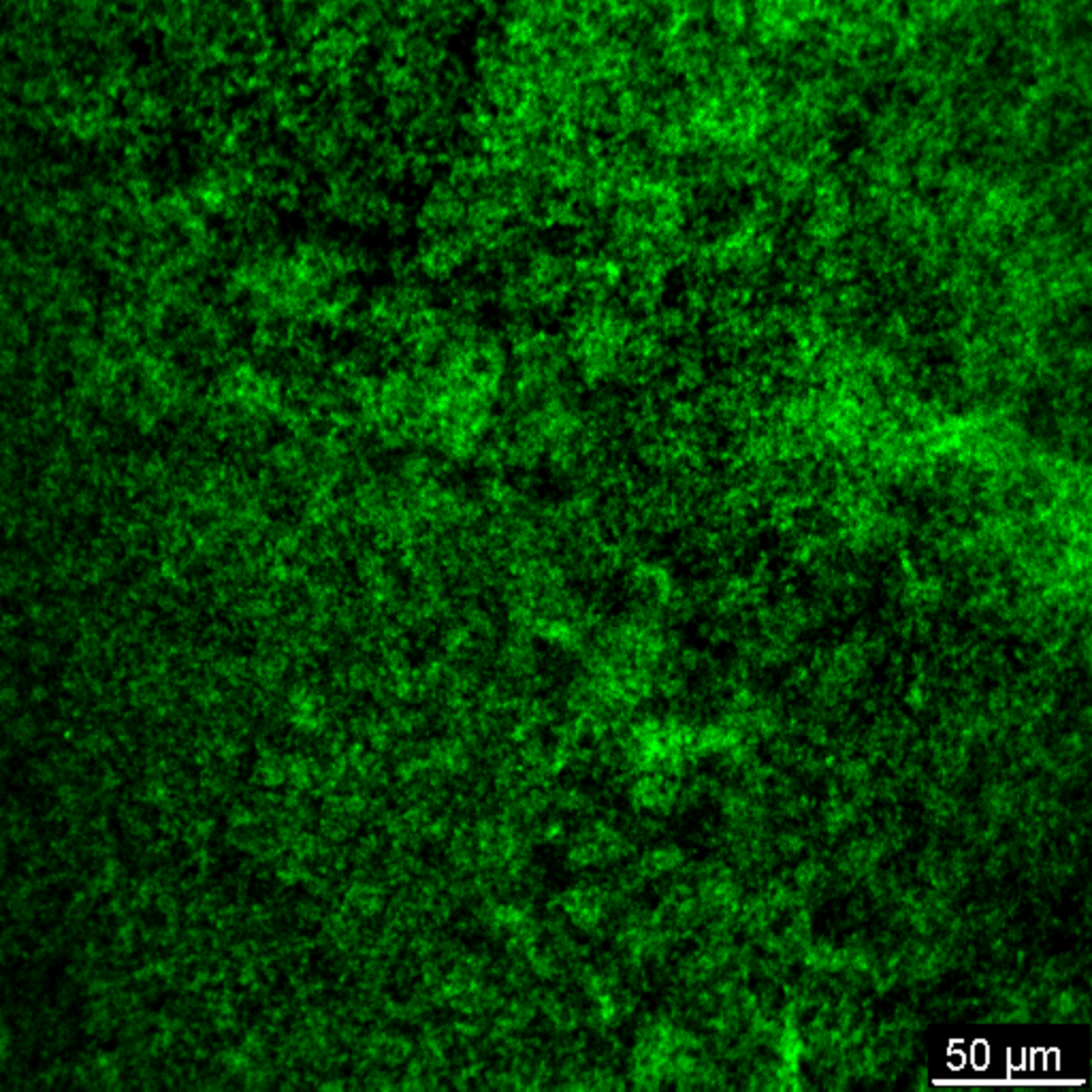
50 µm

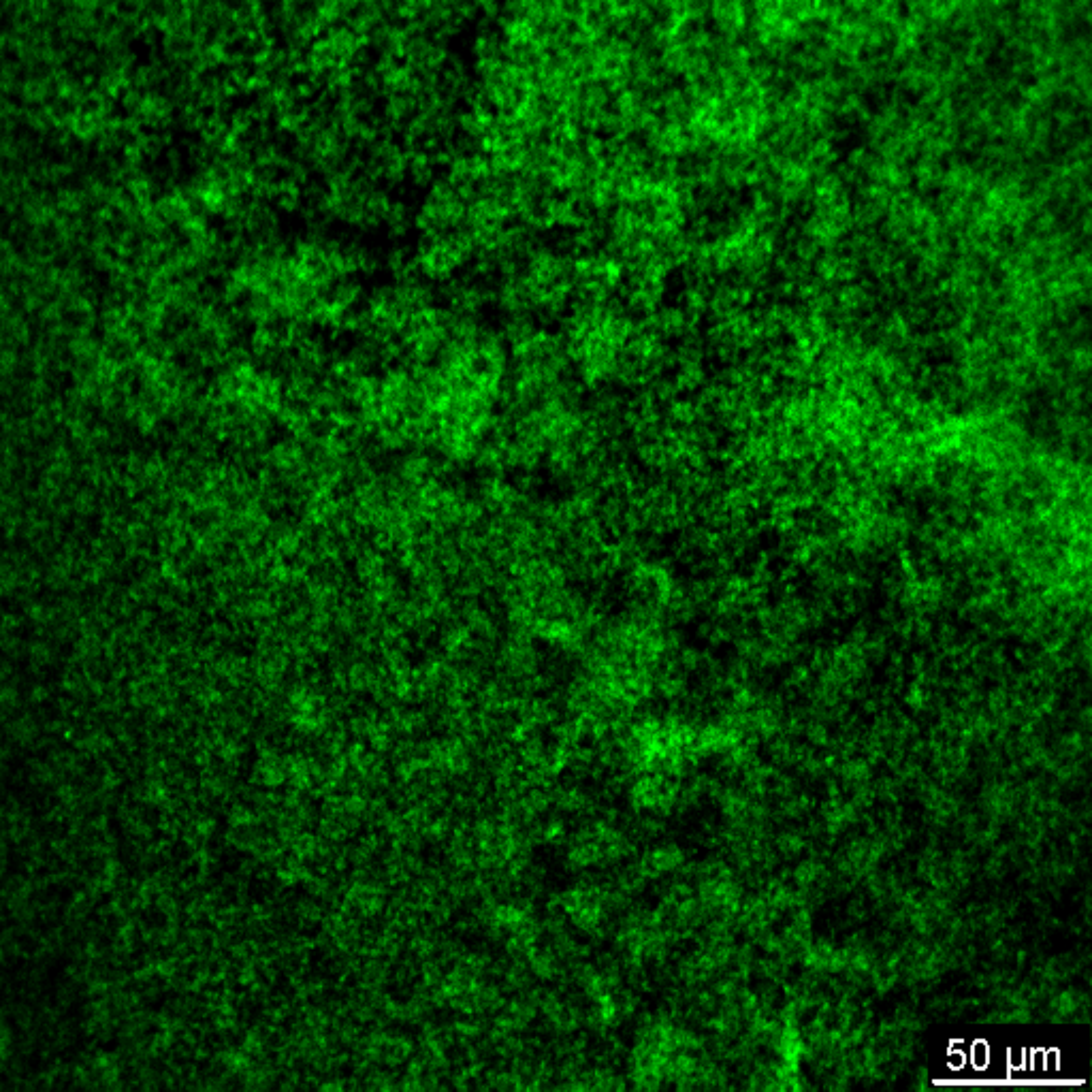
50 µm

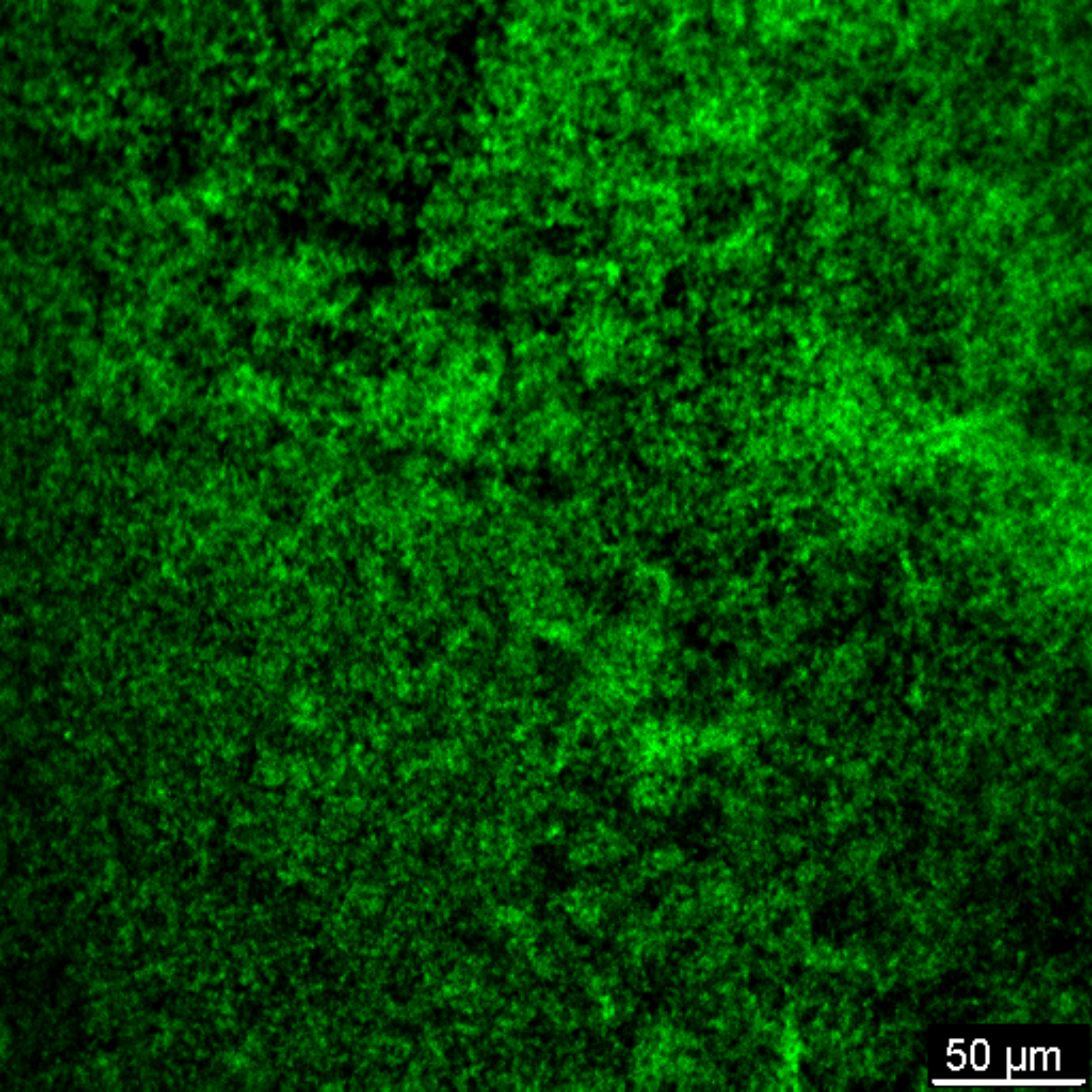
50 µm

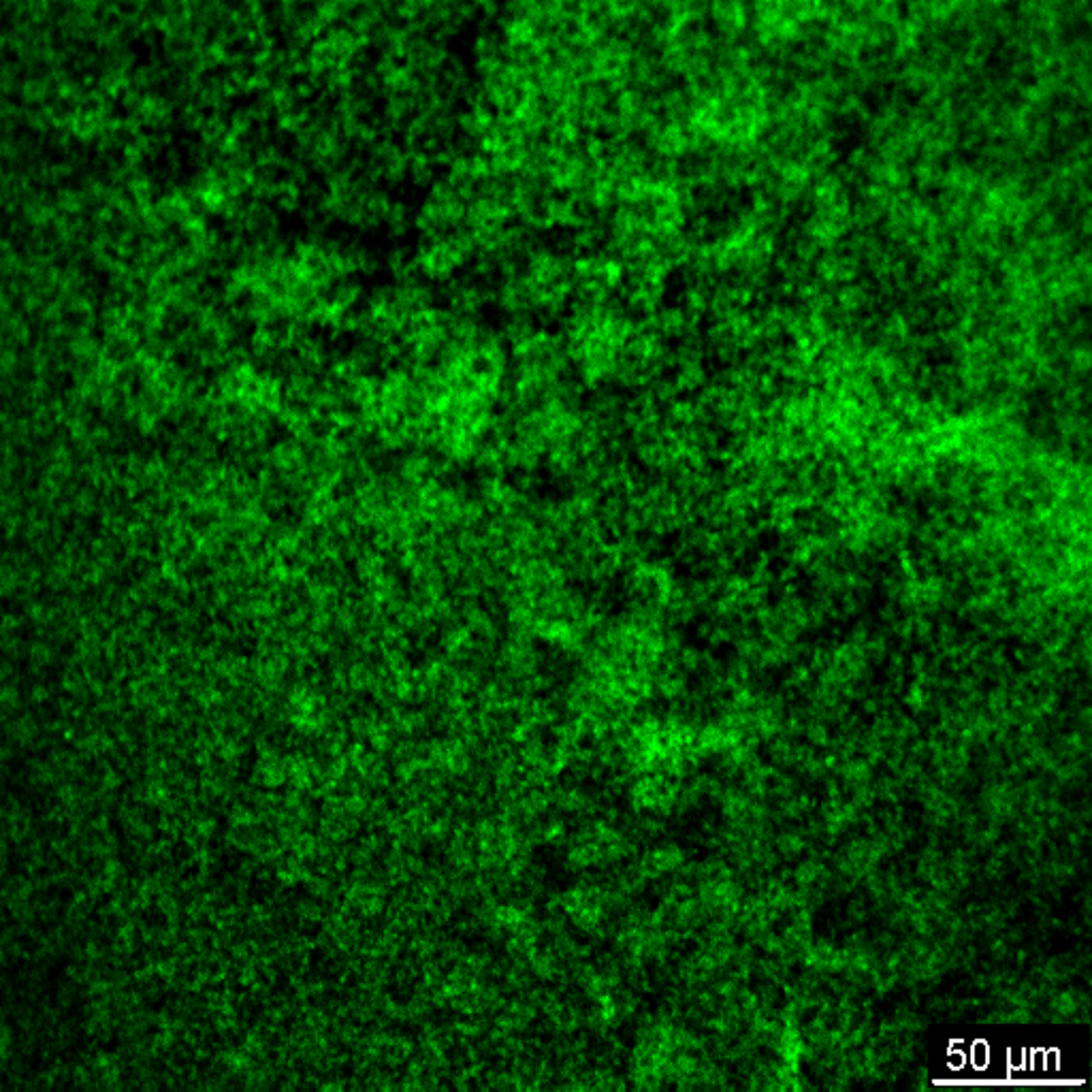
50 µm

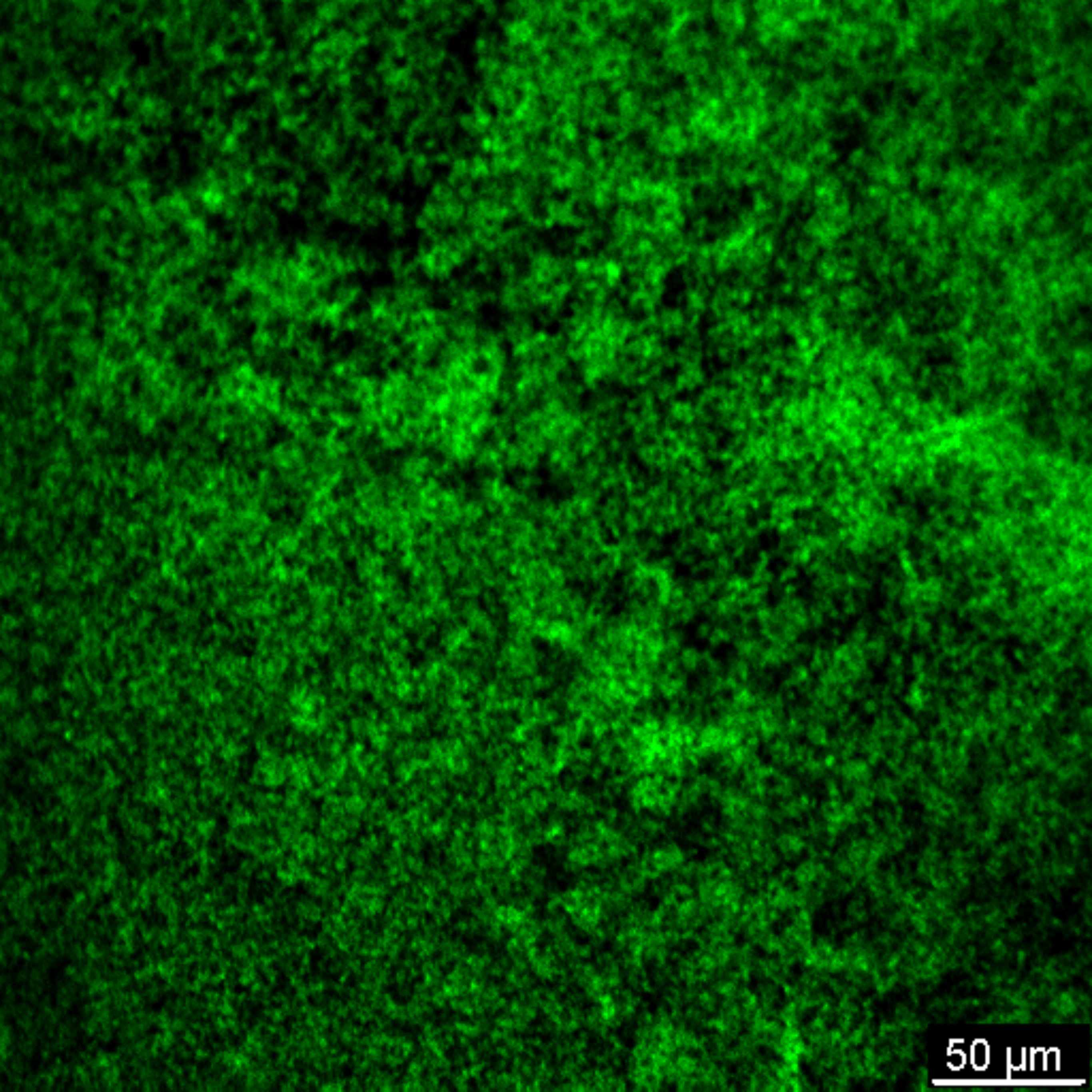
50 µm

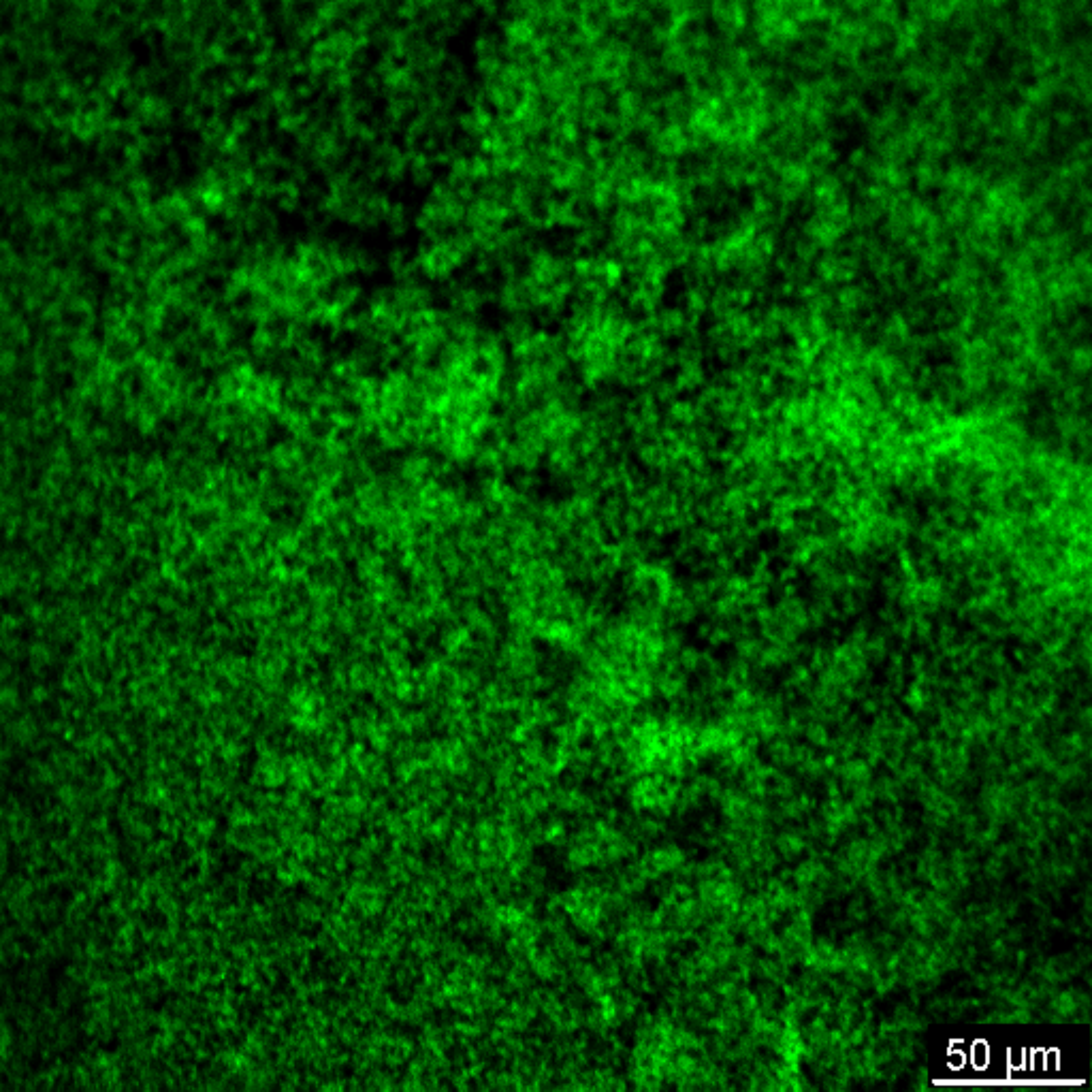
50 µm

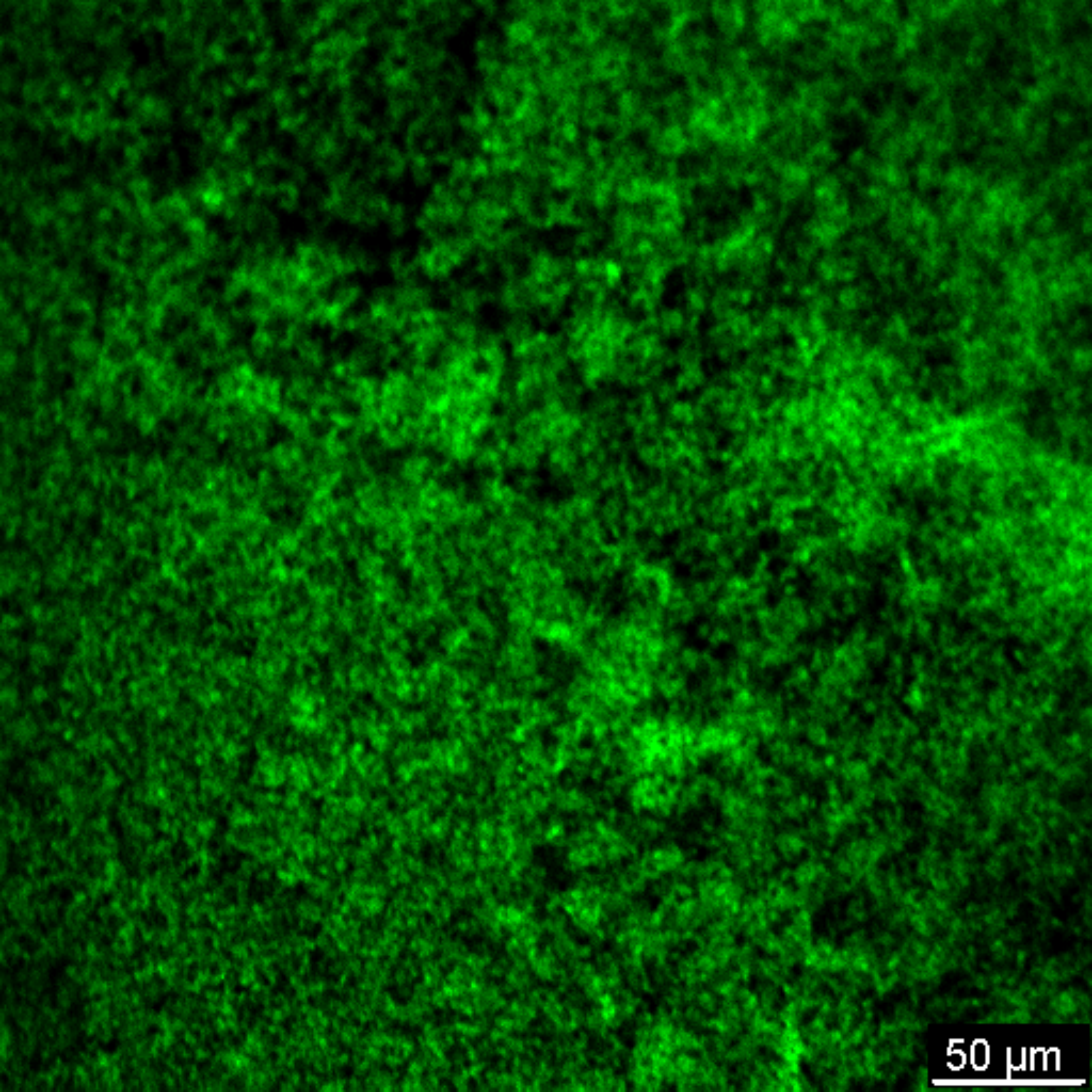
50 µm

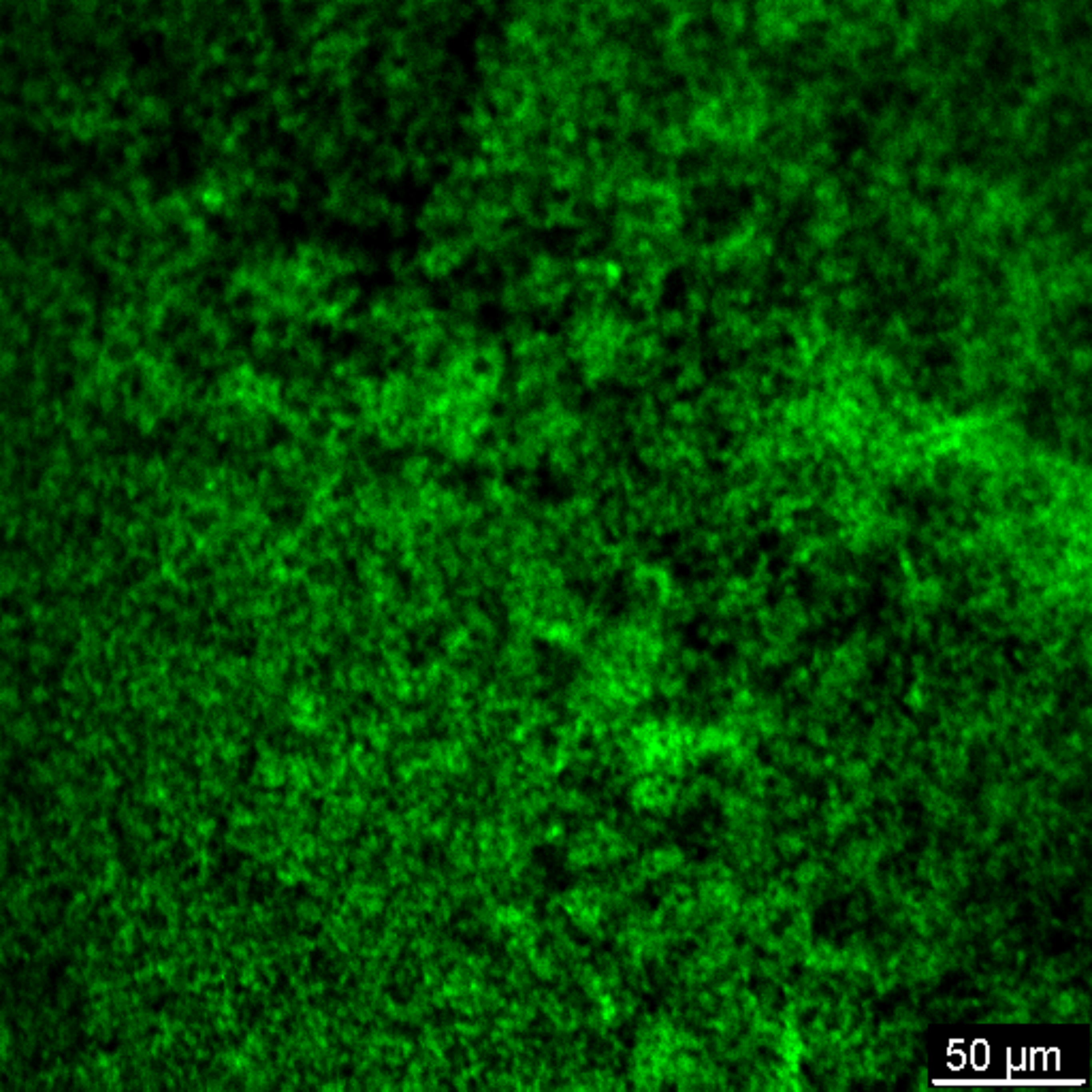
50 µm

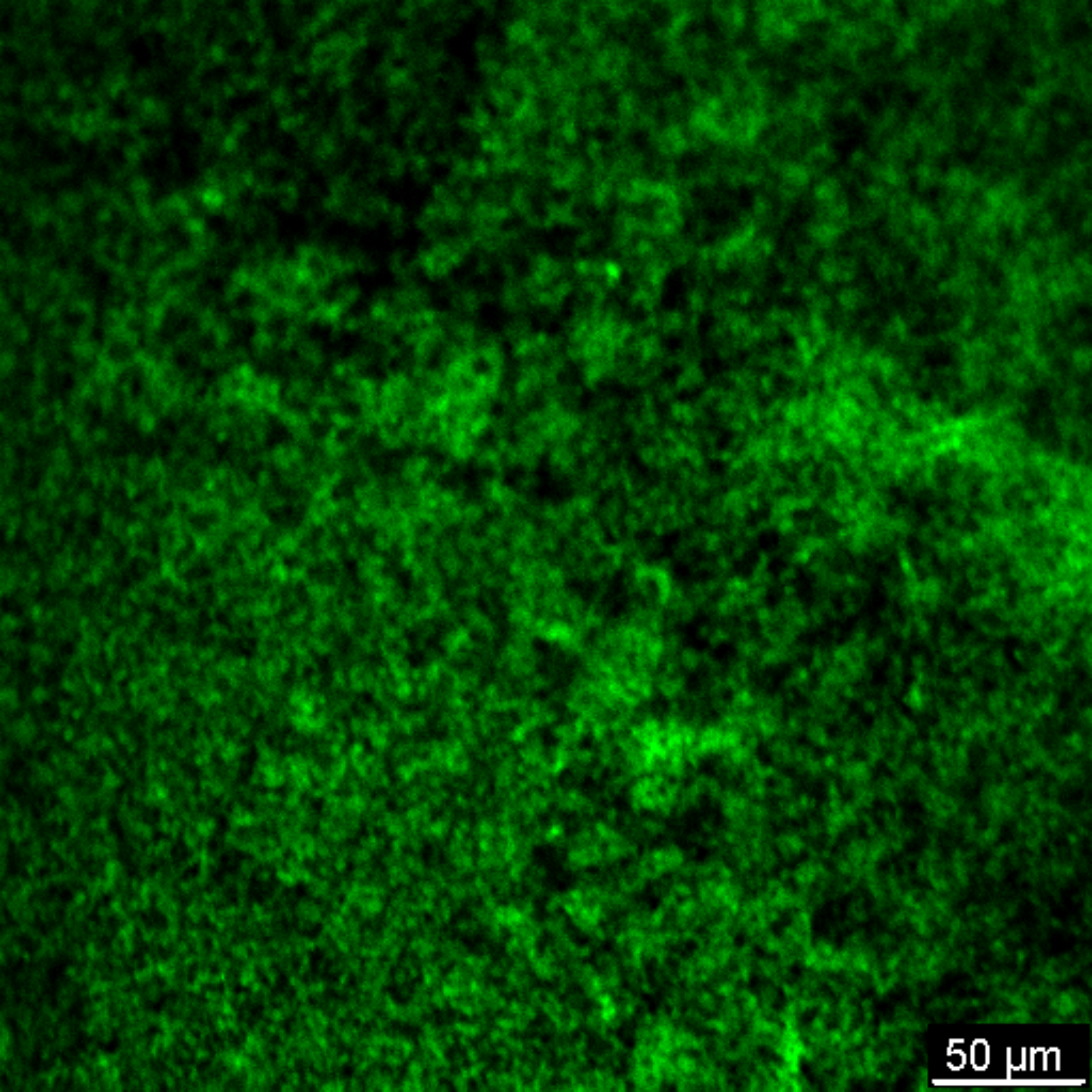
50 µm

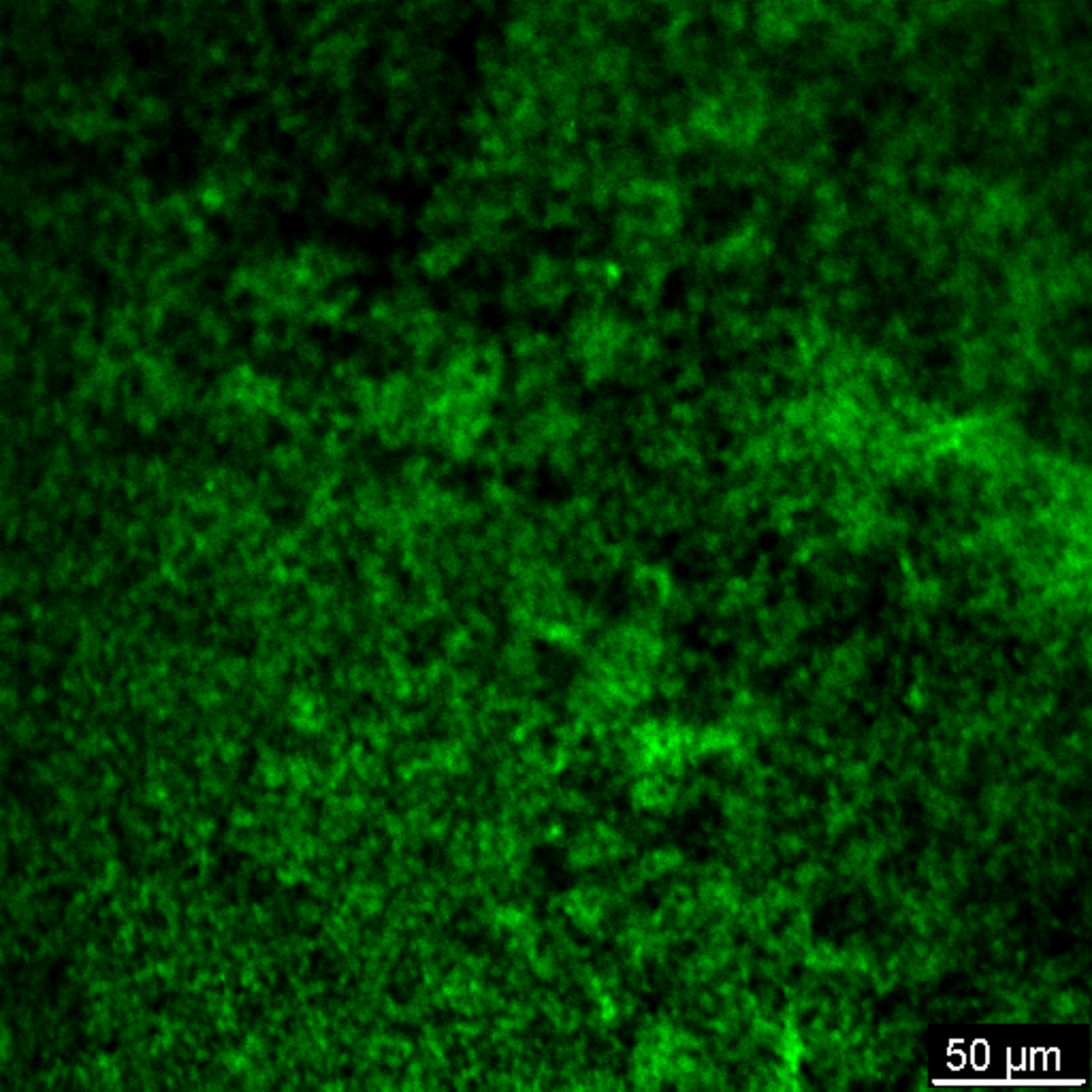
50 µm

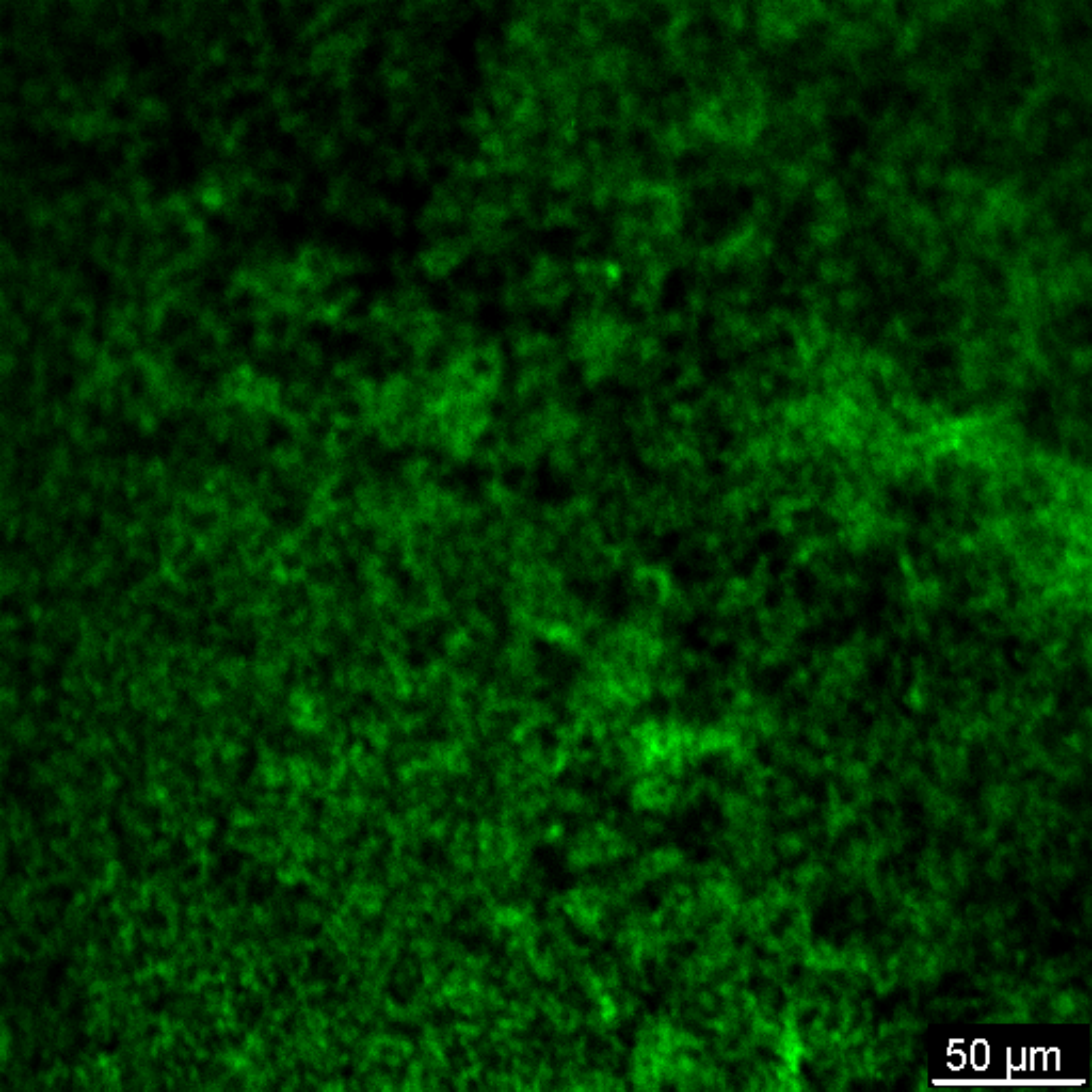
50 µm

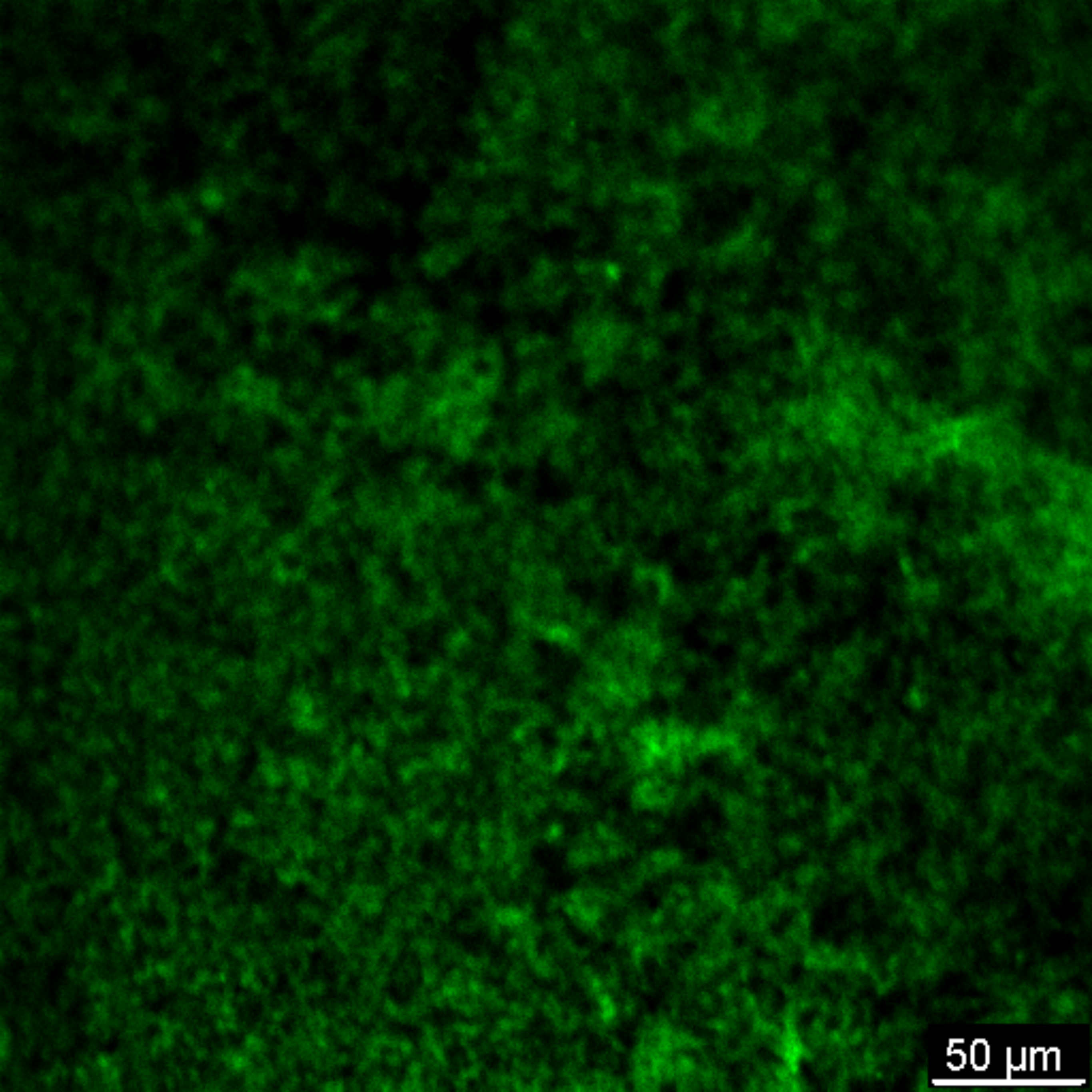
50 µm

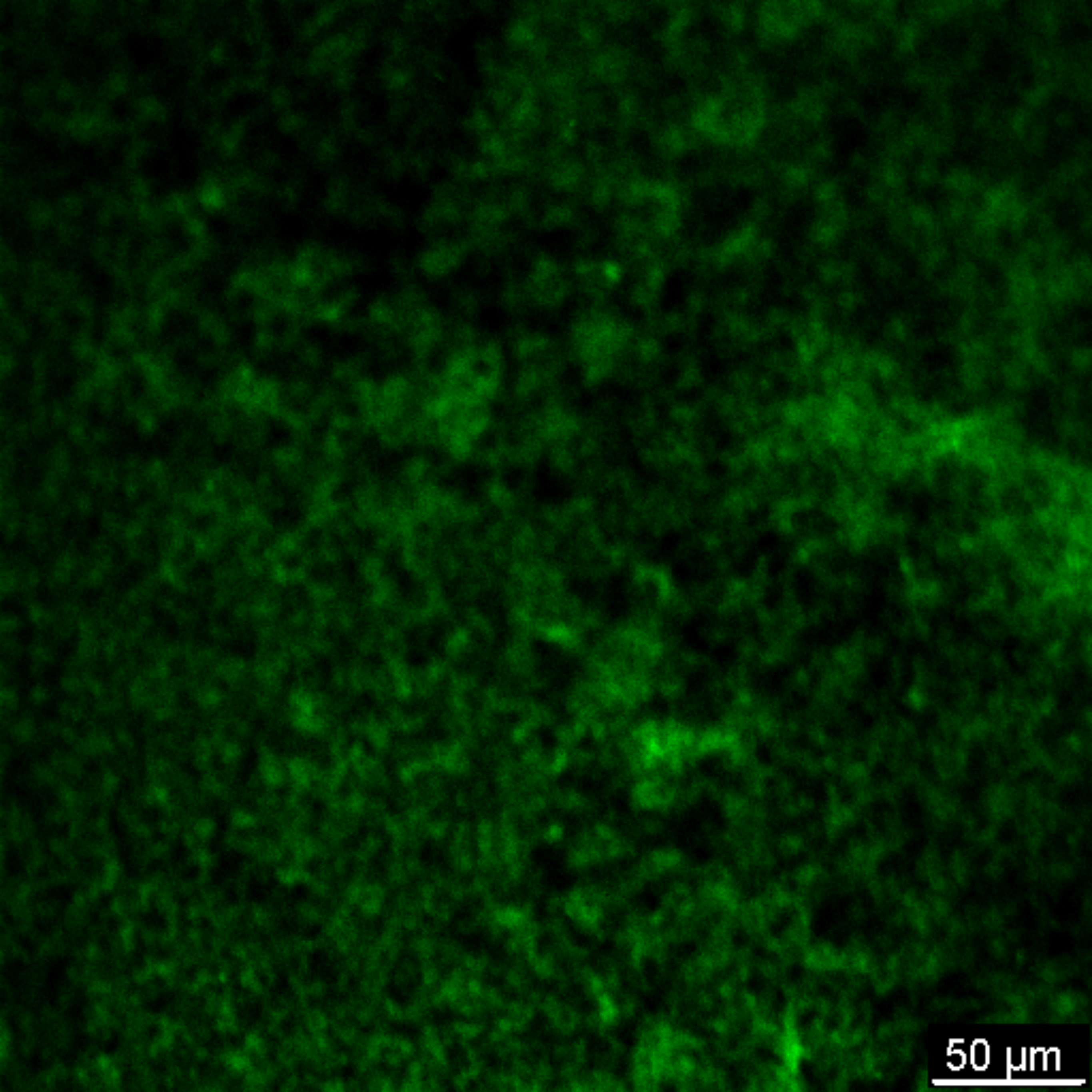
50 µm

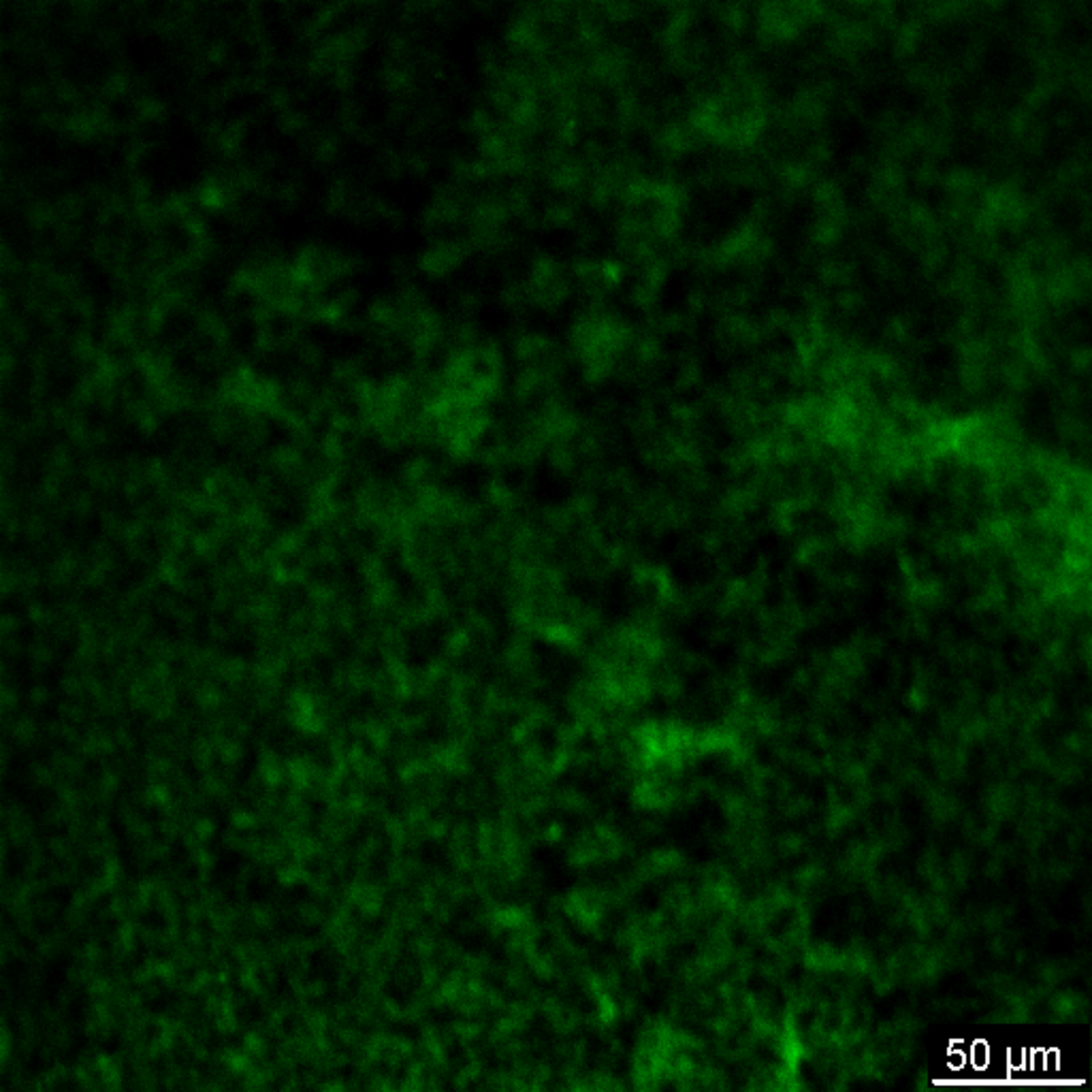
50 µm

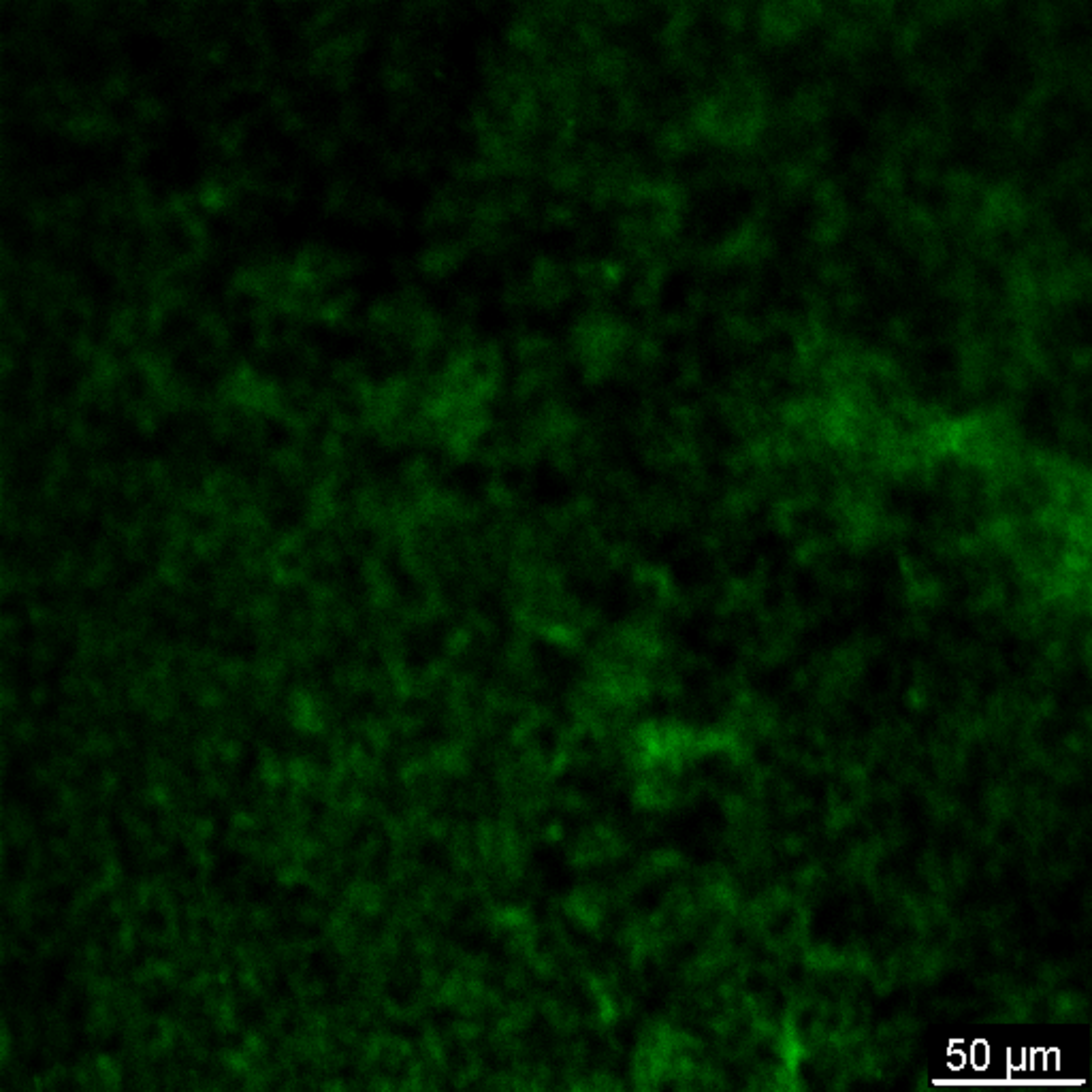
50 µm
