# Supplementary material for: Strengthening of enterococcal biofilms by Esp
Source: PLoS Pathog. 2022 Sep 14;18(9):e1010829. doi: 10.1371/journal.ppat.1010829 (PMC9512215; doi:10.1371/journal.ppat.1010829)
Supplement: Attachment — Submitted filename: Response.docx [file ppat.1010829.s024.docx]

We are grateful for the highly positive comments from reviewers regarding our manuscript. Below, we provide point-by-point answers to comments brought up by the reviewers. Our answers are in black and the reviewers’ comments in blue.

Reviewer #1: 1) Figure 1A: It is not clear what the difference is between Esp452 and EspDDDK until the very end of the Results section though it is mentioned earlier in the manuscript including in the Introduction. It would improve the clarity to mention what EspDDK is earlier and include it in it in Figure 1A.

We have revised Figure 1A (and its legend) to include the extent of the Esp_DDDK_ fragment.

2) Figure 3B: The legend picture in Figure 3B is not right – a gray bar is depicted where there should be white bar.

We have revised Figure 3B accordingly.

3) Figure 4C: The biofilm co-localization by Esp452 and Esp743 in Figure 4C shows just one image field is shown that could have been cherry-picked. A Mander’s overlap coefficient is mentioned, but there is no description. Can a more thorough description of what this is and how the data was analyzed be included in the results? Were multiple independent images analyzed? Can a Mander’s overlap coefficient be calculated for multiple fields and then averages calculated to assess how consistent the strength/weakness of the overlap is?

We have revised this section in the Materials and Methods and Results with a more detailed description of the calculation (which is more accurately called the “Manders coefficient” in the revised manuscript), and included its value from a second independent experiment. We have also provided the original citation for the Manders coefficient. The Manders coefficient is calculated for the entire Z-stack. In addition, we have included the entire Z-stacks for biofilms grown with PBS, Esp743, or Esp452 as supplemental files.

Materials and Methods:

Confocal images were obtained from each sample in two independent experiments with an average of 35 Z slices scanned with a 1.2 µm step size at a resolution of 512 x 512 pixels.

Manders coefficient M1 (25), which reports the ratio of the summed intensities from pixels in the red channel (protein) for which the intensity in the green channel (biofilm) was above zero to the total intensity in the red channel, was calculated for the entire stack with the ImageJ plug-in JACoP (26).

Results:

Fluorescence microscopy of fluorophore-labeled Esp fragments, which were added at the time of inoculation, revealed that Esp452, but not Esp743, colocalized with biofilms, which were detected using a nucleic acid-sensitive fluorophore (Fig. 4C and Supplemental Files 1, 2, and 3). Colocalization was confirmed quantitatively in two independent experiments, for which the Manders coefficient (25) was calculated to be 0.44 and 0.47 for Esp452, but only 0.03 in both experiments for Esp743.

4) Comment/question: Overexpression of the protein can cause the strengthening effect on the biofilms to occur at higher pHs. Do you think it is possible some strains of Enterococcus express more Esp than others contributing to natural strain variation and niche adaptation?

The possibility that natural variation occurs in Exp expression level among strains cannot be excluded. We have raised this possibility by adding the following sentence to the Discussion:

At the same time, these results also suggest that natural variation in Esp expression level could account for the differences observed for Esp action among strains.

5) Too much data is in the supplement, much of which is important to the story. Not one main figure has more than 4 panels and more of the relevant data could be added to the main figures to make the paper easier to read. I suggest moving S5 (the actual visualization of the differences in the biofilms strengthens the story as the ATP measurements are not standard to the field) S12, S15, S17 and S18.

Figures S5 and S12 have been moved such that they are Figures 2A and 6D, respectively, in the revised manuscript.

For Figures S15, S17, and S18 we chose to leave these as supplementary figures as they provide cautionary notes about the prior literature rather revealing a novel feature of Esp action.

Reviewer #2: 1) Could the authors specify which Corning 96-well plates were used (3595, 3596, etc) in the Methods section? The plate material and any surface treatments result in changes in hydrophobicity, which can impact biofilm formation.

We used Corning plate #3603, and this information has been added to the Materials and Methods section.

2) Figure 2A shows a low percentage of signal coming from planktonic vs biofilm cells. This is slightly surprising given the high number of cells in the planktonic fraction (typically around 10^9 CFU/mL). Does this mean that the CFU of biofilm cells > planktonic cells? Or perhaps that biofilm cells are more amenable to lysis with the CellTiter-Glo reagent?

The experiment reports the number of planktonic bacteria after the biofilm has formed and matured, and indeed, the number of bacteria in the biofilm fraction is greater than the number of bacteria in the planktonic fraction. This means that the great majority of bacteria that were planktonic prior to biofilm formation (i.e., 10^9^ CFU/mL) end up as part of the biofilm.

3) Line 437 – “Esp452 bound ThT maximally at pH 4.5 (Fig. 3D)” – the average values for Esp452 at pH 4.2 and 4.3 appear to be greater than the value at 4.5 (although not by much)

The reviewer is correct. The sentence has been revised: “Esp452 bound ThT at pH 4.5 and lower (Fig. 3D), …”.
